# Balancing the Efficiency and Synthetic Accessibility of Organic Solar Cells with Isomeric Acceptor Engineering

**DOI:** 10.1002/advs.202207678

**Published:** 2023-05-12

**Authors:** Qianguang Yang, Haiyan Chen, Jie Lv, Peihao Huang, Deman Han, Wanyuan Deng, Kuan Sun, Manish Kumar, Sein Chung, Kilwon Cho, Dingqin Hu, Haiyan Dong, Li Shao, Fuqing Zhao, Zeyun Xiao, Zhipeng Kan, Shirong Lu

**Affiliations:** ^1^ Chongqing Institute of Green and Intelligent Technology, Chongqing School University of Chinese Academy of Sciences (UCAS Chongqing) Chinese Academy of Sciences Chongqing 400714 P. R. China; ^2^ University of Chinese Academy of Sciences Beijing 100049 P. R. China; ^3^ Chongqing University Chongqing 400044 P. R. China; ^4^ Hoffmann Institute of Advanced Materials Shenzhen Polytechnic 7098 Liuxian Boulevard Shenzhen 518055 P. R. China; ^5^ Department of Material Science and Technology Taizhou University Taizhou 318000 P. R. China; ^6^ Institute of Polymer Optoelectronic Materials and Devices State Key Laboratory of Luminescent Materials and Devices South China University of Technology Guangzhou Beijing 510641 P. R. China; ^7^ Pohang Accelerator Laboratory Pohang University of Science and Technology Pohang 37673 North Korea; ^8^ Department of Chemical Engineering Pohang University of Science and Technology Pohang 37673 South Korea; ^9^ School of Physical Science and Technology Guangxi University Nanning 530004 P. R. China

**Keywords:** organic solar cell, small‐molecule acceptor, isomeric engineering, synthetic simplification, cost‐effectiveness

## Abstract

With the continuous development of organic semiconductor materials and on‐going improvement of device technology, the power conversion efficiencies (PCEs) of organic solar cells (OSCs) have surpassed the threshold of 19%. Now, the low production cost of organic photovoltaic materials and devices have become an imperative demand for its practical application and future commercialization. Herein, the feasibility of simplified synthesis for cost‐effective small‐molecule acceptors via end‐cap isomeric engineering is demonstrated, and two constitutional isomers, BTP‐*m*‐4Cl and BTP‐*o*‐4Cl, are synthesized and compared in parallel. These two non‐fullerene acceptors (NFAs) have very similar optoelectronic properties but nonuniform morphological and crystallographic characteristics. Consequently, the OSCs composed of PM6:BTP‐*m*‐4Cl realize PCE of 17.2%, higher than that of the OSCs with PM6:BTP‐*o*‐4Cl (≈16%). When ternary OSCs are fabricated with PM6:BTP‐*m*‐4Cl:BTP‐*o*‐4Cl, the averaged PCE value reaches 17.95%, presenting outstanding photovoltaic performance. Most excitingly, the figure of merit (FOM) values of PM6:BTP‐*m*‐4Cl, PM6:BTP‐*o*‐4Cl, and PM6:BTP‐*m*‐4Cl:BTP‐*o*‐4Cl based devices are 0.190, 0.178, and 0.202 respectively. The FOM values of these systems are all among the top ones of the current high‐efficiency OSC systems, revealing high cost‐effectiveness of the two NFAs. This work provides a general but accessible strategy to minimize the efficiency‐cost gap and promises the economic prospects of OSCs.

## Graphical abstract

Two constitutional isomers, namely BTP‐*m*‐4Cl and BTP‐*o*‐4Cl, are synthesized with shortened synthesis steps and excellent yields. Both BTP‐*m*‐4Cl and BTP‐*o*‐4Cl work well with PM6, enabling highly efficient organic solar cells. More importantly, the figure of merit values of the isomers are among the best high‐efficiency organic photovoltaic materials, revealing outstanding cost‐effectiveness for future large‐scale manufacture.

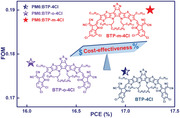

## 1 Introduction

Organic solar cells (OSCs) have been attracting broad research interests in recent decades due to their unique advantages of excellent solution processability, mechanical stability, and degradable active layer materials.^[^ ^1^ ^]^ The power conversion efficiencies (PCEs) of OSCs have been rapidly improved, benefiting greatly from the emergence of multifarious non‐fullerene acceptors (NFAs),^[^ ^2^ ^]^ such as the acceptor–donor–acceptor (A–D–A) type NFAs like ITIC, IT‐4F, and IT‐4Cl with seven‐fused‐ring IDTT central unit,^[^ ^3^ ^]^ the A–D–A′–D–A type NFAs like Y6, N3, BTP‐C*x*‐4Cl, BTP‐eC9, and L8‐BO containing N‐heterocyclic BTP central unit,^[^ ^4^ ^]^ and the porphyrin‐based acceptors like A1 and P‐*x* employing porphyrin ring and electron‐withdrawing terminal moieties.^[^ ^5^ ^]^ Although the PCEs of single‐junction OSCs involving these efficient NFAs have exceeded 19%, the construction of such molecular skeletons especially silk‐loop conjugated skeletons requires multi‐step synthesis and purification, resulting in low material yields and extremely high synthetic cost.^[^ ^6^ ^]^ Because of the limited production quantities and the undesired price of the efficient materials, the progress of large‐scale applications and commercialization is severely hindered.^[^ ^7^ ^]^ Hence, design and synthesis of the highly‐efficient and low‐cost small‐molecule NFAs are of great importance for developing the cost‐effectiveness of OSCs.^[^ ^8^ ^]^

Generally, there are two critical factors to improve cost‐effectiveness of OSCs: one is to simplify the synthetic complexity and reduce the price of materials; the other way is to increase the PCE of OSCs via innovation of materials and devices continuously.^[^ ^9^ ^]^ The price of materials can be reduced by simplifying the synthesis route and/or choosing cheaper raw materials. Over the past 5 years, a number of efficient molecular design strategies have been developed to improve performance and simplify the synthesis simultaneously, such as Cl‐substitution instead of F‐substitution strategies.^[^ ^10^ ^]^ F‐substitutional donors and acceptors have been widely used in highly efficient OSCs, while their complicated synthesis and low yields in the preparation process made the material price rather costly.^[^ ^11^ ^]^ Thus, Cl‐substitution arose and replaced F‐substitution for their easier chemical reaction and lower cost of raw material, in addition to their lower energy levels and the similar intermolecular non‐covalent interactions as F‐substitution.^[^ ^12^ ^]^ Hence, Cl‐substitution strategy is flourishing and contributes to many efficient NFAs with facile synthesis.

Isomeric engineering is a popular molecular design strategy for optimizing the structure and properties of photovoltaic materials. Isomers can be classified into two types: one is stereoisomer, and the other constitutional isomer.^[^ ^13^ ^]^ Isomeric NFAs with stereoisomer have the same molecular formula and molecular backbone (i.e., the identical connection order of atoms), but with atoms or atomic groups arranged differently in space.^[^ ^13^ ^]^ NFAs with constitutional isomers mean isomers that have the same molecular formula but inconsistency exists in covalently‐bonded atoms, such as carbon chain isomerism, position isomerism, and functional group isomerism.^[^ ^13^ ^]^ This concept has intrigued growing research interest in design of NFAs and has been proven to be an effective screening approach toward high‐performance photovoltaic materials.^[^ ^14^ ^]^ For example, Zhan and co‐workers developed FNIC1 and FNIC2,^[^ ^15^ ^]^ which are examples of constitutional isomers with the same end‐groups and side‐chains but isomeric fused‐ring cores; Li and co‐workers reported a side‐chain isomerization for ITIC by replacing 4‐hexylphenyl (*m*‐ITIC) with 3‐hexylphenyl;^[^ ^16^ ^]^ similarly, Wei and co‐workers shifted the position of the alkylthiolation on the phenyl group of the small‐molecule donor core (DTBDT) and fabricated OSC devices with PCE ranging from 11.9% (P‐PhS:BTP‐eC9 based devices) to 16.2% (M‐PhS:BTP‐eC9 based devices).^[^ ^17^ ^]^ These subtle changes in the two isomers influenced the electronic, optical, morphological, and charge‐transport properties, resulting in significantly enhanced PCEs.

In this work, we demonstrate the feasibility of simplified synthesis for cost‐effective small‐molecule acceptors via isomeric engineering. Based on the molecular skeleton of BTP‐4Cl,^[^ ^18^ ^]^ we develop two new constitutional isomers, namely BTP‐*m*‐4Cl and BTP‐*o*‐4Cl, using two new low‐priced end‐cap units, 2‐(4,6‐dichloro‐3‐oxo‐2,3‐dihydro‐1H‐inden‐1‐ylidene)malononitrile (IC‐m2Cl) and 2‐(4,5‐dichloro‐3‐oxo‐2,3‐dihydro‐1H‐inden‐1‐ylidene)malononitrile (IC‐o2Cl). The two end‐cap units are isomers of end group IC‐2Cl in Y7‐series, which are easily synthesized via two steps of chemical reaction and prepared with low‐priced raw materials. The two NFAs have very similar optoelectronic properties but differential morphological and crystallographic characteristics. The PM6:BTP‐*m*‐4Cl based devices produced an excellent PCE of over 17% both in chloroform (CHCl_3_) and *o*‐xylene solvent, higher than that of PM6:BTP‐*o*‐4Cl based OSCs. A further optimization of the ternary blends (PM6:BTP‐*m*‐4Cl:BTP‐*o*‐4Cl) achieved a best PCE of 17.95%, demonstrating outstanding photovoltaic performance of the developed NFAs. Moreover, the figure of merit (FOM) values of PM6:BTP‐*m*‐4Cl, PM6:BTP‐*o*‐4Cl, and PM6:BTP‐*m*‐4Cl:BTP‐*o*‐4Cl based devices are 0.190, 0.178, and 0.202, respectively, which are all among the top FOM values of the current high‐efficiency OSC system, revealing the high cost‐effectiveness of the two NFAs. The results demonstrate that developing constitutional isomers is an effective approach to fine tune molecular structure and performance, as well as to seek high cost‐effective photovoltaic materials for OSCs.

## 2 Results and Discussion

### 2.1 Molecular Design and Synthesis

**Figure 1:**
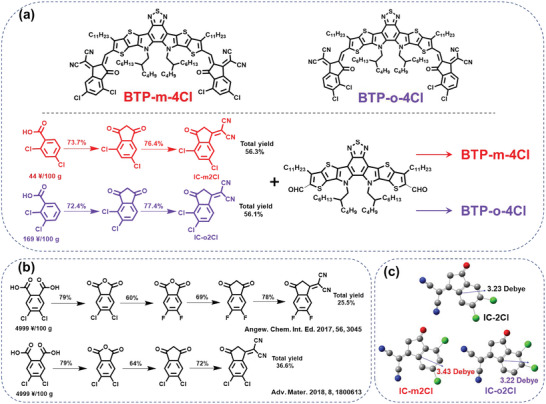
a) Molecular structures and synthetic routes of BTP‐*m*‐4Cl and BTP‐*o*‐4Cl; b) the reported synthetic routes of popular end groups IC‐2F and IC‐2Cl; c) the dipole moments of IC‐m2Cl and IC‐o2Cl calculated by density functional theory.

Taking into consideration the material price, reducing synthetic routes and use of low‐cost raw materials are the key factors for controlling the overall cost of final materials. Hence, we choose Cl‐substituted end groups rather than F‐substituted end groups, and used cheaper raw materials 2,4‐dichlorobenzoic acid (¥44/100 g) and 2,3‐dichlorobenzoic acid (¥169/100 g) instead of 4,5‐dichlorophthalic acid (¥4999/100 g) to design new end groups. By a two‐step process of chemical synthesis through Friedel–Crafts reaction and Knoevenagel condensation reaction, we obtained two new end groups IC‐m2Cl and IC‐o2Cl. IC‐m2Cl and IC‐o2Cl are constitutional isomers with different positions of the two Cl atoms, and both are isomers of common end group IC‐2Cl. Then, IC‐m2Cl and IC‐o2Cl incorporated with the central skeleton compound (BTP‐BO‐CHO) through further Knoevenagel condensation reaction to provide the final isomeric targets BTP‐*m*‐2Cl and BTP‐*o*‐2Cl. The molecular structures and synthetic routes of the corresponding materials are shown in **Figure** **1**a and Figure S1, Supporting Information. The detailed synthetic procedures and the structural characterizations (^1^H‐NMR, ^13^C‐NMR, and MS spectra) of the intermediate products and target molecules are described in Supporting Information (Figures S1–S15, Supporting Information).

Compared with the common end moieties (IC‐2F and IC‐2Cl) in previous reports (Figure 1a,b), the synthesis procedures of IC‐m2Cl and IC‐o2Cl are simplified to a two‐step process with cheaper raw precursors, while four‐step and three‐step process are needed for IC‐2F^[^ ^19^ ^]^ and IC‐2Cl,^[^ ^11^ ^]^ respectively. Even more, the yields of intermediate products and targets are good, especially the last step of BTP‐*m*‐2Cl and BTP‐*o*‐2Cl are in high yields of 95.4% and 96.2%, which are key factors for cutting the cost of organic materials. Therefore, BTP‐*m*‐2Cl and BTP‐*o*‐2Cl have significant advantages of low‐cost manufacturing compared with the previous reported Y6‐sieris NFAs containing IC‐2Cl or IC‐2F end groups.

Besides, we investigated dipole moments of the two moieties by density functional theory (DFT) calculations before synthesis. The dipole moments of IC‐m2Cl and IC‐o2Cl in the ground state are 3.43 and 3.22 Debye, respectively (Figure 1c). The dipole moment values and direction differ from IC‐2Cl (3.23 Debye, Figure 1c), bringing inconsistent molecular orientation and interaction force at solid‐state stacking.^[^ ^20^ ^]^ The similar values of dipole moments for IC‐o2Cl and IC‐2Cl indicate their well‐matched electronic‐drawing ability. The larger dipole moments of IC‐m2Cl portend its stronger electronic‐drawing ability compared to IC‐2Cl and IC‐o2Cl, which can potentially enhance the charge transfer, broaden the absorption spectrum, and form a more vital molecular packing simultaneously.

### 2.2 Photophysical and Electrochemical Properties

**Figure 2:**
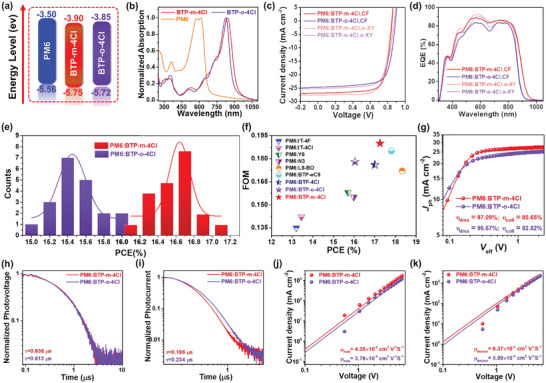
a) The energy levels and b) absorption spectra of PM6, BTP‐*m*‐4Cl, and BTP‐*o*‐4Cl; c) *J–V*, d) EQE and IQE of the optimal devices; e) statistical diagram of PCE distribution for 20 individual devices; f) summary of the PCE and FOM of different OSC systems; g) the *J*_ph_–*V*_eff_, h) TPV, i) TPC, j) hole‐mobilities, and k) electron‐mobilities of the optimal devices.

Cyclic voltammetry (CV) was used to study the electrochemical properties of BTP‐*m*‐2Cl and BTP‐*o*‐2Cl. The highest occupied molecular orbital (HOMO)/lowest unoccupied molecular orbital (LUMO) levels of BTP‐*m*‐2Cl and BTP‐*o*‐2Cl are −5.75/−3.90 and −5.72/−3.85 eV, respectively (**Table** **1** and **Figure** **2**a). The deeper energy levels of BTP‐*m*‐2Cl are finely fitting the tendency of DFT calculated results for terminal groups. It also demonstrates that end‐capped isomeric engineering with varying position of Cl atoms can effectively affect energy levels of NFAs. The UV–vis absorption spectra of BTP‐*m*‐2Cl and BTP‐*o*‐2Cl are shown in Table 1, Figure 2b, and Figure S17a, Supporting Information. The maximum absorption peaks of BTP‐*m*‐2Cl and BTP‐*o*‐2Cl in the film state are 840.5 and 825.6 nm, which red‐shift 87.4 and 80.0 nm from the maximum absorption peaks of the corresponding solution, respectively, indicating strong intermolecular interactions in solid films of the two molecules.^[^ ^21^ ^]^ The absorption edge of BTP‐*m*‐2Cl film is 930.5 nm, being with large red‐shift (37 nm) compared with BTP‐*o*‐4Cl film, implying a stronger ability of sunlight harvesting and electron attraction when the two Cl atoms are in the meta position.^[3a^ ^]^ That is, the position of Cl atoms on end‐cap is very important factor for their absorption and molecular stacking. Estimated from the absorption edges of the neat films, the optical band gaps of BTP‐*m*‐2Cl and BTP‐*o*‐2Cl are 1.33 and 1.39 eV, respectively, which are both in good accordance with the results measured by CV measurements.

**Table 1:** Summary of the photophysical and electrochemical properties of BTP‐*m*‐4Cl and BTP‐*o*‐4Cl

Acceptor	*E*_HOMO_ [ev]^cv^	*E*_LUMO_ [ev]^cv^	*λ*_ma_ *_x_* ^sol^ [nm]	*λ*_ma_ *_x_* ^film^ [nm]	*λ*_onset_^film^ [nm]	^a)^ *E*_g_^opt^ [ev]
BTP‐*m*‐4Cl	−5.75 (±0.05)	−3.90 (±0.05)	753.1	840.5	930.5	1.33
BTP‐*o*‐4Cl	−5.72 (±0.05)	−3.85 (±0.05)	745.6	825.6	893.5	1.39

^a)^ Calculation: *E*_g_^opt^ = 1240/*λ*_onset_^film^ (eV)

### 2.3 Photovoltaic Properties

To evaluate the photovoltaic performance of BTP‐*m*‐4Cl and BTP‐*o*‐4Cl, OSCs with conventional architecture of indium tin oxide (ITO)/2Br‐2Pac/active layer (PM6:BTP‐*m*‐4Cl or PM6:BTP‐*o*‐4Cl)/Phen‐NaDPO/Ag were fabricated. The details of optimized conditions and original experimental data are summarized in Tables S8–S13, Supporting Information. **Table** **2** summarizes the key photovoltaic parameters of the optimized PM6:BTP‐*m*‐4Cl and PM6:BTP‐*o*‐4Cl based devices. The *J–V* characteristics of optimized devices are plotted in Figure 2c and Figure S18a, Supporting Information. By using CHCl_3_ solvent, PM6:BTP‐*m*‐4Cl based devices achieved optimal PCE of 17.12% with an open‐circuit voltage (*V*_OC_) of 0.851 V, a short‐circuit current density (*J*_SC_) of 26.86 mA cm^−2^, and a fill factor (FF) of 74.72%; while PM6:BTP‐*o*‐4Cl based devices gained optimal PCE of 16.04%, accompanying with a higher *V*_OC_ of 0.890 V, a *J*_SC_ of 24.68 mA cm^−2^, and an FF of 72.99%. The diminishing *V*_OC_ (39 mV) of PM6:BTP‐*m*‐4Cl based devices compared to that of the PM6:BTP‐*o*‐4Cl based devices is mainly attributed to the deeper LUMO energy level of BTP‐*m*‐4Cl.^[^ ^22^ ^]^ Whereas, the higher *J*_SC_ of PM6:BTP‐*m*‐4Cl based devices compared to PM6:BTP‐*o*‐4Cl based devices indicates that PM6:BTP‐*m*‐4Cl based devices have higher photon utilization efficiency due to more red‐shifted absorption of PM6:BTP‐*m*‐4Cl blend film (Figure S17b, Supporting Information).^[^ ^23^ ^]^

**Table 2:** Photovoltaic parameters of the optimized PM6:BTP‐*m*‐4Cl and PM6:BTP‐*o*‐4Cl based devices

Materials	Solvent	*V*_OC_ [V]	*J*_SC_ [mA cm^−2^]	FF [%]	^a)^PCE [%]	^b)^ *J*_SC_ [mA cm^−2^]
PM6:BTP‐*m*‐4Cl	CHCl_3_	0.851 (0.850 ± 0.001)	26.86 (26.68 ± 0.19)	74.72 (73.09 ± 1.62)	17.12 (16.59 ± 0.53)	26.30
PM6:BTP‐*m*‐4Cl	*o*‐xylene	0.847 (0.843 ± 0.004)	27.68 (27.46 ± 0.22)	73.57 (72.43 ± 1.14)	17.25 (16.79 ± 0.46)	27.34
PM6:BTP‐*o*‐4Cl	CHCl_3_	0.890 (0.884 ± 0.005)	24.68 (24.44 ± 0.24)	72.99 (71.37 ± 1.61)	16.04 (15.44 ± 0.59)	24.04
PM6:BTP‐*o*‐4Cl	*o*‐xylene	0.880 (0.881 ± 0.001)	25.23 (25.39 ± 0.16)	71.36 (68.60 ± 2.75)	15.82 (15.35 ± 0.47)	25.19

^a)^ The average parameters were calculated with over 15 independent cells;

^b)^ *J*_SC_ integrated from the EQE curves.

Meanwhile, we compared PM6:BTP‐*m*‐4Cl and PM6:BTP‐*o*‐4Cl based devices in non‐halogen solvent. When the processing solvent changed to *o*‐xylene instead of CHCl_3_, the optimal PM6:BTP‐*m*‐4Cl based devices gained further enhancement and photovoltaic performance of 17.25% efficiency with higher *J*_SC_ were achieved (Table 2). On the contrary, PM6:BTP‐*o*‐4Cl based devices processed with *o*‐xylene solvent obtained optimal devices with only 15.82% efficiency. The corresponding parameters and *J–V* curves are shown in Table 2, Figure 2c, and Figure S18c, Supporting Information. Notably, the modified acceptor BTP‐*m*‐4Cl with *meta*‐dichloro substitutions on end groups possesses better photovoltaic performance relative to BTP‐*o*‐4Cl whether in CHCl_3_ or in *o*‐xylene solvent system.

The external quantum efficiency (EQE) curves of PM6:BTP‐*m*‐4Cl and PM6:BTP‐*o*‐4Cl based devices are shown in Figure 2d. The EQE curves of the two systems are different in shape, and the EQE maximum of PM6:BTP‐*m*‐4Cl based device is 88.75% at 560 nm, obviously higher than that of the PM6:BTP‐*o*‐4Cl device (83.55% at 560 nm). It is as well observed that the photo response of PM6:BTP‐*m*‐4Cl based devices is remarkably stronger in the range of 450 to 840 nm. The average EQE of PM6:BTP‐*m*‐4Cl based devices is 84.21%, higher than that of PM6:BTP‐*o*‐4Cl devices at the same region (79.02% in the range of 460–840 nm), which means BTP‐*m*‐4Cl can trigger the photon to electron conversion from PM6 more easily. It is further verified by a photoluminescence (PL, Figure S19, Supporting Information) study that the quenching efficiency of PM6 emission in the PM6:BTP‐*m*‐4Cl films (96.3%) is higher than that of PM6:BTP‐*o*‐4Cl films (92.3%), which also means the easier and faster exciton dissociation for PM6:BTP‐*m*‐4Cl blended system.^[^ ^24^ ^]^ The integrated *J*_SC_ calculated by EQE curves of PM6:BTP‐*m*‐4Cl and PM6:BTP‐*o*‐4Cl based devices are 26.30 and 24.04 mA cm^−2^, respectively, both of which are consistent with the values measured by solar simulator. Meanwhile, the uniform tendency of internal quantum efficiency (IQE) for the two systems in the range of 450–870 nm further verifies the higher photon to electron conversion efficiency in the PM6:BTP‐*m*‐4Cl devices (Figure S18b, Supporting Information).

Figure 2e shows the histograms and corresponding Gaussian distributions of PCE counts (20 individual devices) for the two systems along with CHCl_3_ solvent, indicating the good reproducibility of PM6:BTP‐*m*‐4Cl and PM6:BTP‐*o*‐4Cl based devices. Indeed, it reveals that both BTP‐*m*‐4Cl and BTP‐*o*‐4Cl are promising NFAs for high performance OSCs. Moreover, in comparison with the reported NFAs BTP‐4Cl, which has the same center skeleton but isomeric terminal groups, BTP‐*m*‐4Cl and BTP‐*o*‐4Cl were obtained by a simplified synthetic process with cheaper and available raw materials as well as high yields, portending excellent commercial potential, which will be discussed in detail below.

### 2.4 Cost‐Effectiveness Analysis

The PCE values and manufacturing costs of materials and OSCs are key parameters for consideration of their future commercial application. It is meaningless to evaluate commercial potential using only PCE value or synthetic cost of single donor or acceptor material.^[^ ^9^ ^]^ Currently, reports of cost‐effectiveness analysis are mostly based on calculation of synthetic complexity (SC) for materials and FOM values for OSCs.^[^ ^9^, ^25^ ^]^ The SC value gives a visual indication of the complexity degree of photovoltaic materials, that the higher SC index represents the more complicated synthetic process.^[25c^ ^]^ The FOM value is comprehensive index considering complexity degree of materials synthesis and its initial performance of OSC device.^[25a]^ It is a rough but reasonable index for evaluating the accessibility and commercial potential of photovoltaic materials applied in OSC manufacturing.^[25a]^

In this paper, we first calculated the synthetic complexity (SC) of the reported representative NFA acceptors (**Table** **3**) following Equation (1).^[^ ^9^, ^25^ ^]^

(1) $$\begin{array}{ccc} \mathrm{SC} & = & 35\frac{\mathrm{NSS}}{{\mathrm{NSS}}_{\max}}+25\frac{\log\left(\mathrm{RY}\right)}{\log\left({\mathrm{RY}}_{\max}\right)}+15\frac{\mathrm{NUO}}{{\mathrm{NUO}}_{\max}}+15\frac{\mathrm{NCC}}{{\mathrm{NCC}}_{\max}}\\ & & +\;10\frac{\mathrm{NHC}}{{\mathrm{NHC}}_{\max}} \end{array}$$

where NSS is the number of synthetic steps, RY is the reciprocity yields, NUO is the number of unit operations required for the isolation/purification, NCC is the number of column chromatography, NHC is the number of hazardous chemicals used for their preparation, and NSS_ma_ *_x_*, RY_ma_ *_x_*, NUO_ma_ *_x_*, NCC_ma_ *_x_*, and NHCmax are the maximum values of the corresponding parameters. From the comparison result (Table 3 and Table S6, Supporting Information), we can see the SC indexes of BTP‐*m*‐4Cl and BTP‐*o*‐4Cl are 81.562 and 81.546, respectively, lower than the SC indexes (90–110) of current high‐efficiency NFA acceptors, indicating their advantage of synthetic convenience. Then, to get a reasonable FOM value, SC indexes were calculated by a specific combination of OSC active layer, being with Equation (2).^[^ ^9^ ^]^

(2) $${\mathrm{SC}}_{D:A}=\frac{{\mathrm{SC}}_{D}\times W_{D}+{\mathrm{SC}}_{A}\times W_{A}}{W_{D}+W_{A}}$$

where SC_D_ and SC_A_ are the synthetic complexity of donor and acceptor independently, *W*_D_ and *W*_A_ are the weight ratio of donor and acceptor. Finally, the FOM values of devices were calculated by SC_D:A_ and PCE.^[^ ^25a^ ^]^

(3) $$\mathrm{FOM}=\frac{\mathrm{PCE}}{{\mathrm{SC}}_{D:A}}$$

**Table 3:** Summary of SC and correlation indexes of representative NFA acceptors

Material	NSS	RY	NUO	NCC	NHC	SC	Refs.
Y6	15	0.0104	26	6	25	100.402	[26]
N3	15	0.0057	26	6	25	105.450	[27]
L8‐BO	17	0.0129	32	10	29	111.524	[28]
BTP‐eC9	14	0.0184	24	6	23	92.616	[29]
BTP‐4Cl	14	0.0184	24	6	23	92.616	[30]
BTP‐*m*‐4Cl	13	0.0508	23	6	21	81.562	This Work
BTP‐*o*‐4Cl	13	0.0509	23	6	21	81.546	

Based on the initial reported performances, the FOM values of BTP‐*m*‐4Cl:Y6, BTP‐*o*‐4Cl:Y6, and current high‐efficiency OSCs systems have been calculated. The FOM values of PM6:BTP‐*m*‐4Cl and PM6:BTP‐*o*‐4Cl based devices are 0.190 and 0.178, respectively, both among the top FOM values of the current high‐efficiency binary OSCs, which reveal the higher cost‐effectiveness of the two new NFAs (**Table** **4** and Figure 2f). We also calculated the FOM indexes of other representative OSC system with active layer of polymer:PCBM, polymer:ITIC, and polymer:IT‐4F (Table S7, Supporting Information), the results demonstrate that BTP‐*m*‐4Cl and BTP‐*o*‐4Cl have good advantage of synthesis and application potential in future OSC device manufacture. From another perspective, both SC and FOM indexes of BTP‐*m*‐4Cl and BTP‐*o*‐4Cl are superior to those of the original isomer BTP‐4Cl, indicating isomeric engineering is an effective approach to develop low‐cost materials in the future.

**Table 4:** Summary of PCE, SC, and FOM indexes of representative OSC devices

Material	Ratio_D:A_	SC_D:A_	PCE [%]	FOM	Refs.
PM6:Y6	1:1.2	99.641	15.7	0.158	[26]
PM6:N3	1:1.2	103.331	15.98	0.155	[27]
PM6:L8‐BO	1:1.2	106.643	18.32	0.172	[28]
PM6:BTP‐eC9	1:1.2	96.330	17.8	0.185	[29]
PM6:BTP‐4Cl	1:1.2	96.330	17.0	0.176	[30]
PM6:BTP‐*m*‐4Cl	1:1.3	89.921	17.25	0.190	This Work
PM6:BTP‐*o*‐4Cl	1:1.3	89.912	16.04	0.178	
PM6:BTP‐*m*‐4Cl:BTP‐*o*‐4Cl	1:1:0.3	89.023	17.95	0.202	

### 2.5 Charge Carrier Dynamics Analysis

To understand the discrepancy of *J*_SC_, FF, and PCE, we turn to discuss the exciton dissociation, recombination, and charge carrier dynamics of the PM6:BTP‐*m*‐4Cl and PM6:BTP‐*o*‐4Cl based devices. Figure 2g depicts the photocurrent density (*J*_ph_) as a function of the effective voltage (*V*_eff_) to study the charge generation and extraction properties. The *J*_ph_ *= J*_L_ − *J*_D_, in which *J* is the current density and the subscripts represent the test under illumination (L) or in the dark (D); the *V*_eff_ *= V*_0_ *− V*_A_, where *V*_0_ is the voltage when *J*_ph_ is equal to 0 and *V*_A_ is the applied bias voltage.^[^ ^31^ ^]^ At high *V*_eff_ of 2.5 V, all the photogenerated excitons are assumed to be dissociated into free charge carriers and collected by electrodes, and so *J*_ph, sat_ is obtained (corresponding data in Table S1, Supporting Information). The exciton dissociation efficiency (*η*_diss_ = *J*_SC_/*J*_ph, sat_) and charge collection efficiency (*η*_coll_ = *J*_ma_ *_x_*_power_/ *J*_ph, sat_) are calculated under the short circuit and maximum power output conditions, respectively. The PM6:BTP‐*m*‐4Cl based device exhibits a saturated current density (*J*_ph,sat_) of 27.73 mA cm^−2^, higher than the PM6:BTP‐*o*‐4Cl based device (25.30 mA cm^−2^), which is in line with better photon harvest capability of PM6:BTP‐*m*‐4Cl based device.^[^ ^31^ ^]^ The PM6:BTP‐*m*‐4Cl based device exhibits a *η*_diss_ of 97.09% and a *η*_coll_ of 85.65%, higher than the PM6:BTP‐*o*‐4Cl based device (*η*_diss_ of 96.67% and *η*_coll_ of 82.82%). Therefore, a good exciton dissociation and more appropriate carrier collection path exist in PM6:BTP‐*m*‐4Cl based devices, which are essential to give a higher *J*_SC_ opportunity and improve the final efficiency.

The bimolecular charge recombination losses could be qualitatively analyzed by employing the power–law relation of *J*_SC_∝*I* ^*α*^ to fit *J*_SC_ as a function of the incident light intensity plotted in log scales.^[^ ^31^ ^]^ That is, *α* is the power factor (0 < *α* ≦ 1), and if the value of *α* is 1, it means nearly all free carriers are swept out and collected at the electrodes prior to recombination.^[^ ^31^, ^32^ ^]^ From the fitting curves (Figure S21a, Supporting Information), the calculated *α* values of PM6:BTP‐*m*‐4Cl and PM6:BTP‐*o*‐4Cl based devices are 0.988 and 0.980, respectively, which indicate PM6:BTP‐*m*‐4Cl based device is less affected by bimolecular recombination than PM6:BTP‐*o*‐4Cl based device. Trap assisted charge recombination is another key factor affecting the charge carrier behaviors, which reflects the extent of carrier traps across the active layer or at the interface between the organic semiconductor and the electrode. When *V*_OC_ is plotted as a function of the incident light intensity, the data are fitted according to the expression *V*_OC_∝*nkT*/*q*ln(*I*), where *n*, *k*, *T*, and *q* are the ideality factor, Boltzmann constant, the temperature in Kelvin, and elementary charge, respectively.^[^ ^31^ ^]^ The *n* value is usually in the range of 1 to 2, where a value of 1 stands for a trap‐free condition and a value more than 1 stands for trap‐assisted recombination.^[^ ^31^, ^32^ ^]^ As fitted in Figure S21b, Supporting Information, the *n* values of PM6:BTP‐*m*‐4Cl and PM6:BTP‐*o*‐4Cl based devices are resulted as 1.34 and 1.40 respectively, indicating PM6:BTP‐*m*‐4Cl based device suffers from slighter trap‐assisted recombination, which accounts for the promoted charge extraction and improved photovoltaic performance.

To quantitative analysis of the charge extraction and recombination patterns of PM6:BTP‐*m*‐4Cl and PM6:BTP‐*o*‐4Cl based devices, we further performed transient photovoltage (TPV) and photocurrent (TPC) (The original data are provided in Figure S22, Supporting Information). The carrier lifetimes (*τ*) under open‐circuit conditions are extracted from the TPV decay dynamics employing mono‐exponential fit (Figure 2h). The PM6:BTP‐*m*‐4Cl based device exhibits a *τ* value of 0.638 µs, longer than its counterpart of PM6:BTP‐*o*‐4Cl based device (0.613 µs), which is in compliance to the above result of lower recombination for PM6:BTP‐*m*‐4Cl based device. The photocurrent decay time of PM6:BTP‐*m*‐4Cl and PM6:BTP‐*o*‐4Cl based devices are 0.196 and 0.234 µs as depicted in TPC (Figure 2i), indicating PM6:BTP‐*m*‐4Cl based device has more effective charge carrier extraction. Meanwhile, the reduced charge extraction time agrees with the abovementioned higher charge collection efficiency (*η*_coll_ = 85.85%) in PM6:BTP‐*m*‐4Cl based device.^[^ ^31^, ^33^ ^]^ Consequently, the faster carrier extraction and less extent of free charge recombination produce synergistic effects of the higher *J*_sc_ and FF in PM6:BTP‐*m*‐4Cl based device.^[^ ^31^, ^32^ ^]^

Last, the hole‐ and electron‐mobilities are derived by fitting the dark current density with the space charge limited current model. The hole‐only diode devices were fabricated with the device architectures of ITO/ MoO_3_/active layer/MoO_3_/Ag and electron‐only diode devices with ITO/ZnO/PhenoNaDPO/active layer/PhenoNaDPO/Ag. As shown in Figure 2j,k and Table S2, Supporting Information, the hole‐ and electron‐mobility of PM6:BTP‐*m*‐4Cl based devices are 4.35 × 10^−4^ and 6.37 × 10^−4^ cm^2^ V^−1^ s^−1^, respectively, higher than values of PM6:BTP‐*o*‐4Cl based devices (3.76 × 10^−4^ and 5.99 × 10^−4^ cm^2^ V^−1^ s^−1^). The higher and more balanced electron/hole mobilities of PM6:BTP‐*m*‐4Cl based devices (*µ*_e_/*µ*_h_ = 1.46, contrast to 1.60 for PM6:BTP‐*o*‐4Cl based devices), which conform to the less recombination, more fluent charge extraction, and collection discussed above, drive higher *J*_sc_, FF, and PCE values of the corresponding devices.^[^ ^31^, ^32^, ^34^ ^]^ The results also indicate that the *meta*‐dichloro–substituted end group strategy can maximize and balance exciton dissociation and charge collection.

### 2.6 Morphological Characteristics

Grazing‐incidence wide‐angle X‐ray scattering (GIWAXS) was carried out to study the molecular packing and orientation in thin films. As displayed in Figure S23a,b, Supporting Information, dominantly face‐on oriented *π*–*π* stacking can be observed both in BTP‐*m*‐4Cl and BTP‐*o*‐4Cl neat films, while BTP‐*m*‐4Cl neat film has a stronger (010) *π*–*π* stacking diffraction than BTP‐*o*‐4Cl neat film. Estimated by the Scherrer equation,^[^ ^35^ ^]^ it clearly reveals a shorter (010) *π*–*π* stacking distance and a larger crystalline coherence length (CCL_010_) of the BTP‐*m*‐4Cl film (Table S3, Supporting Information), which evidence the closer packing of BTP‐*m*‐4Cl molecules and a better face‐on orientation.

**Figure 3:**
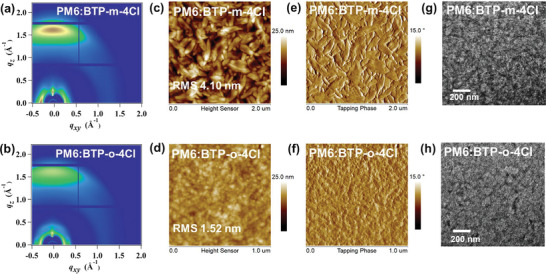
a,b) 2D GIWAXS patterns, c,d) AFM height images, e,f) phase images, and g,h) TEM images of PM6:BTP‐*m*‐4Cl and PM6:BTP‐*o*‐4Cl blended films.

For the blended films processed under optimal device conditions, the PM6:BTP‐*m*‐4Cl and PM6:BTP‐*o*‐4Cl blended films demonstrate similar molecule orientation in **Figure** **3**a,b, and line cut profiles along the direction of OOP and IP have been collected in Table S3, Supporting Information. The PM6: BTP‐*m*‐4Cl blended film displays (100) lamellar peaks at *q_xy_* ≈ 0.304 Å^−1^ with CCL of 8.31 nm (Figure S23 and Table S3, Supporting Information). In comparison, the PM6:BTP‐*o*‐4Cl blended film counterpart exhibits (100) lamellar peaks at *q_xy_* ≈ 0.291 Å^−1^ with CCL of 8.44 nm. The *π*–*π* stacking diffraction peak of PM6:BTP‐*m*‐4Cl blended film in the OOP direction is located at *q_z_* ≈ 1.660 Å^−1^ with a d‐spacing of 3.785 Å and a CCL of 2.20 nm, whereas the parallel *π*–*π* stacking diffraction peak of PM6:BTP‐*o*‐4Cl blended film shifts to 1.653 Å^−1^ with a *d*‐spacing of 3.801 Å and a CCL of 1.99 nm. The shorter *d*‐spacing and longer CCL values imply PM6:BTP‐*m*‐4Cl blended films have a larger interplanar spacing and higher crystallinity. The higher crystallinity and more ordered arrangement for the crystal of BTP‐*m*‐4Cl molecules are also verified by more facile cultivation processes of single crystal (The single crystal diffraction data and perspective drawing of BTP‐*m*‐4Cl are shown in Table S5 and Figures S27–S29, Supporting Information). Indeed, we cultivated a single crystal of BTP‐*m*‐4Cl by solvent diffusion method facilely, but we could not gain the single crystal of BTP‐*o*‐4Cl with a similar method or other ways. Then, the higher crystallinity contributes to more appropriate phase aggregation, which not only facilitates charge transport but also reduces the trapping probability of the charge carrier and then prolongs the charge carrier lifetime.

Taking the above‐mentioned factors into consideration, we investigate the morphology of PM6:BTP‐*m*‐4Cl and PM6:BTP‐*o*‐4Cl blended films to study their phase separation and crystallinity. Atomic force microscopy (AFM) of pure and blended film has been investigated (Figure 3c,f and Figure S24, Supporting Information). As AFM height images demonstrated, PM6:BTP‐*m*‐4Cl blended film forms more visible surface pattern than PM6:BTP‐*o*‐4Cl blended films, where rod‐like microcrystalline texture and block structure can be clearly seen on the film surface, forming a dense and compact morphology, implying enhanced molecular aggregation with excellent crystallinity.^[^ ^36^ ^]^ In contrast, the PM6:BTP‐*o*‐4Cl blended film forms a series of small spherical aggregate morphology. As a result, the PM6:BTP‐*m*‐4Cl device has a root mean square roughness (RMS) values of 4.10 nm in Figure 3c, which is larger than PM6:BTP‐*o*‐4Cl device counterpart in Figure 3d (RMS 1.52 nm). This further illustrates the *meta*‐ and *ortho*‐dichloro–substituted end group strategy can control the molecular aggregation, being in line with the trend of crystallinity ascertained by GIWAXS tests.

As demonstrated by the AFM phase image (Figure 3e,f), PM6:BTP‐*m*‐4Cl blended film exhibits a very interesting block structure and crystallites interlaced morphology, in which large block structure and the microcrystalline structure can be seen to closely attach onto or even embed to form a special area. We believe that this special morphology can provide a more effective path for charge separation and transport compared to ordinary PM6:BTP‐*o*‐4Cl blended film. In fact, comparing AFM images of BTP‐*m*‐4Cl and BTP‐*o*‐4Cl neat films, similar conclusions could be obtained. From Figure S24a,b, Supporting Information, the BTP‐*m*‐4Cl neat film has RMS value of 1.36 nm, which is smaller than that of BTP‐*o*‐4Cl neat film (RMS 5.38 nm). It is clearly seen that BTP‐*m*‐4Cl neat film forms a larger block continuous morphology and becomes homogeneously distributed, while BTP‐*o*‐4Cl neat film shows more prominent domains and rougher surface. From AFM phase image of the neat films (Figure S24c,d, Supporting Information), the BTP‐*m*‐4Cl film presents a more well‐proportioned and smoother phase image than BTP‐*o*‐4Cl film.

Furthermore, from the transmission electron microscopy (TEM) images (Figure 3g,h), we obtain the information that PM6:BTP‐*m*‐4Cl blended film has more uniform surface and larger domain size. Both the AFM and TEM images confirm that the variance of morphology characteristics exist in the two mixed system, even though there is only a tiny structure change in the two NFAs.^[^ ^37^ ^]^ In short, the *meta*‐dichloro–substituted end group strategy brings a reasonable crystal contact and high crystallinity, which contributes to the exciton dissociation and charge collection, endowing the higher *J*_sc_, FF, and PCE values of PM6:BTP‐*m*‐4Cl based OSCs.

### 2.7 Ternary OSCs

From above analysis, we can see that PM6:BTP‐*m*‐4Cl based devices have higher *J*_SC_ and FF, while PM6:BTP‐*o*‐4Cl based devices have higher *V*_OC_. AFM tests show larger crystalline domains in PM6:BTP‐*m*‐4Cl based devices contrast to PM6:BTP‐*o*‐4Cl based devices. GIWAXS measurements show that a face‐on molecular orientation exist in PM6:BTP‐*m*‐4Cl blended films, but inconsonant molecular orientation and arrangement coexist in PM6:BTP‐*o*‐4Cl blended films. In view of the isomer structure and high compatibility of BTP‐*m*‐4Cl and BTP‐*o*‐4Cl, we hope to combine the advantages of the two systems to improve the energy match, morphology, and carrier characteristics of the device, thus to further enhance PCE of OSCs.

**Figure 4:**
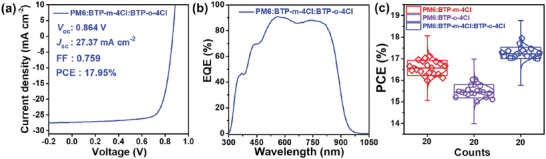
a) *J–V* and b) EQE curve of the optimal device based on PM6:BTP‐*m*‐4Cl:BTP‐*o*‐4Cl blended films; c) statistical diagram of PCE distribution for 20 individual devices.

With the total weight ratio of PM6:acceptor kept at 1:1.3, we adjusted the weight ratio of BTP‐*m*‐4Cl and BTP‐*o*‐4Cl systematically, and the detailed fabrication information can be also found in the Supporting Information (Tables S14–S24, Supporting Information). The *J–V*, EQE, and photovoltaic parameters of the optimized ternary OSCs are shown in **Figure** **4** and **Table** **5**. The results show that ternary OSCs with a weight ratio of 1:1:0.3 for PM6:BTP‐*m*‐4Cl:BTP‐*o*‐4Cl active layer can achieve a high PCE up to 17.95% (The certified PCE of National Institute of Metrology [NIM] is 17.4%, as shown in Figure S26, Supporting Information), which effectively enhance the performance relative to the corresponding binary OSCs. Compared to the binary OSCs, the enhanced *J*sc parameter is the key factor for performance improvement. The results clearly show the good compatibility and balance of isomeric acceptors in ternary OSCs. Although the characteristics of energy levels, absorption spectra, and molecular arrangements are slightly different; the energy matching, light absorption, and phase morphology of dual‐acceptor in ternary OSCs can be finely regulated.

**Table 5:** Photovoltaic parameters of the optimized PM6:BTP‐*m*‐4Cl:BTP‐*o*‐4Cl based ternary OSC devices

Materials	*V*_OC_ [mV]	*J*_SC_ [mA cm^−2^]	FF [%]	^a)^PCE [%]	^b)^ *J*_SC_ [mA cm^−2^]
PM6:BTP‐*m*‐4Cl:BTP‐*o*‐4Cl	0.864 (0.857 ± 0.007)	27.37 (26.99 ± 0.38)	75.90 (74.70 ± 1.21)	17.95 (17.27 ± 0.68)	27.14

^a)^ The average parameters were calculated with over 15 independent cells;

^b)^ *J*_SC_ integrated from the EQE curves.

Compared to binary PM6:BTP‐*m*‐4Cl based OSCs, the ternary device displays improvements not only with *J*_SC_ and FF but also for *V*_OC_. To evaluate the reason for this change, we took energy loss (*E*_loss_) analysis for the optimized binary and ternary devices. As reported, the total *E*_loss_ composed of three parts: the first part as ∆*E*1, radiative recombination loss above the bandgap calculated by equation $\Delta E_{1}=E_{g}-qV_{\mathrm{OC}}^{\mathrm{SQ}}$; the second part as ∆*E*2, radiative recombination loss below the bandgap also calculated by equation $\Delta E_{2}=qV_{\mathrm{OC}}^{\mathrm{SQ}}-qV_{\mathrm{OC}}^{\mathrm{rad}}$; and the third part as Δ*E*3, nonradiative recombination loss, also called *E*_loss,nr_, calculated by equation $\Delta E_{3}=qV_{\mathrm{OC}}^{\mathrm{non}-\mathrm{rad}}=-kTln\left({\mathrm{EQE}}_{\mathrm{EL}}\right)$.^[^ ^38^ ^]^ The details of each component of the *E*_loss_ are shown in Table S4 and Figure S25, Supporting Information. As shown in Table S4, Supporting Information, both binary and ternary devices exhibit similar values of Δ*E*1 about 0.262–0.263 eV. In contrast, a litter reducing Δ*E*2 for ternary and PM6:BTP‐*o*‐4Cl binary system compare to PM6:BTP‐*m*‐4Cl binary system, indicating lesser radiative recombination loss occurring in BTP‐*o*‐4Cl binary system. By the result of sensitive‐EQE values (Figure S25 and Table S4, Supporting Information), the ternary and binary devices with BTP‐m‐4C show enhanced EQE_EL_ relative to PM6:BTP‐*o*‐4Cl binary system, corresponding to a smaller ∆*E*3 compared to BTP‐*o*‐4Cl binary device. This result indicates that the nonradiative recombination loss in PM6:BTP‐*m*‐4Cl binary and PM6:BTP‐*m*‐4Cl:BTP‐*o*‐4Cl ternary devices was successfully suppressed. The total *E*_loss_ is 0.556, 0.548, and 0.542 eV for PM6:BTP‐*m*‐4Cl, PM6:BTP‐*o*‐4Cl, and PM6:BTP‐*m*‐4Cl:BTP‐*o*‐4Cl, respectively, which are very consistent with the *V*_OC_ tendency of the corresponding OSCs. Hence, a slightly lower *E*_loss_ for the optimal ternary system gave rise to a higher *V*_OC_.

Moreover, we took the FOM calculation of the ternary OSC device by Equation (3), the FOM value increased to 0.202, higher than the binary OSCs and other representative OSC systems (Table 4 and Table S7, Supporting Information). The prominent FOM value is among the best high‐efficiency organic photovoltaic materials, further confirming the high cost‐effectiveness of the two new NFAs applied in future OSC systems.

## 3 Conclusions

In summary, we report here a simple synthesis method for high‐efficiency NFA materials. First, two end groups (IC‐m2Cl and IC‐o2Cl) with different chlorine substitution positions have been developed by a two‐step process of chemical synthesis with cheap raw materials, and two isomeric acceptors (BTP‐*m*‐4Cl and BTP‐*o*‐4Cl) have been gained by shortened synthesis in high yields. The two acceptors are isomers, with slightly different energy levels, absorption spectra, and molecular arrangements. Application of the two isomeric acceptors in OSCs with PM6 as the donor material, BTP‐*m*‐4Cl displays better photovoltaic performance with a higher PCE over 17% both in CHCl_3_ and *o*‐xylene (non‐halogen) solvent. With dual‐acceptor system combined with the two isomeric acceptors, we built ternary OSCs based on PM6:BTP‐*m*‐4Cl:BTP‐*o*‐4Cl blended films, enabling PCE significantly increase to 17.95% (certified PCE of 17.4%). More importantly, we calculated the FOM values of the three devices, realizing 0.190, 0.178, and 0.202 for PM6:BTP‐*m*‐4Cl, PM6:BTP‐*o*‐4Cl, and PM6:BTP‐*m*‐4Cl:BTP‐*o*‐4Cl systems, respectively, which are all among the top ones of the current high‐efficiency OSC systems, revealing the outstanding cost‐effectiveness of the two acceptors for future manufacture. This work demonstrates that molecular isomerization strategy is an effective and facile method for manufacturing low‐cost and high‐efficiency NFA materials for OSCs.

## Conflict of Interest

The authors declare no conflict of interest.

## Author Contributions

Q.Y. and H.C. contributed equally to this work. Material synthesis and characterization, FOM calculations, writing and proof—Q.Y. and H.C.; device fabrication and testing—J.L. and Q.Y.; DFT calculations—P.H., Eloss analysis supporting—W.D., GIWAX testing supporting—M.K., S.C., and K.C.; AFM testing and other characterization—D.H., D. Hu, H.D., L.S., and F.Z.; supervision, resources and paper revision—K.S., Z.X., Z.K., and S.L.. All authors have read and agreed to the published version of the manuscript.

## Supporting information

Supporting Information

## Data Availability

The data that support the findings of this study are available in the supplementary material of this article.
