# Supplementary material for: Balancing the Efficiency and Synthetic Accessibility of Organic Solar Cells with Isomeric Acceptor Engineering
Source: Adv Sci (Weinh). 2023 May 12;10(20):2207678. doi: 10.1002/advs.202207678 (PMC10369256; doi:10.1002/advs.202207678)
Supplement: Supplementary file 1 — Supporting Information [file ADVS-10-2207678-s001.pdf]

ADVANCED SCIENCE
Open Access

Supporting Information

for *Adv. Sci.*, DOI 10.1002/advs.202207678

Balancing the Efficiency and Synthetic Accessibility of Organic Solar Cells with Isomeric Acceptor Engineering

Qianguang Yang, Haiyan Chen, Jie Lv, Peihao Huang, Deman Han, Wanyuan Deng, Kuan Sun, Manish Kumar, Sein Chung, Kilwon Cho, Dingqin Hu, Haiyan Dong, Li Shao, Fuqing Zhao, Zeyun Xiao, Zhipeng Kan* and Shirong Lu**

Supporting Information

Balancing the Efficiency and Synthetic Accessibility of Organic Solar Cells with Isomeric Acceptor Engineering

Qianguang Yang,[a,b,†] Haiyan Chen,[a,c,†] Jie Lv,[a,f] Peihao Huang,[a] Deman Han,[e] Wanyuan Deng,[h] Kuan Sun,[c] Manish Kumar,[g] Sein Chung,[i] Kilwon Cho,[i] Dingqin Hu, [a] Haiyan Dong,[a] Li Shao,[a,c] Fuqing Zhao,[a] Zeyun Xiao,[a,b,]* Zhipeng Kan,[d,]* and Shirong Lu[a,c,e]*

[a] Q. Yang,[†] H. Chen,[†] J. Lv, P. Huang, D. Hu, H. Dong, L. Shao, F. Zhao, Prof. Z. Xiao*, Prof. S. Lu*
Chongqing Institute of Green and Intelligent Technology, Chongqing School, University of Chinese Academy of Sciences (UCAS Chongqing), Chinese Academy of Sciences, Chongqing, 400714, China.
E-mail: lushirong@cigit.ac.cn, xiao.z@cigit.ac.cn

[b] Q. Yang,[†] Prof. Z. Xiao*
University of Chinese Academy of Sciences, Beijing 100049, China.

[c] H. Chen,[†] L. Shao, Prof. S. Lu*, Prof. K. Sun
Chongqing University, Chongqing 400044, China.

[d] Prof. Z. Kan*
School of Physical Science and Technology, Guangxi University, Nanning 530004, China.
E-mail: kanzhipeng@gxu.edu.cn

[e] Prof. S. Lu*, Dr. D. Han
Department of Material Science and Technology, Taizhou University, Taizhou 318000, China

[f] J. Lv
Hoffmann Institute of Advanced Materials, Shenzhen Polytechnic, 7098 Liuxian Boulevard, Shenzhen 518055, China.

[g] Dr. M. Kumar
Pohang Accelerator Laboratory, Pohang University of Science and Technology, Pohang, 37673, Korea

[h] Dr. W. Deng
Institute of Polymer Optoelectronic Materials and Devices. State Key Laboratory of Luminescent Materials and Devices, South China University of Technology Guangzhou 510641.P R. China

[i] Dr. S. Chung, K. Cho
Department of Chemical Engineering, Pohang University of Science and Technology, Pohang 37673, South Korea.

[†] These authors contributed equally to this work.

Contents

1. Synthesis 1
2. Characterization methods 3
3. Device fabrication and testing 4
4. Supplemental figures 6
5. Crystal data and structure of BTP-m-4Cl 21
6. The synthesis route of reported materials for SC calculation 24
7. Calculation of SC and FOM index 32
8. The detailed fabrication of OSC devices 34
9. References 40

1. Synthesis

Materials: PM6 was purchased from Organtec. Ltd. 12,13-bis(2-butyloctyl)-3,9-diundecyl-12,13-dihydro-[1,2,5]thiadiazolo[3,4-e]thieno[2",3":4',5']thieno[2',3':4,5]pyrrolo[3,2g]thieno[2',3':4,5]thieno[3,2-b]indole-2,10-dicarbaldehyde (BT-BO-CHO) was purchased from Jiangsu ji'a Biotechnology Co., Ltd. PEDOT:PSS (Clevios AL4083) was purchased from Xi’an Polymer Light Technology Corp. and the ITO glass was purchased from Advanced Election Technology Co. Ltd. Other reagents and solvents were purchased from Energy Chemical, Tansoole, Derthon OPV Co.Ltd and JiangSu GE-Chem Biotech. Ltd.

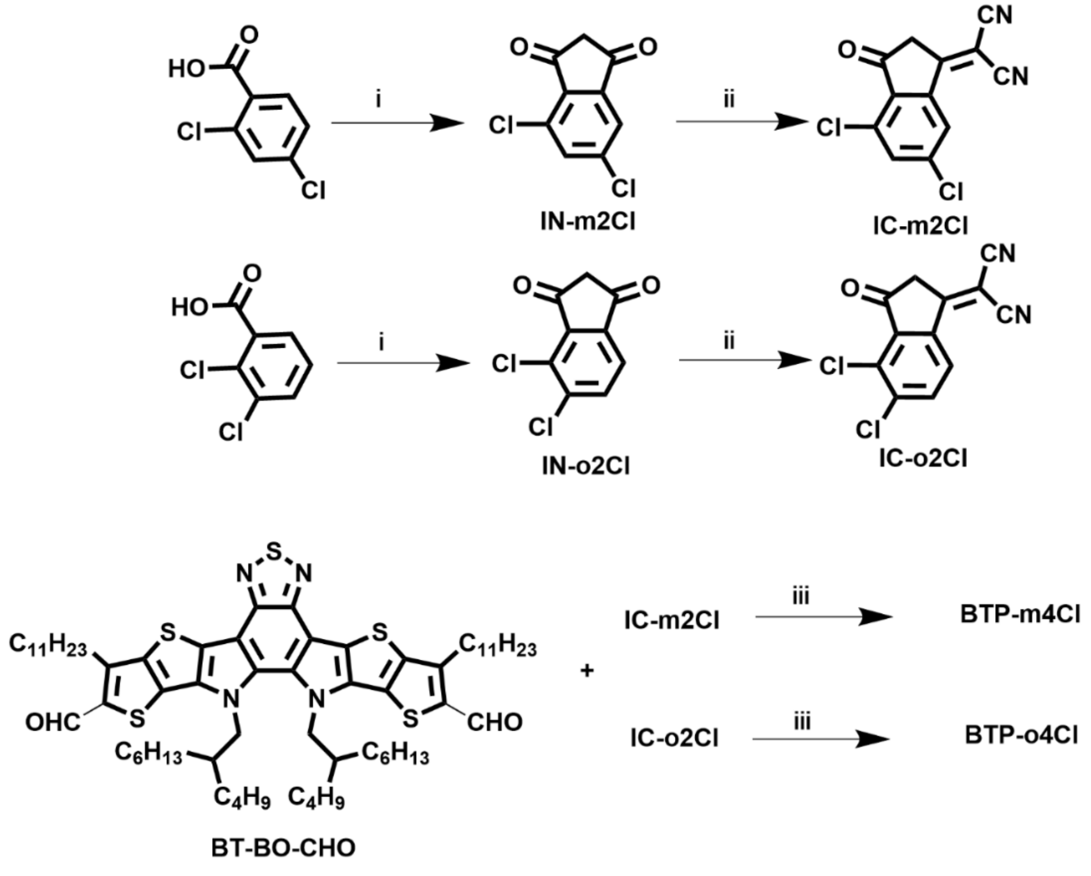

Figure S1. Synthetic route of BTP-m-4Cl and BTP-o-4Cl. (i) $SOCl_2$, 65°C 1 hour (h); $AlCl_3$, Malonyl chloride, 65°C, overnight; (ii) Malononitrile, NaOAc, room temperature 2 h; (iii) Pyridine, $CHCl_3$, room temperature 3 h.

Synthesis of IN-m2Cl: Thionyl chloride (4.4 ml g, 60 mmol) was added to a solution of 2,4-dichlorobenzoic acid (5.7300 g, 30 mmol) in $CHCl_3$ (30 mL) under the protection of N_2. Subsequently, DMF (0.5 mL) was added to initiate the reaction. The reaction was stirred at 65 ^oC for 1 hour, then the excess thionyl chloride was removed by under reduced pressure, and the intermediate

2,4-dichlorobenzoyl chloride was used for the next step without further purification. The mixture of malonyl dichloride (5.6 ml, 60 mmol) and $AlCl_3$ (5.9999 g, 45 mmol) in dry dichloromethane (30 mL) solution was flushed with N_2 for ten minutes, then 2,4-dichlorobenzoyl chloride was added. After being refluxed at 65 °C overnight. After cooling to room temperature, the mixture was poured into the oxalic acid aqueous solution of 2M slowly. Then, the PH of the solution was tuned to around 7 by using $NaHCO_3$ aqueous solution. The crude product extracted from dichloromethane, after removing the solvent, was purified by silica gel chromatography using dichloromethane as the eluent to obtain a yellow solid IN-m2Cl (4.7511 g, yield 73.7%). ^{1}H NMR (400 MHz, $CDCl_3$) δ 7.84 (d, 1H), 7.75 (d, 1H), 3.30 (s, 2H). ^{13}C NMR (101 MHz, $CDCl_3$) δ 194.66, 192.95, 145.76, 142.57, 136.90, 136.88, 132.82, 121.92, 45.45.

Synthesis of IC-m2Cl: Sodium acetate (0.5726 g, 7.0 mmol) was added to a solution of malononitrile (0.6144 g, 9.3 mmol) and IN-m2Cl (1.0000 g, 4.7 mmol) in 20 ml of ethanol. The mixture was stirred for 1 h at room temperature. Then 10 mL of water was added into the mixture, then acidified to pH 1-2 by using HCl (2 M). The solid was filtered and purified by column chromatography, and the target IC-m2Cl was obtained as a yellow solid (0.9343 g, yield 76.4%). ^{1}H NMR (400 MHz, $CDCl_3$) δ 8.53 (d, 1H), 7.76 (d, 1H), 3.78 (s, 2H). ^{13}C NMR (101 MHz, $CDCl_3$) δ 190.51, 163.07, 144.96, 142.91, 136.75, 134.23, 134.08, 124.34, 111.51, 111.43, 81.23, 77.36, 77.04, 76.72, 43.92.

Synthesis of BTP-m-4Cl: The compound BTP-BO-CHO (114.0 mg, 0.1 mmol), IC-m2Cl (105.2 mg, 0.4 mmol), and chloroform (10 mL) were added to a 25 mL round bottom flask. Then pyridine (0.5 mL) was added. The reaction mixture was stirred at room temperature (25-30°C) for 3 hours. Methanol was added, and the precipitate was collected by filtration to obtain the crude product. Using petroleum ether:dichloromethane (1:1) as the eluent, it was further chromatographed on a silica gel column to obtain the compound BTP-m-4Cl (163.0mg, yield 95.4%). ^{1}H NMR (400 MHz, $CDCl_3$) δ 9.17 (s, 2H), 8.62 (d, 2H), 7.66 (d, 2H), 4.86-4.74 (m, 4H), 3.22 (t, 4H), 2.18-2.11 (m, 2H), 1.91-1.83 (m, 4H), 1.54-1.47 (m, 4H), 1.43-1.35 (m, 4H), 1.33-0.90 (m, 56H), 0.87 (t, 6H), 0.73-0.62 (m, 12H). ^{13}C NMR (101 MHz, $CDCl_3$) δ 185.02, 157.69, 154.19, 147.48, 145.36, 143.01, 141.23, 137.82, 136.77, 135.77, 135.17, 134.38, 133.65, 133.04, 131.21, 130.11, 123.79, 120.05, 115.16, 114.63, 113.65, 68.65, 55.81, 39.28, 31.92, 31.54, 31.23, 30.51, 30.38, 29.85, 29.66, 29.63, 29.52, 29.46, 29.40, 29.35, 28.09, 25.45, 22.86, 22.81, 22.69, 22.50, 22.48, 14.12, 13.98, 13.78. MALDI-TOF MS (m/z) calculated for $C_{90}H_{102}Cl_4N_8O_2S_5$: 1628.54539 $[M]^+$, found: 1628.55162.

Synthesis of IN-o2Cl: The synthetic route of IN-o2Cl is similar to that of IN-m2Cl except using 2,3-dichlorobenzoic acid instead of 2,4-dichlorobenzoic acid (yield 72.4%). ^{1}H NMR (400 MHz, $CDCl_3$) δ 7.89 (d, 1H), 7.82(d, 1H), 3.29 (s, 2H). ^{13}C NMR (101 MHz, $CDCl_3$) δ 194.74, 193.21, 143.08, 142.10, 139.83, 136.86, 130.17, 121.79, 45.29.

Synthesis of IC-o2Cl: The synthetic route of IC-o2Cl is similar to that of IC-m2Cl except using IN-o2Cl instead of IN-m2Cl (yield 77.1%). ^{1}H NMR (400 MHz, $CDCl_3$) δ 8.53 (d, 1H), 7.95 (d, 1H), 3.80 (s, 2H). ^{13}C NMR (101 MHz, $CDCl_3$) δ 190.77, 163.14, 142.40, 142.08, 136.88, 132.00, 124.32, 111.88, 111.65, 80.16, 43.94.

Synthesis of BTP-o-4Cl: The synthetic route of BTP-o-4Cl is similar to that of BTP-m-4Cl except using IC-o2Cl instead of IC-m2Cl (yield 96.2%). ^{1}H NMR (400 MHz, $CDCl_3$) δ 9.16 (s, 2H), 8.59 (d, 2H), 7.78 (d, 2H), 4.89-4.76 (m, 4H), 3.19 (t, 4H), 2.18-2.15 (m, 2H), 1.90-1.82 (m, 4H), 1.53-1.46 (m, 4H), 1.37-1.34 (m, 4H), 1.29-0.90 (m, 56H), 0.86 (t, 6H), 0.75-0.62 (m, 12H). ^{13}C NMR (101 MHz, $CDCl_3$) δ 185.06, 157.94, 154.07, 147.50, 145.30, 140.23, 140.01, 137.76, 136.60, 135.71, 135.30, 134.42, 133.54, 133.23, 131.19, 130.82, 123.69, 120.01, 115.30, 114.95, 113.60, 68.12, 55.85, 39.33, 31.92, 31.54, 31.21, 30.60, 30.41, 29.84, 29.66, 29.62, 29.52, 29.46, 29.39, 29.34, 28.16, 25.49, 22.89, 22.69, 22.51, 14.11, 13.97, 13.82, 13.77. MALDI-TOF MS (m/z) calculated for $C_{90}H_{102}Cl_4N_8O_2S_5$: 1628.54539 $[M]^+$, found: 1628.55397.

2. Characterization methods

^{1}H-NMR and ^{13}C-NMR spectra were obtained on a Bruker AVANCE III 400 MHz nuclear magnetic resonance (NMR) spectrometer. MALDI-TOF mass spectra were recorded using a JEOL JMS-S3000 Spiral-TOFMS (JEOL, Tokyo, Japan), Ions generated by irradiation with a 349-nm Nd:YLF laser were accelerated at 20 kV. The ions then passed along a spiral ion trajectory with a flight length of approximately 17 m. Cyclic voltammetry (CV) was done on a CHI600E electrochemical workstation with Pt disk, Pt plate, and standard calomel electrode (SCE) as working electrode, the counter electrode, and reference electrode, respectively, in a 0.1 mol·L^{-1} tetrabutylammonium hexafluorophosphate (Bu_4NPF_6) acetonitrile (CH_3CN) solution. The samples were drop cast on the glass carbon electrode to obtain a thin film for CV measurement. The CV curves were recorded versus the potential of SCE, which was calibrated by the ferrocene-ferrocenium (Fc/Fc^+) redox couple (4.8 eV below the vacuum level). Ultraviolet−visible light (UV-vis) absorption spectra were recorded on a Perkin Eimer Lambda 365 spectrophotometer. The photoluminescence

(PL) was determined by the FLS1000 fluorescence spectrometer. Data was collected at -173 k on a Bruker D8 venture single-crystal X-ray diffractometer, Mo-radiation. Absorption corrections were applied using the multiscan technique. The structures were solved by the direct method and refined by the full-matrix least-squares method on F^2 using the SHELXL-97 software.

3. Device fabrication and testing

ITO-coated glass substrates were cleaned with detergent water, deionized water, acetone, and isopropyl alcohol in an ultrasonic bath sequentially for 30 min, and further treated with UV exposure for 30 min in a UV-ozone chamber. Hole transport layer interface condition: 1) PEDOT:PSS was diluted with the same volume of water, a thin layer of PEDOT:PSS (~20 nm) (Clevios AL4083) was spin-coated onto the UV-treated substrates, the PEDOT-coated substrates were subsequently annealed on a hot plate at 150 °C for 10 min, and the substrates were then transferred into the glovebox for active layer deposition; 2) About 50 uL 2Br-2Pac (0.3 mg mL^{-1} in isopropyl alcohol) was spin-coated at 2000 rpm for 20s, the 2Br-2Pac -coated substrates were subsequently annealed on a hot plate at 50 °C for 10 min, and the substrates were then transferred into the glovebox for active layer deposition. The optimized concentration was 16 mg·mL^{-1} chloroform solution with a D:A ratio of 1:1.3 (w/w). Note: The as-prepared solutions were stirred for 2h at room temperature before being spin coat on the PEDOT:PSS or 2Br-2Pac substrates. The active layers were spin-coated at an optimized speed of 2000 rpm ~ 2500 rpm for a time period of the 30s, resulting in films of 140 to 150 nm in thickness. The active layers were then Solvent annealed (CS_2,40s) and thermal annealed (TA) for 5 min at 90 °C. The active layer thickness was around 140~150 nm. Then, Phen-NaDPO as the electron transporting layer was spin-coated on the active layer by 2000 rpm for 20s from isopropyl alcohol solution. Finally, Finally, the samples were placed in a thermal evaporator for evaporation of a 90 nm-thick layer of Silver (Ag) evaporated at 2 Å s^{-1}; pressure of less than $2x10^{-6}$ Torr. Following electrode deposition, samples underwent *J−V* testing.

The current density-voltage (*J-V*) curves of devices were recorded under AM 1.5G illumination provided by an AAA class solar simulator (Enli Technology Co., Ltd. SS-X50R) calibrated by a silicon reference cell with KG2 filter (supported from Enli Tech. Co., Ltd., Taiwan). The external quantum efficiency (EQE) was measured by a certified incident photon to electron conversion (IPCE) equipment (QE-R) from Enli Technology Co., Lt. The light intensity at each wavelength was calibrated using a standard monocrystalline Si photovoltaic cell. The IQE of optimized blend films were calculated from the following equation:

$$IQE = \frac{EQE}{(1 - R\% - Parasitic\ absorption)}$$

The parasitic absorption was obtained from the transfer matrix model[1].

SCLC Mobility Measurements: Fitting the hole/electron-only diode dark current to the space charge limited current (SCLC) model can obtain the hole and electron mobility of the photosensitive active layer. The electron-only device structure was ITO/ZnO/ Phen-NaDPO / Active layer / Phen-NaDPO /Ag, and the hole-only device structure was ITO/ MoO_3/ Active layer /MoO_3/Ag. Using the following equation to estimate the electric-field dependent SCLC mobility:[2]

$$J(V) = \frac{9}{8}\varepsilon_0\varepsilon_r\mu_0 exp\left(0.89\beta\sqrt{\frac{V - V_{bi}}{L}}\right)\frac{(V - V_{bi})^2}{L^3}$$

For the hole-only device structure, V_{bi} = 0 V (flat band pattern formed by MoO_3-MoO_3); For the electron-only device structure, V_{bi} = 0.5 V was used following the protocol reported.

Transient photovoltage (TPV) and transient photocurrent (TPC)： For TPV, the measurement was conducted under 1 sun conditions by illuminating the device with a white light-emitting diode, and the device is set to the open-circuit condition. For TPC, the device is set to the short-circuit condition in dark. The output signal was collected by keysight oscilloscope. The photovoltage decay kinetics of all devices follow a mono-exponential decay: $\delta V = Aexp(-t/\tau)$ where t is the time, and τ is the charge carrier lifetime.

Grazing Incidence Wide-angle X-ray Scattering: Silicon substrates for the GIWAXS test were sonicated for 15 min each in successive baths of detergent, DI water, acetone, and isopropanol. The substrates were then dried with pressurized nitrogen before being exposed to the UV−ozone plasma for 15 min. The BHJ layers were prepared following methods described in the Section of Device Fabrication. All samples for GIWAXS were radiated at 10 keV X-ray with an incident angle of 0.13°, SDD (mm) is 205.811, Pixel size (mm) is 0.075, Pixel distance of Standard (Peak position) is 114, q of standard ($Å^{-1}$) is 0.2104.

Atomic Force Microscopy (AFM) Imaging: Topographic images of the films were obtained from a Bruker atomic force microscopy (AFM) with the type of dimension edge with Scan Asyst™ in the tapping mode using an etched silicon cantilever at a nominal load of ~2nN, the

scanning rate for a 2 μm×2 μm image size was 0.9 Hz and 5 μm×5 μm image size was 1.0 Hz. Films were spun-cast on 2Br-2Pac-coated ITO glass substrates.

Transmission Electron Microscopy (TEM) Characterization: Transmission electron microscope (TEM) studies were conducted with a FEI Talos F200S electron microscopy to investigate the phase distribution of the active layer. Films were spun-cast on 2Br-2Pac-coated glass substrates. The BTP-m-4Cl, BTP-o-4Cl, and BHJ films were floated off the substrates in isopropyl alcohol and collected on lacey without carbon coated TEM grids (Electron Microscopy Sciences).

4. Supplemental figures

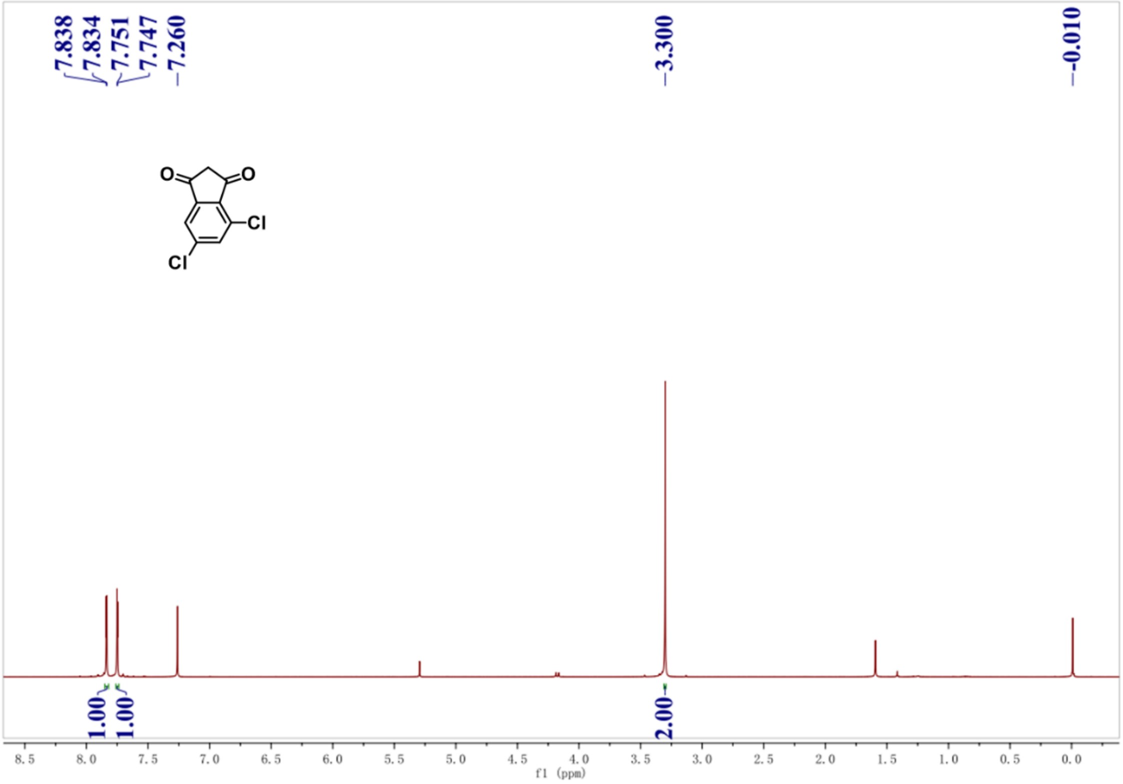

Figure S2. ^{1}H-NMR spectrum of IN-m2Cl in $CDCl_3$.

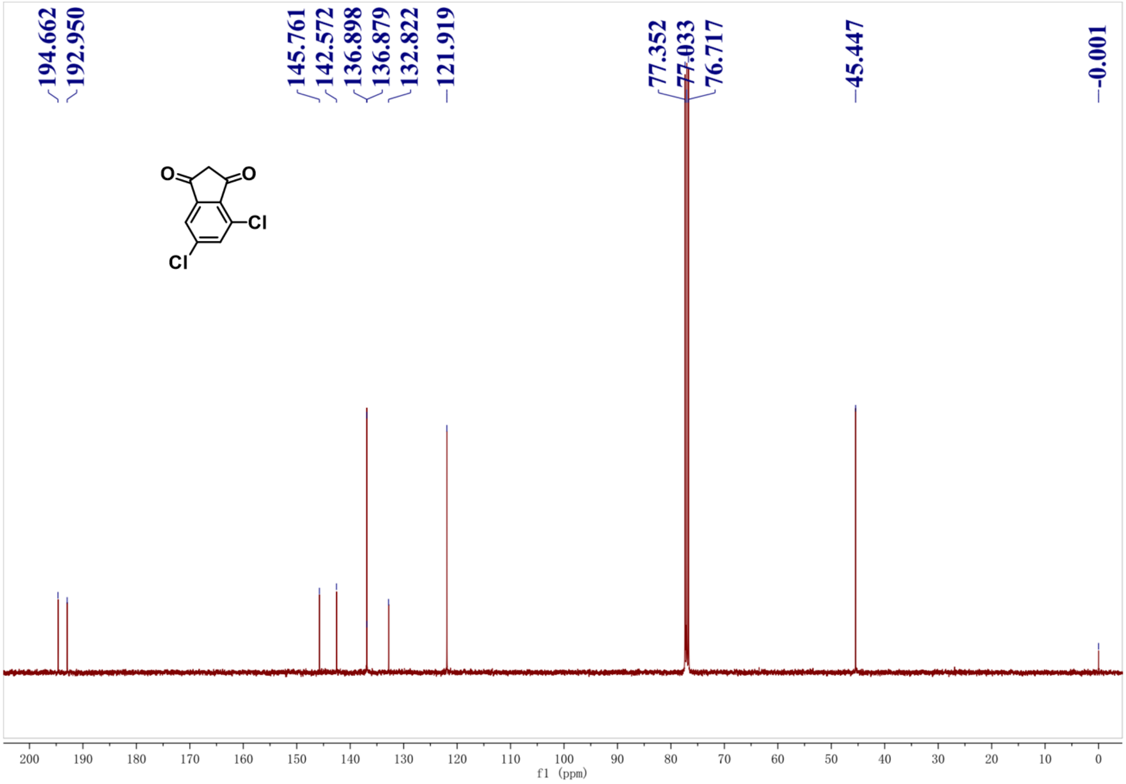

Figure S3. ^{13}C-NMR spectrum of IN-m2Cl in $CDCl_3$.

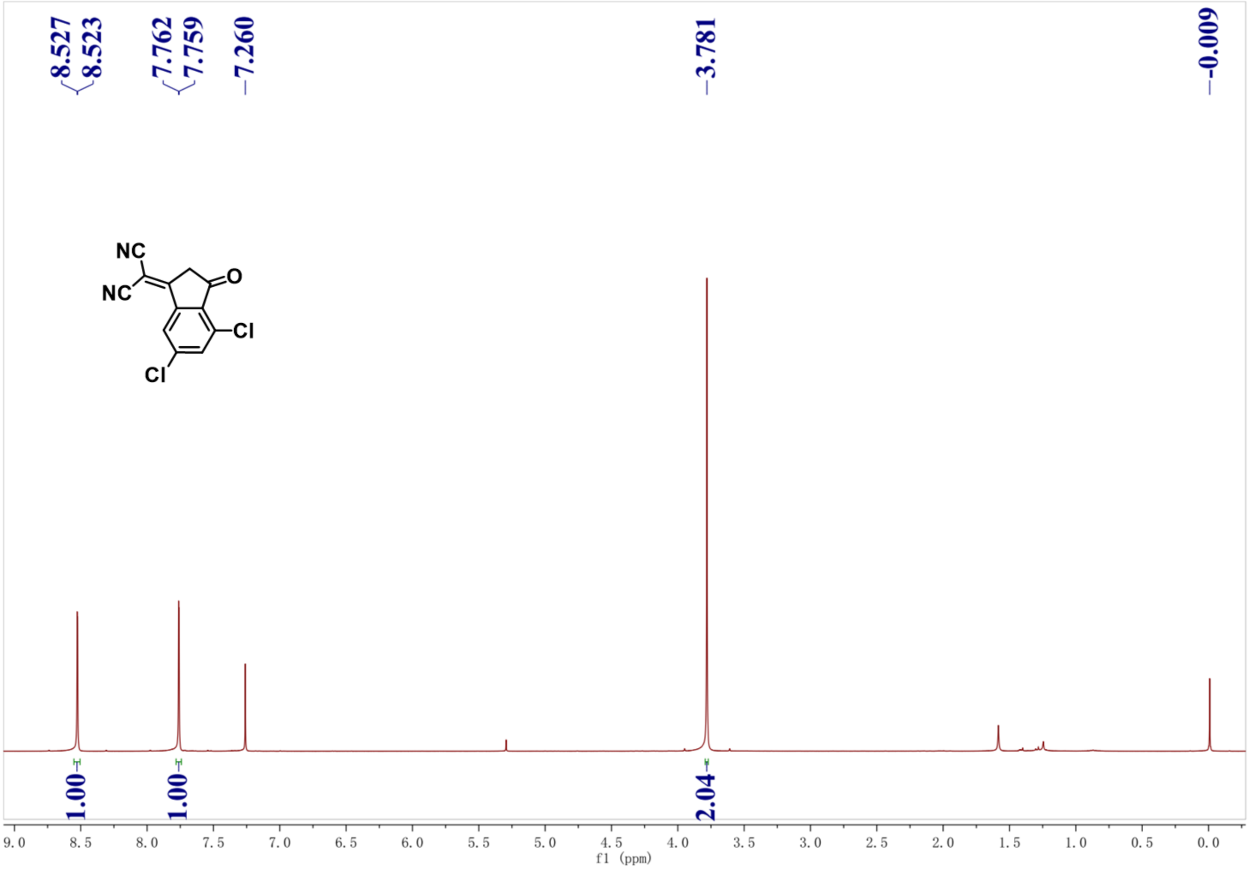

Figure S4. ^{1}H-NMR spectrum of IC-m2Cl in $CDCl_3$.

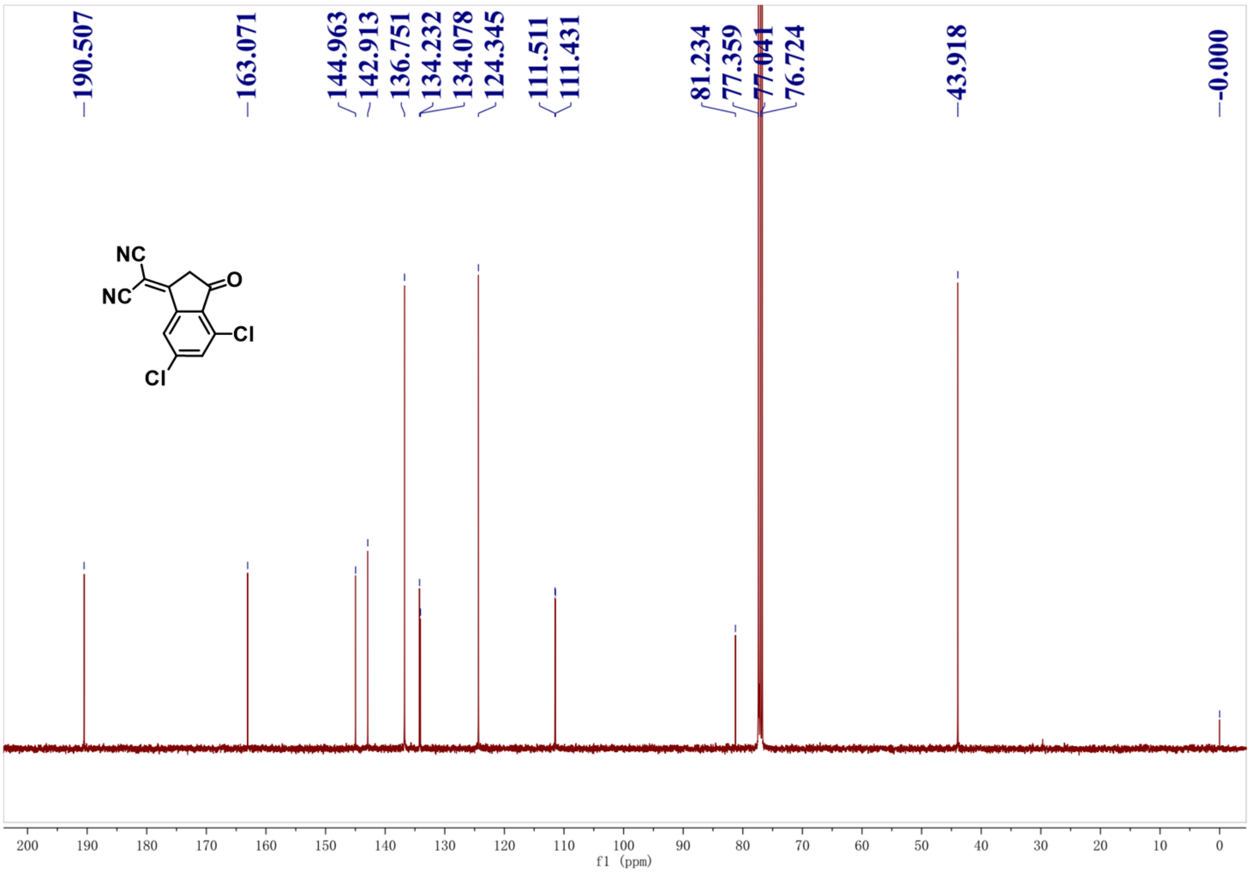

Figure S5. ^{13}C-NMR spectrum of IC-m2Cl in $CDCl_3$.

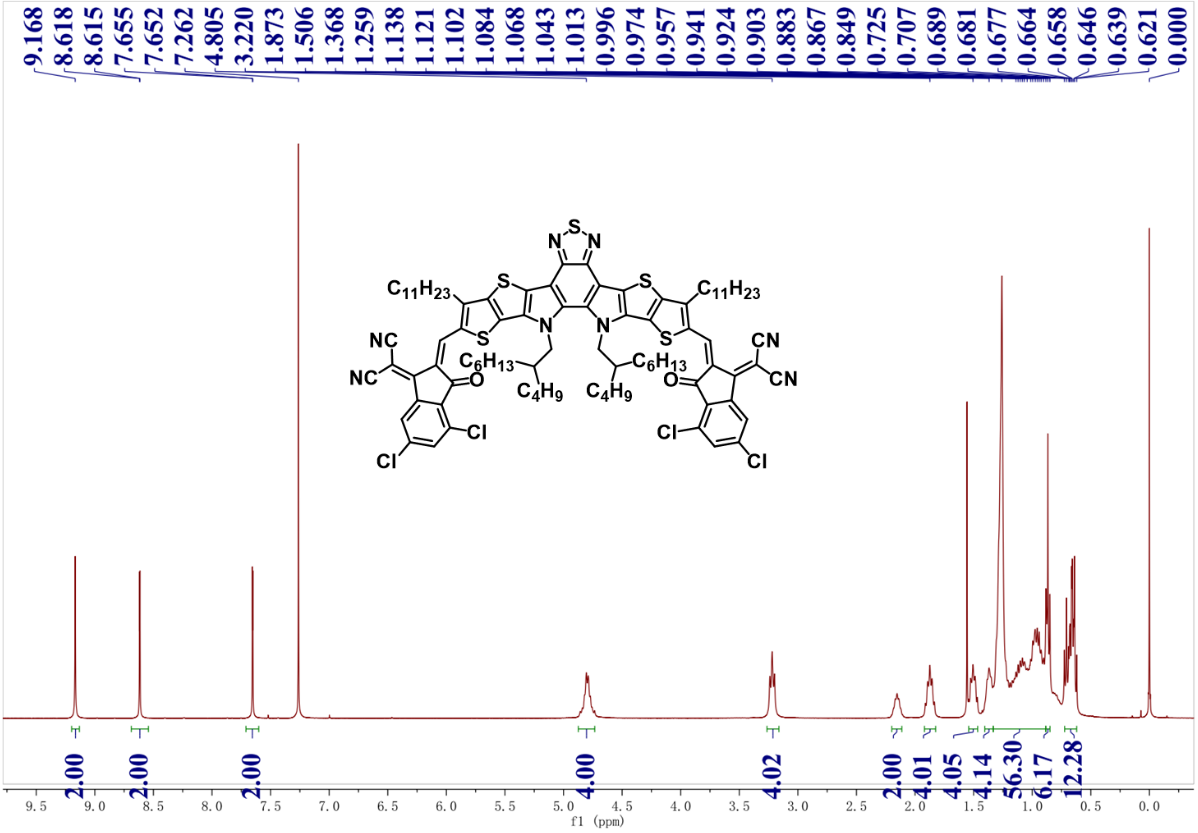

Figure S6. ^{1}H-NMR spectrum of BTP-m-4Cl in $CDCl_3$.

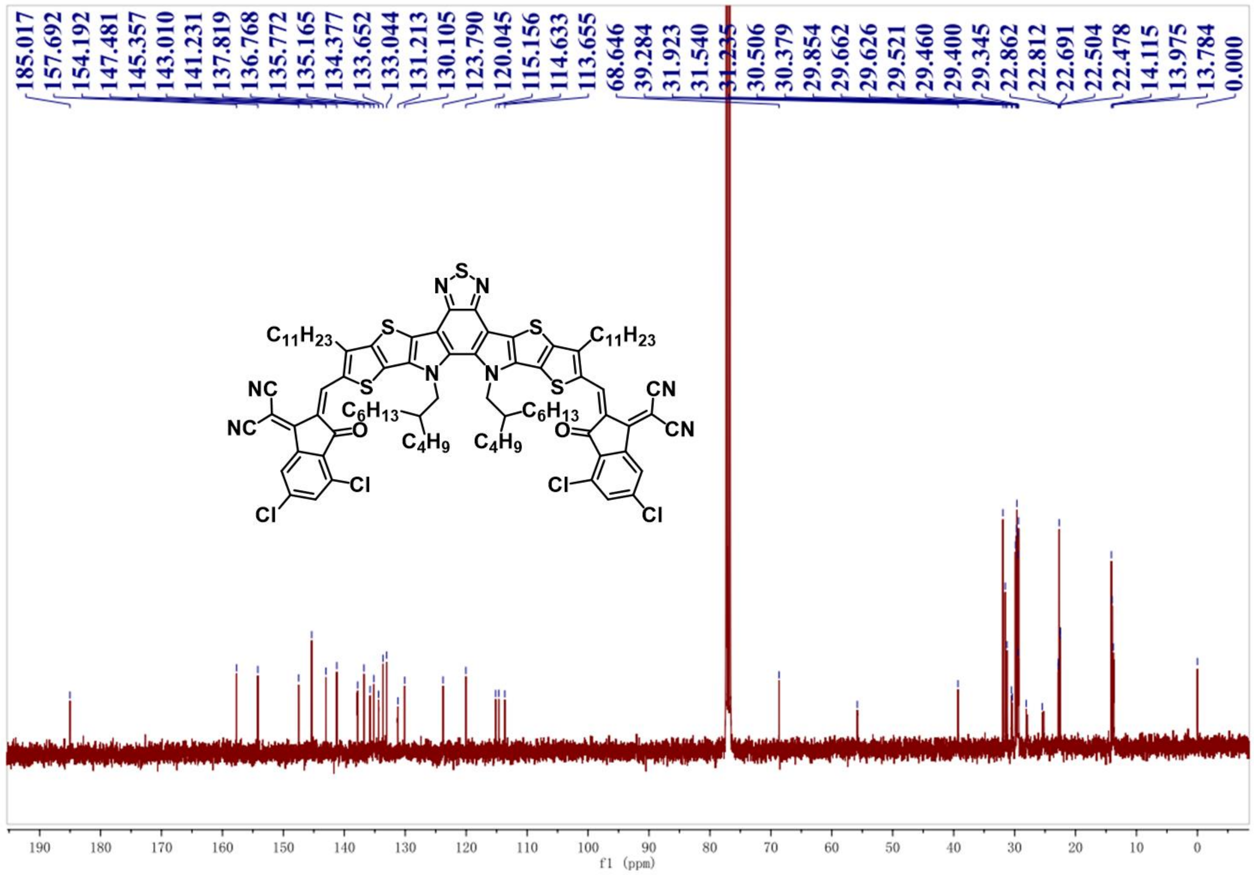

Figure S7. ^{13}C-NMR spectrum of BTP-m-4Cl in $CDCl_3$.

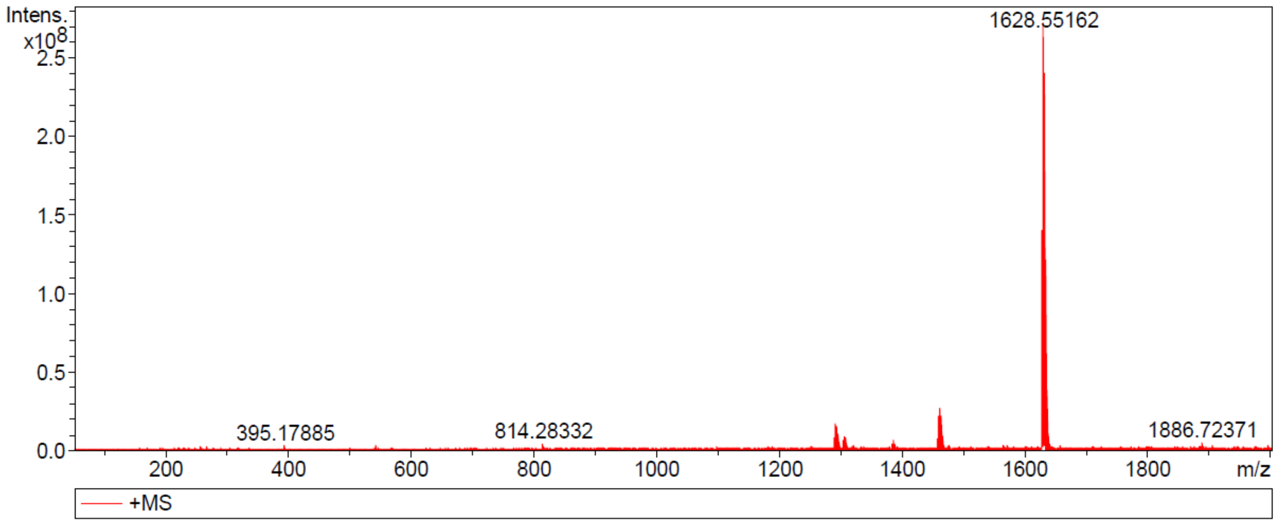

Figure S8. MALDI-TOF spectrum of BTP-m-4Cl.

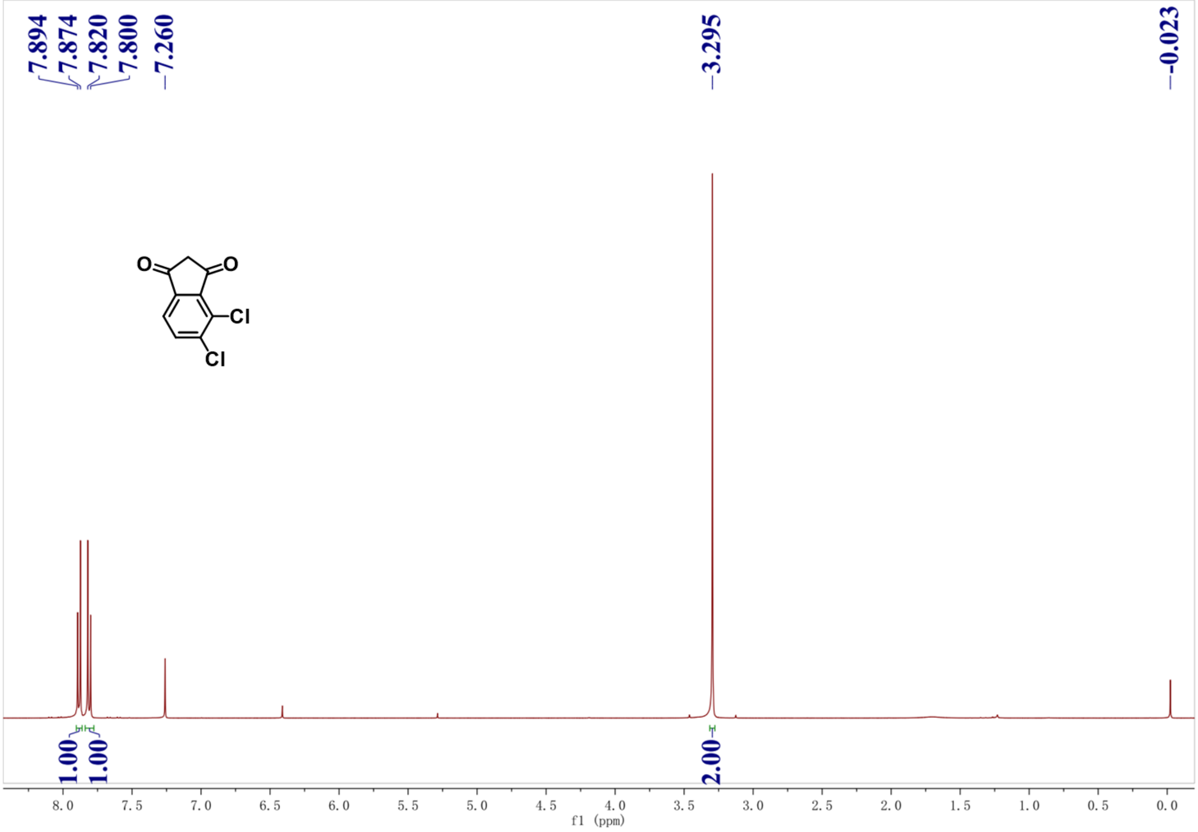

Figure S9. ^{1}H-NMR spectrum of IN-o2Cl in $CDCl_3$.

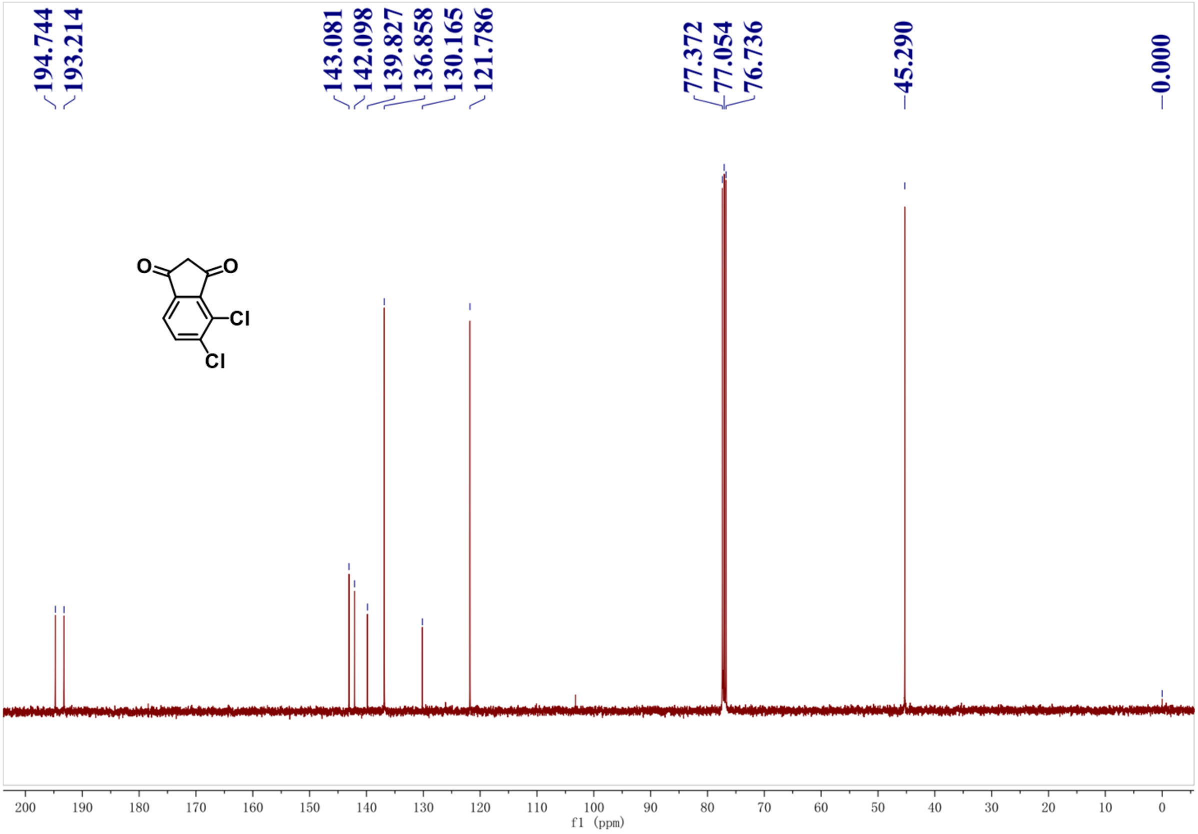

Figure S10. ^{13}C-NMR spectrum of IN-o2Cl in $CDCl_3$.

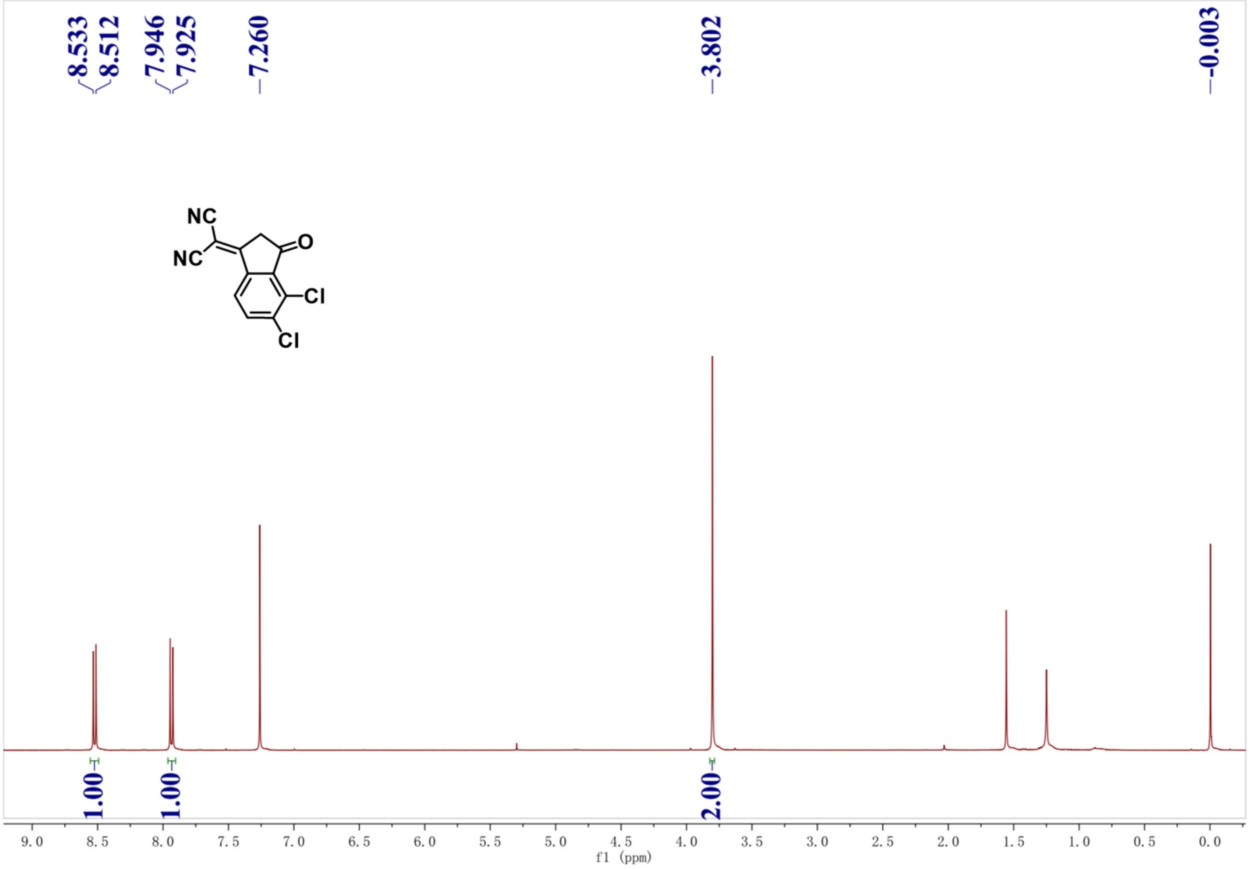

Figure S11. ^{1}H-NMR spectrum of IC-o2Cl in $CDCl_3$.

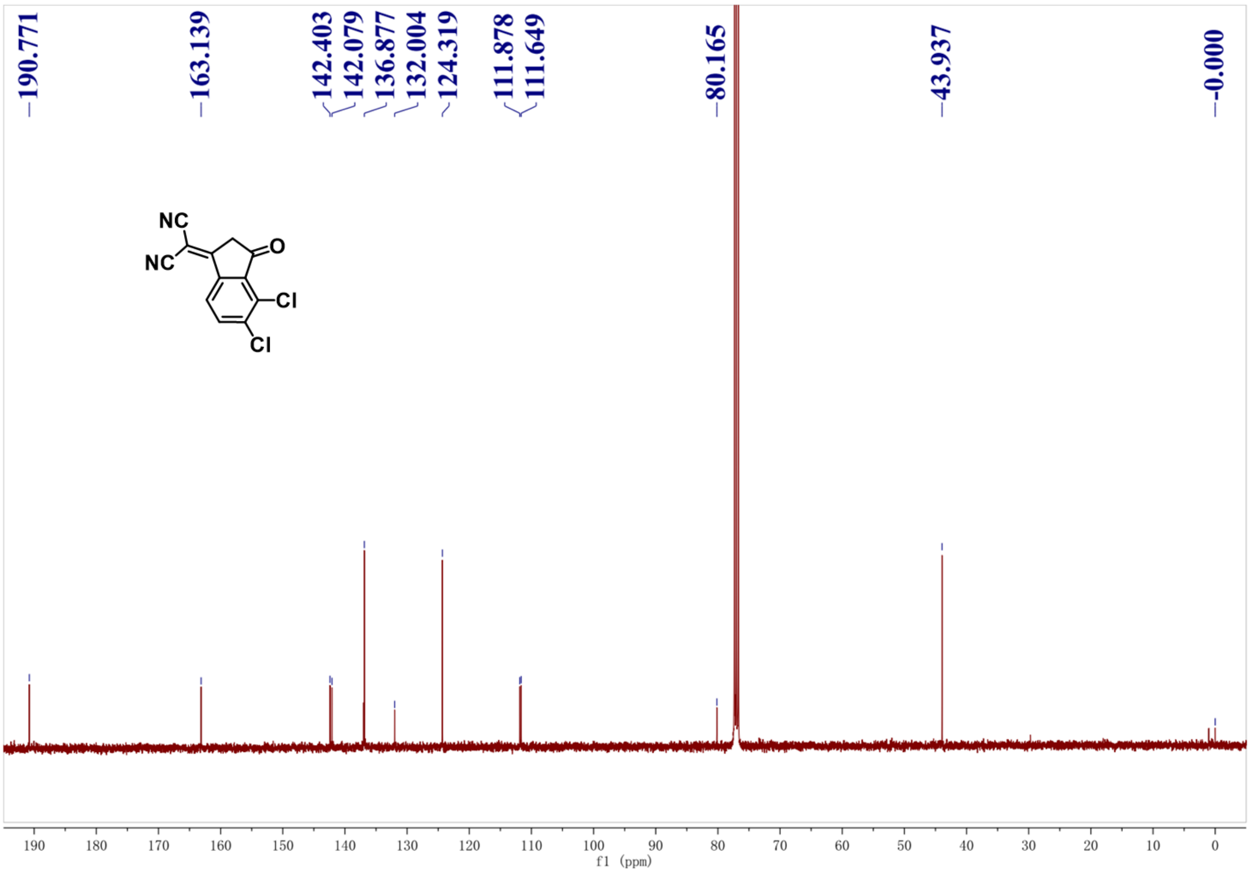

Figure S12. ^{13}C-NMR spectrum of IC-o2Cl in $CDCl_3$.

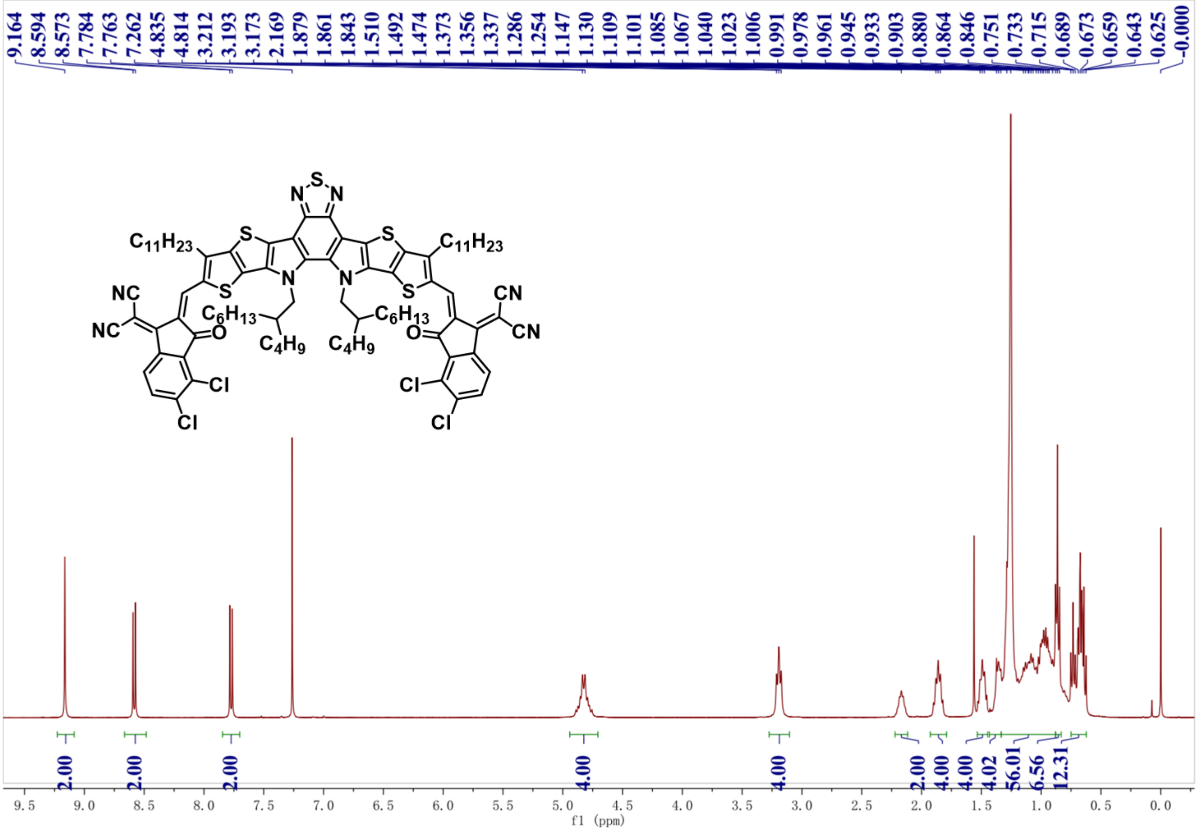

Figure S13. ^{1}H-NMR spectrum of BTP-o-4Cl in $CDCl_3$.

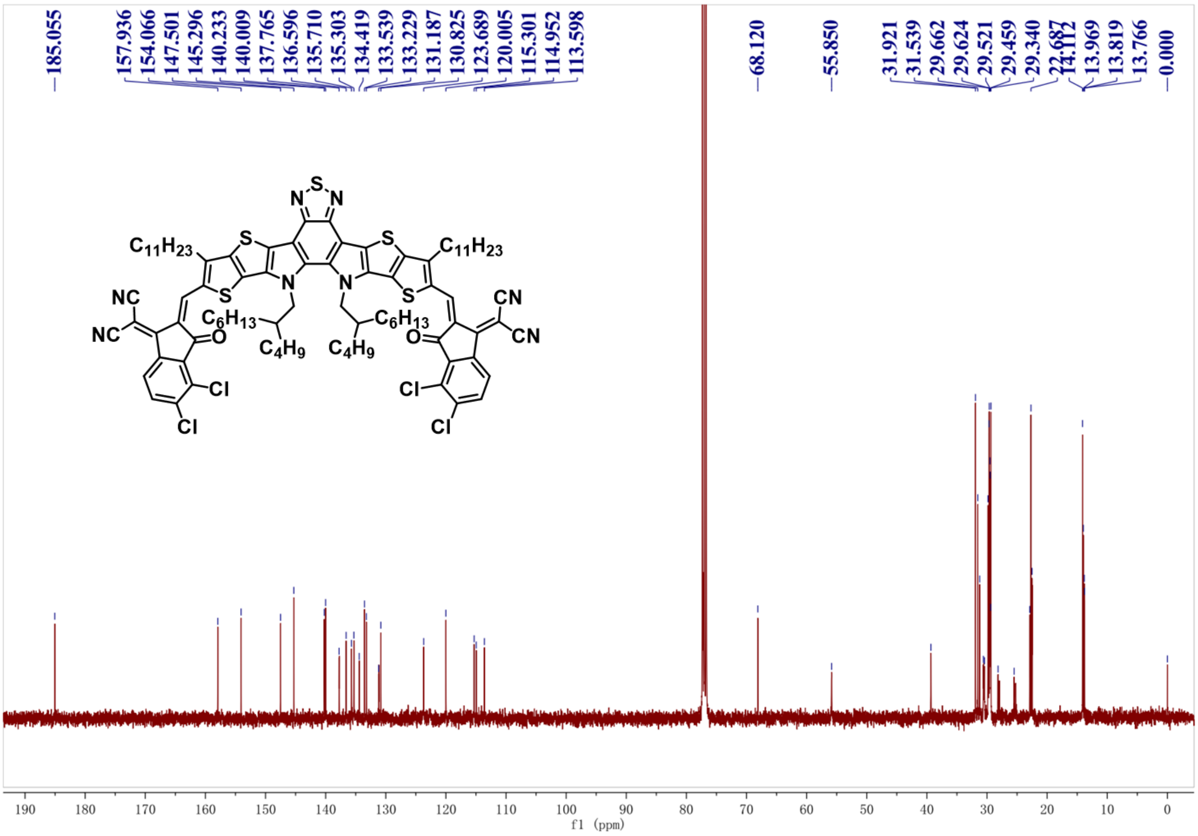

Figure S14. ^{13}C-NMR spectrum of BTP-o-4Cl in $CDCl_3$

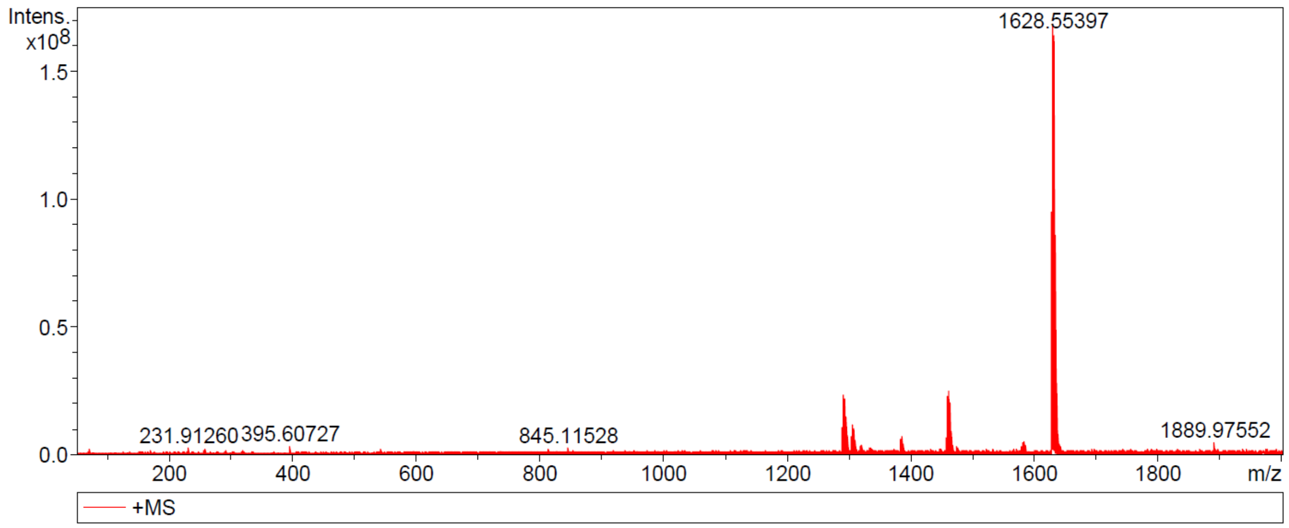

Figure S15. MALDI-TOF spectrum of BTP-o-4Cl.

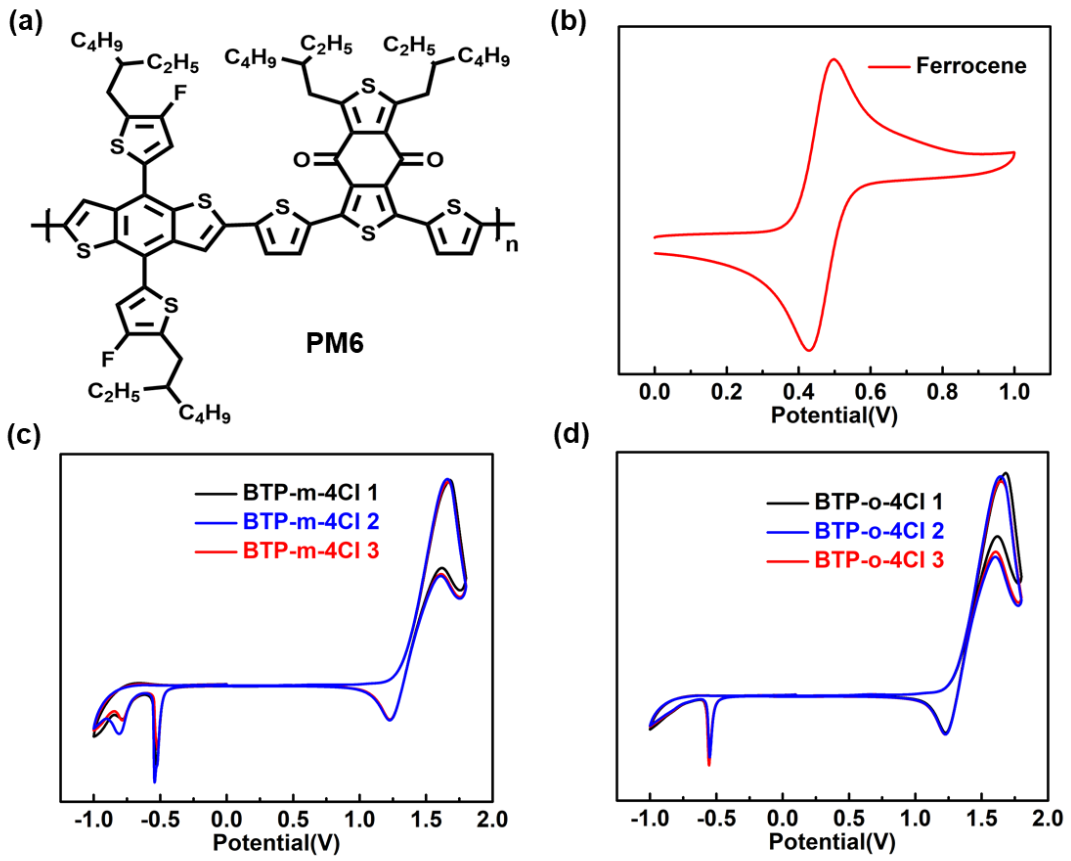

Figure S16. (a)Molecular structures of PM6. Cyclic voltammograms of (b) Ferrocence, (c) BTP-m-4Cl, and (d) BTP-o-4Cl films measured in 0.1 M Bu_4NPF_6 solution at the scan rate of 50 mV/s.

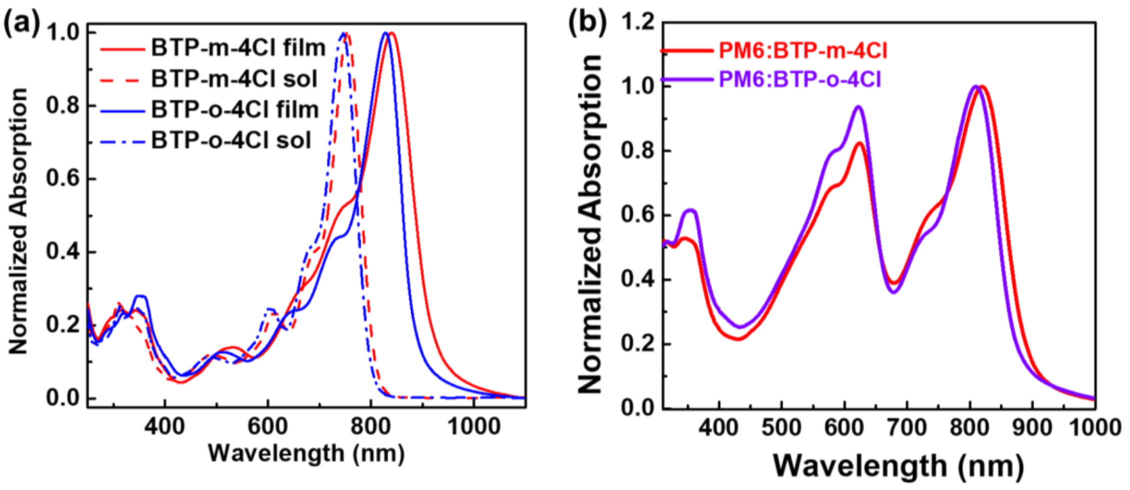

Figure S17. UV-vis absorption spectra of BTP-o-4Cl, BTP-o-4Cl, and blended films.

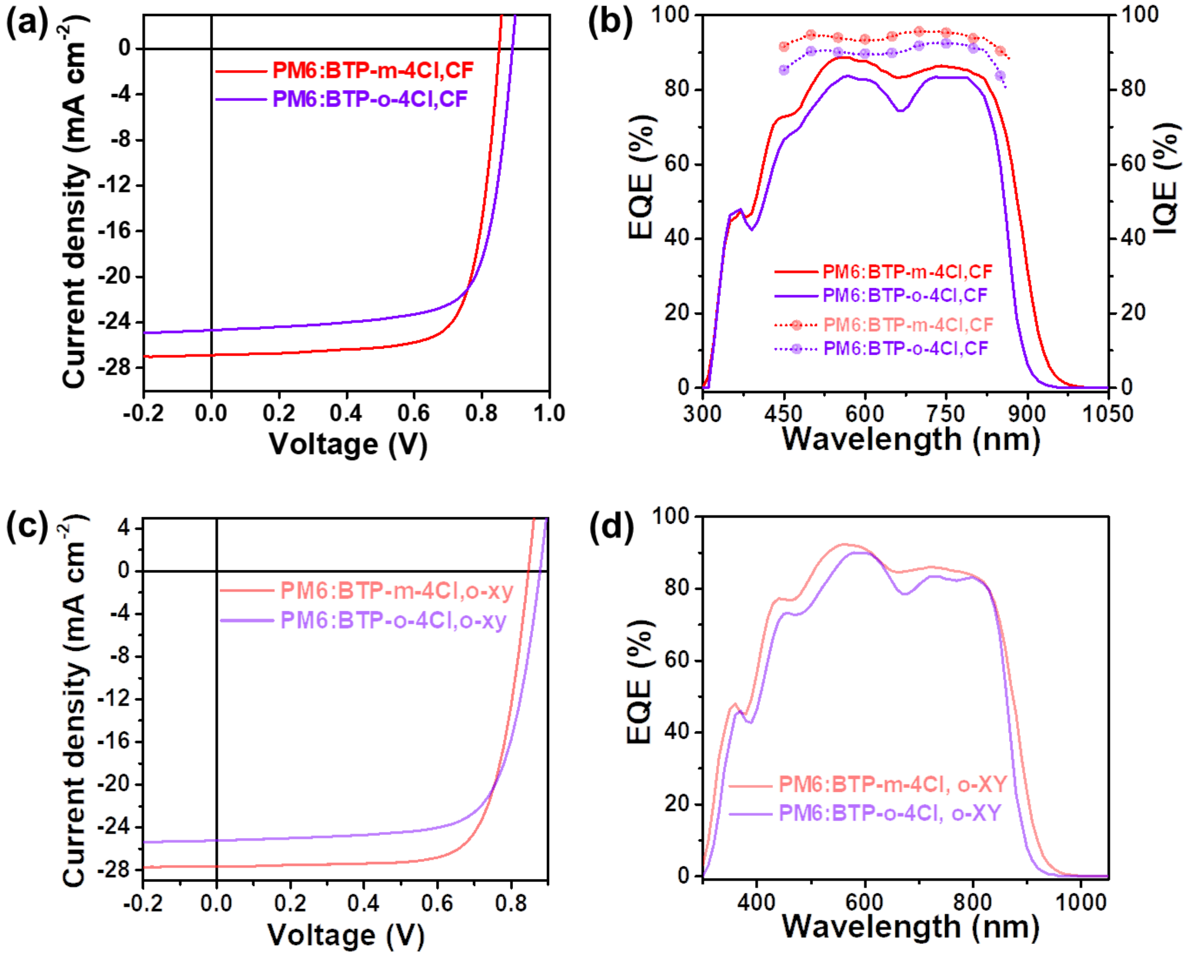

Figure S18. (a) J-V and, (b) EQE and IQE of the optimal devices in $CHCl_3$ solvent system. (c) J-V and (d) EQE of the optimal devices in o-xylene solvent system.

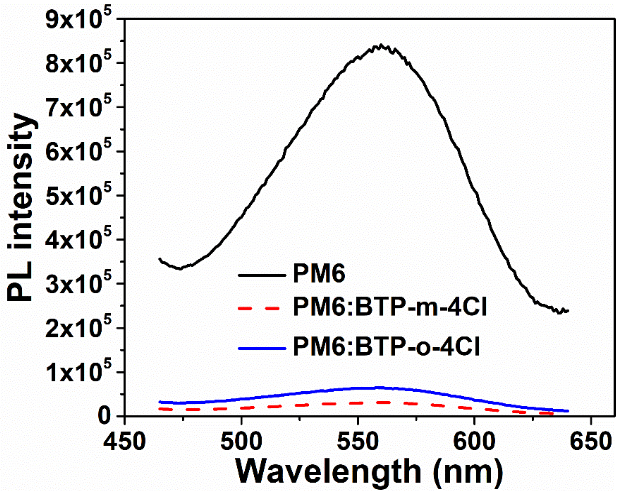

Figure S19. Photoluminescence (PL) spectra based on PM6, PM6:BTP-m-4Cl, and PM6:BTP-o-4Cl thin films at the wavelength of 560 nm.

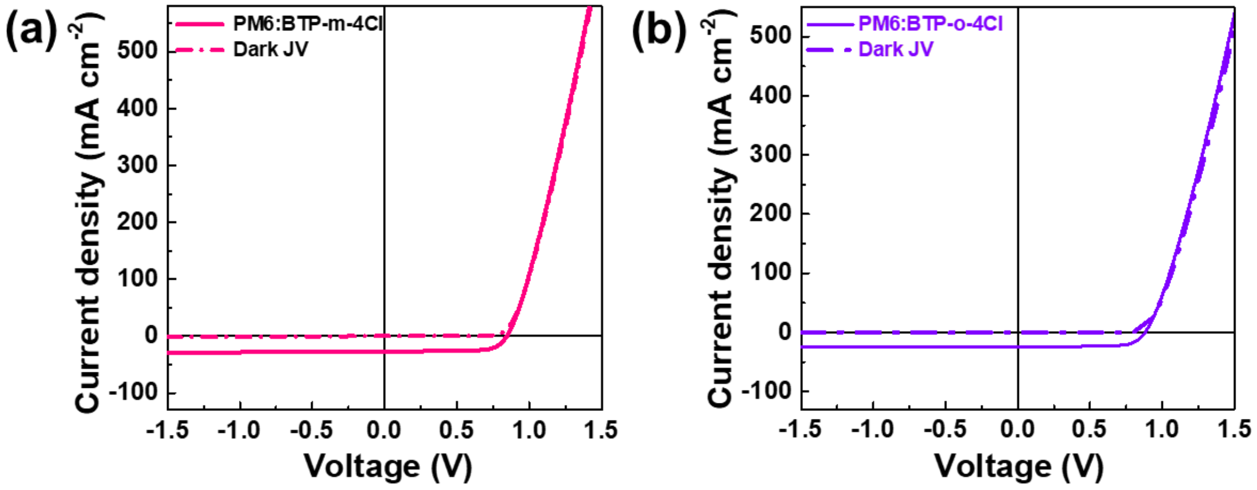

Figure S20. The original *J-V* curves and dark *J-V* curves of (a) PM6: BTP-m-4Cl, and (b) PM6: BTP-o-4Cl were used to calculate exciton dissociation efficiency.

Table S1. The corresponding parameters of exciton dissociation efficiency ($\eta_{diss} = J_{SC} / J_{\mathrm{ph},\, sat}$) and charge collection efficiency ($\eta_{coll} = J_{max\ power} / J_{ph,\, sat}$) of PM6:BTP-m-4Cl and PM6:BTP-o-4Cl based devices.

Parameter	PM6:BTP-m-4Cl	PM6:BTP-o-4Cl
$J_{\mathrm{ph},\, sat}$ (mA/cm^2)	27.73	25.30
J_{SC} (mA/cm^2)	26.93	24.46
$\eta_{diss} = J_{SC} / J_{\mathrm{ph},\, sat}$ (%)	97.09	96.67
J_{max} (mA/cm^2)	23.75	20.96
$\eta_{coll} = J_{max\ power} / J_{\mathrm{ph},\, sat}$ (%)	85.65	82.82

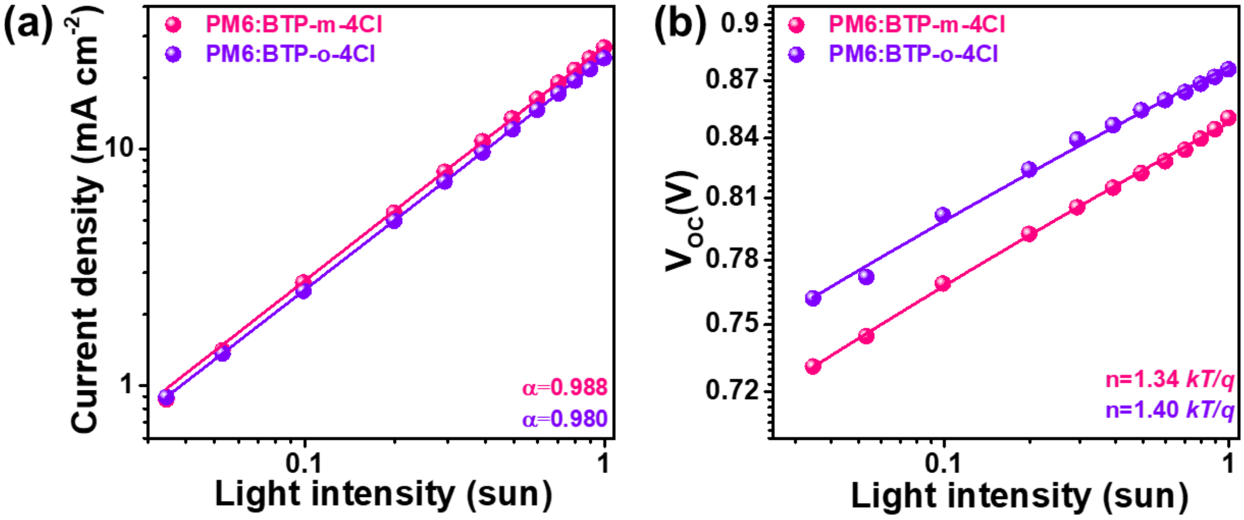

Figure S21. The fitted results of (a) J_{SC} and (b) V_{OC} under various incident light intensities. Fitted equations: $J_{SC} \propto I^{\alpha}$ and $V_{oc} \propto n(kT/q)ln(I)$.

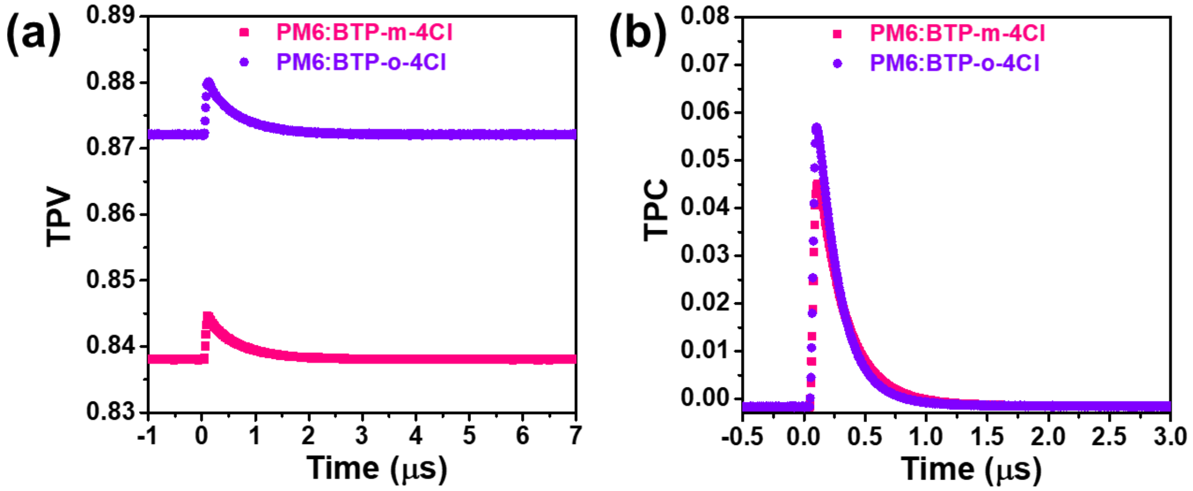

Figure S22. (a) Transient photovoltage (TPV) and (b) transient photocurrent (TPC) measurements of PM6:BTP-m-4Cl and PM6:BTP-o-4Cl based devices.

Table S2. Summary of mobilities for PM6:BTP-m-4Cl and PM6:BTP-o-4Cl based OSC devices.

Active layer	μ_h [$\times 10^{-4}$ cm^2 V^{-1} s^{-1}]	μ_e [$\times 10^{-4}$ cm^2 V^{-1} s^{-1}]	μ_e / μ_h
PM6: BTP-m-4Cl	4.35±0.98	6.37±0.21	1.46
PM6: BTP-o-4Cl	3.76±0.69	5.99±0.49	1.60

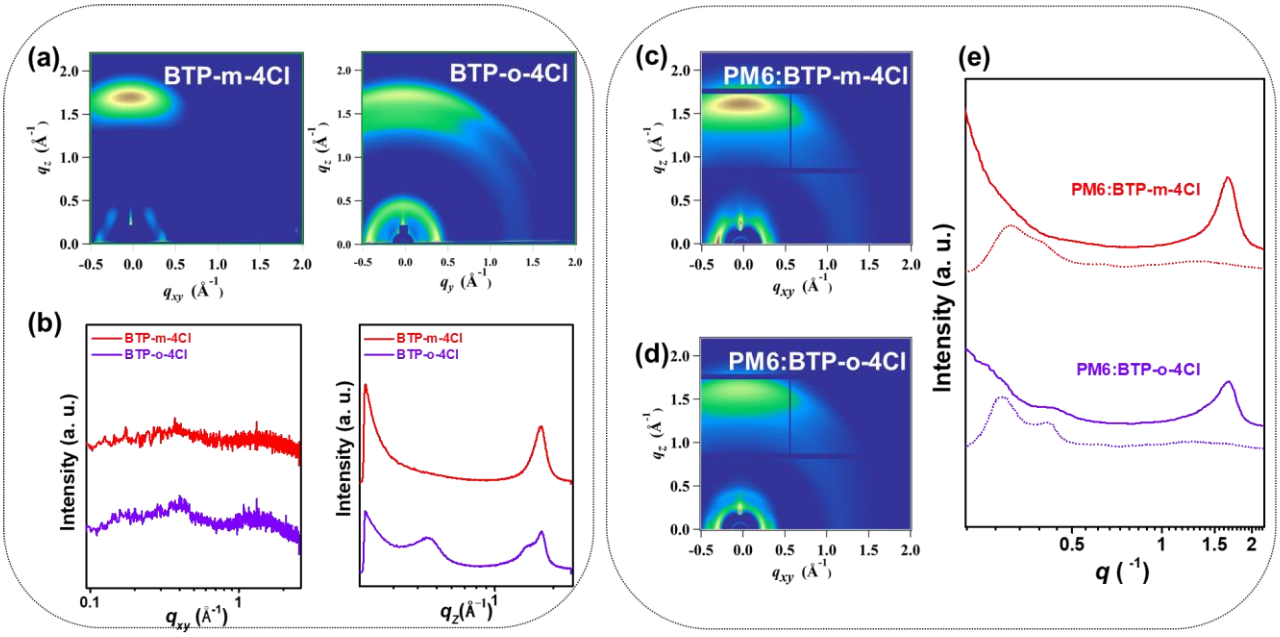

Figure S23. (a) GIWAXS patterns of pure films. (b) In-plane and out-of-plane line-cut profiles of the corresponding GIWAXS intensity profiles. (c, d) GIWAXS patterns of blended films. (e) In-plane (dotted lines) and out-of-plane (solid lines) line-cut profiles of the corresponding GIWAXS intensity profiles.

Table S3. Detailed GIWAXS peak information in-plane (IP) and out-of-plane (OOP) of BTP-m-4Cl, BTP-o-4Cl, PM6:BTP-m-4Cl and PM6:BTP-o-4Cl thin film.

Component	Peak	Peak location (Å^{-1})	d-spacing (Å)	FWHM (Å^{-1})	Crystal coherence length(nm)
BTP-m-4Cl	**(010) OOP**	**1.720**	**3.653**	**0.023**	**24.88**
	(100) IP	0.376	16.711	0.040	14.14
BTP-o-4Cl	(100) OOP	0.427	14.715	0.025	22.66
	**(010) OOP**	**1.627**	**3.862**	**0.053**	**10.78**
	(100) IP	0.367	17.120	0.034	16.64
PM6:BTP-m-4Cl	(100) IP	0.304	20.668	0.068	8.31
	(100) OOP	0.195	32.221	0.062	9.12
	**(010) OOP**	**1.660**	**3.785**	**0.260**	**2.20**
PM6:BTP-o-4Cl	(100) IP	0.291	21.592	0.067	8.44
	(100) OOP	0.186	33.781	0.042	13.46
	**(010) OOP**	**1.653**	**3.801**	**0.287**	**1.99**

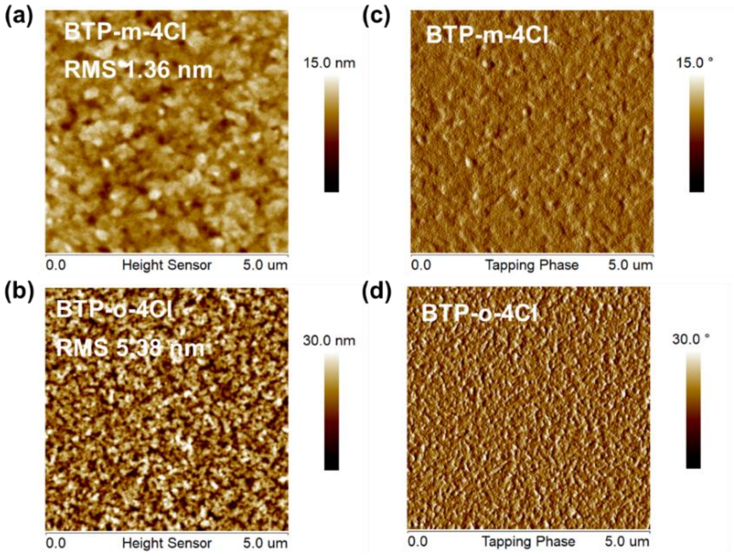

Figure S24. (a, b) AFM height image and (c, d) phase image of BTP-m-4Cl and BTP-o-4Cl pure films.

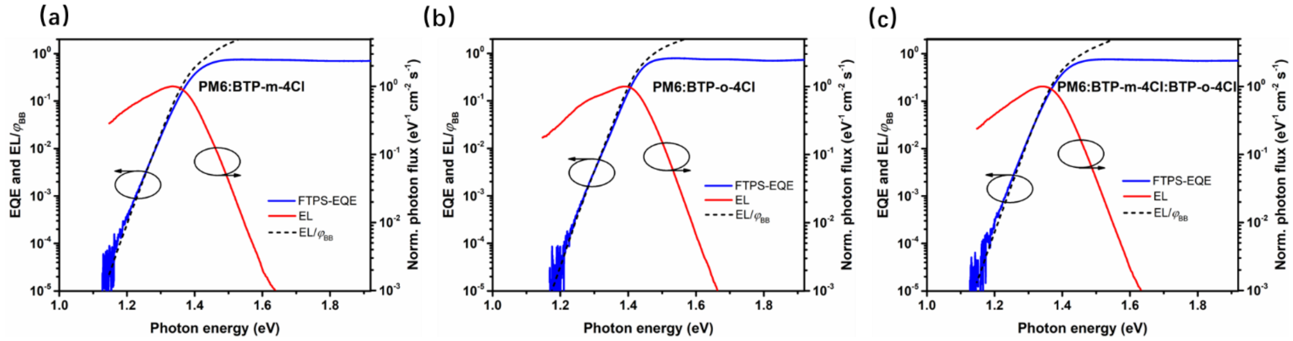

Figure S25. EL spectra and normalized s-EQE values of (a) PM6:BTP-m-4Cl, (b) PM6:BTP-o-4Cl and (d) PM6:BTP-m-4Cl :BTP-o-4Cl.

Table S4. Detailed energy losses of PM6:BTP-m-4Cl, PM6:BTP-o-4Cl and PM6:BTP-m-4Cl:BTP-o-4Cl based devices.

Device	E_g^{PV} (eV)	V_{OC}^{Cal} [V]	qV_{OC}^{SQ} [eV]	qV_{OC}^{rad} [eV]	[a]ΔE1 (eV)	[b]ΔE2 (eV)	[c]ΔE3 (eV)	E_{loss} (eV)	EQE_{EL} ($\times 10^{-2}$%)
PM6:BTP-m-4Cl	1.40	0.844	1.138	1.080	0.262	0.058	0.236	0.556	1.08
PM6:BTP-o-4Cl	1.43	0.882	1.167	1.122	0.263	0.045	0.240	0.548	0.91
PM6:BTP-m-4Cl:BTP-o-4Cl	1.40	0.858	1.138	1.085	0.262	0.053	0.227	0.542	1.53

[a] $\Delta E_1 = E_g - qV_{OC}^{SQ}$; ΔE_1 is attributed to radiative recombination from the absorption above the bandgap. Where V_{OC}^{SQ} is the maximum voltage by the Shockley–Queisser limit; q is the elementary charge. [b] $\Delta E_2 = qV_{OC}^{SQ} - qV_{OC}^{rad}$; ΔE_2 is attributed to additional radiative recombination from the absorption below the bandgap. Where V_{OC}^{rad} is the open-circuit voltage when there is only radiative recombination. [c] $\Delta E_3 = qV_{OC}^{non-rad} = -kTln(EQE_{EL})$; ΔE_3 is nonradiative recombination loss.

1

中 国 计 量 科 学 研 究 院

测 试 报 告

Test Report

报告编号 GXgf2021-12550
Report No

客户名称 Client	中国科学研究院重庆绿色智能技术研究院 Chongqing Institute of Green and Intelligent Technology , CAS
样品名称 Sample	有机太阳能电池 Organic Solar Cells
型号/规格 Type/Model	有机太阳能电池 Organic Solar Cells
出厂编号 Serial No.	L34-M1-F-2
生产厂商 Manufacturer	中国科学研究院重庆绿色智能技术研究院 Chongqing Institute of Green and Intelligent Technology , CAS
客户地址 Client Address	重庆市北碚区方正大道 266 号, No.266 Fangzheng Avenue, Beibei District,Chongqing
测试日期 Date of Test	2021-07-29

批准人:
Approved by

中国计量科学研究院 测试专用章

地址：中国 北京 北三环东路 18 号
Address：No.18 Bei San Huan Dong Lu,Beijing,P.R.China
邮编：100029
Post Code
电话：+86-10-64525569/74
Tel
传真：+86-10-64271948
Fax
网址：http://www.nim.ac.cn
Website
电子邮箱：kehufuwu@nim.ac.cn
Email

2019-cs-R0520

2

中 国 计 量 科 学 研 究 院

报告编号 GXgf2021-12550
Report No.

中国计量科学研究院（NIM）是国家最高的计量科学研究中心和国家级法定计量技术机构。1999 年授权签署了国际计量委员会（CIPM）《国家计量基(标)准和国家计量院签发的校准与测量证书互认协议》(CIPM MRA)。The National Institute of Metrology (NIM) is China's national metrology institute (NMI) and a state-level legal metrology institute. NIM is China' s signatory to the Mutual Recognition of National Measurement Standards and of Calibration and Measurement Certificates Issued by National Metrology Institutes (CIPM MRA) which is arranged by the International Committee of Weights and Measures (CIPM).

质量管理体系符合 ISO/IEC17025 标准，通过中国合格评定国家认可委员会（CNAS）和亚太计量规划组织（APMP）联合评审的校准和测量能力（CMCs）在国际计量局（BIPM）关键比对数据库中公布。NIM's quality management system meets requirements of the ISO/IEC 17025. Its Calibration and Measurement Capabilities (CMCs) that are peer reviewed both by China National Accreditation Service for Conformity Assessment (CNAS) and the Asia Pacific Metrology Programme (APMP) are published in the International Bureau of Weights and Measures (BIPM) Key Comparison Database (KCDB).

2020 年，NIM 和 CNAS 就认可领域的技术评价活动签署了谅解备忘录，承认 NIM 的计量支撑作用和出具的校准/检测结果的溯源效力。NIM and CNAS signed a Memorandum of Understanding (MOU) for Recognition of Technical Assessment in Laboratory Accreditation Field in 2020, in which CNAS recognizing the technical supporting role of NIM in laboratory accreditation and the traceability of NIM's calibration / test results.

测试所依据/参考的技术文件（代号、名称）Reference documents (Code,Name)

参照 JJF 1622-2017 太阳电池校准规范：光电性能

测试环境条件及地点 Testing place and environment:
温 度 Temperature: （23±1）℃ 地 和-13-110
湿 度 Humidity: （67±2）%RH 其 它 Others: /

测试使用的计量基（标）准装置（含标准物质)/主要仪器
Reference Standards（Including Reference Material）/Instruments used

名 称 Name	测量范围 Measurement Range	不确定度/准确度等级 Uncertainty/ Accuracy	报告编号 Certificate No.	证书有效期至 Due Date (YYYY-MM-DD)
标准太阳电池 Reference solar cell	I_{sc}:(0.1-200) mA	1.3%(k=2)	GXgf2021-10109	2022-01-13
小面积稳态太阳模拟器 Steady solar simulator	(300-1100) nm	AAA 级	GXgf2021-11814	2022-06-24
源表 Sourcemeter	DC-V：10mV-10V DC-I: 100μA-1A	（5×10^{-4}-10×10^{-5}）（k=2）（10×10^{-5}-40×10^{-5}）(k=2)	DCsc2020-02729	2021-12-30

2019-cs-R0520

中 国 计 量 科 学 研 究 院

报告编号 GXgf2021-12550
Report No.

测 试 结 果

Test Results

1. 测试条件 Test Conditions:

标准太阳电池：单晶硅；
Reference Solar Cell: mono-Si;
太阳模拟器：双光源太阳模拟器，AAA 级；
Solar Simulator Classification：double-light source in AAA classification;
温度传感器/控制系统：无；
Temperature Sensor/Control System: None;
扫描方向：正扫
Scan Direction: Forward
电压设置：-0.10V~0.90V；间隔：0.02V
Scan Parameter: From -0.10V to 0.90V with 0.02V interval
光阑 Mask（Y/N）: Y
扫描时间： 15 秒 扫描点数：51
Scan Time: 15 s Scan Point: 51

2. I-V 特性参数 I-V Characteristic parameters :

以上述标准太阳电池标定太阳模拟器辐照度至 1000 W/m²，测量所得被测太阳电池的 I-V 特性曲线和参数如下：

By using the above reference solar cell to calibrate the solar simulator's irradiance to 1000 W/m², the obtained I-V characteristic curves and parameters for samples are as follows.

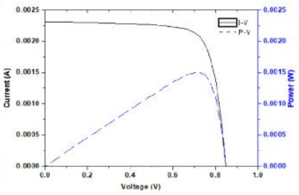

2019-cs-R0520

中 国 计 量 科 学 研 究 院

报告编号 GXgf2021-12550
Report No.

测 试 结 果

Test Results

有效面积 (mm²)	短路电流 I_{sc}(mA)	开路电压 V_{oc}(V)	最大功率 P_{max}(mW)
8.624	2.314	0.849	1.504
最大功率电流 I_{max}(mA)	最大功率电压 V_{max}(V)	填充因子 FF (%)	转换效率 η(%)
2.089	0.720	76.6	17.4

注 Note:
1. 测试所用 mask（M1）的面积为 8.624 mm²（证书编号：CDjc2019-4669）。
The mask (M1) area is 8.624 mm² (Certificate No.: CDjc2019-4669).
2. 此数据仅对被测样品当时状态有效。
The data apply only at the time of the test for the sample.
3. 英文证书摘要见附录。
Summary of certificate in English is attached in the appendix.

---------------------以下空白-------------------
Blank below

声明 Statement:
1. 我院仅对加盖“中国计量科学研究测试专用章”的完整报告负责。
NIM is ONLY responsible for the complete report with the test stamp of NIM.
2. 本报告的测试结果仅对所测试的样品有效。
The report is ONLY valid for the tested sample or instrument.
3. 本报告用中英文两种语言表述，准确含义以中文为准。
The report is expressed in both English and Chinese, with the Chinese version as standard.

测 试 员:
Tested by

核 验 员:
Checked by

2019-cs-R0520

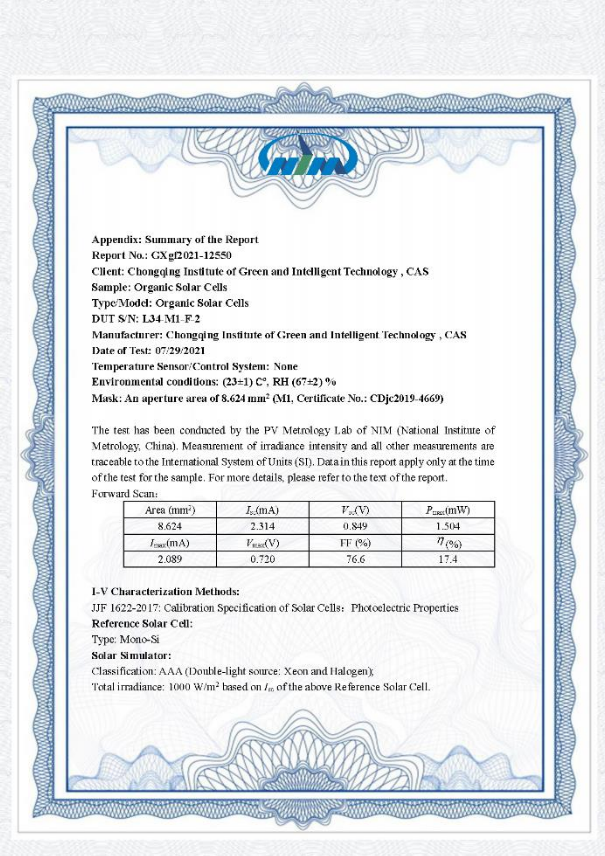

Appendix: Summary of the Report
Report No.: GXgf2021-12550
Client: Chongqing Institute of Green and Intelligent Technology , CAS
Sample: Organic Solar Cells
Type/Model: Organic Solar Cells
DUT S/N: L34-M1-F-2
Manufacturer: Chongqing Institute of Green and Intelligent Technology , CAS
Date of Test: 07/29/2021
Temperature Sensor/Control System: None
Environmental conditions: (23±1) C°, RH (67±2) %
Mask: An aperture area of 8.624 mm² (M1, Certificate No.: CDjc2019-4669)

The test has been conducted by the PV Metrology Lab of NIM (National Institute of Metrology, China). Measurement of irradiance intensity and all other measurements are traceable to the International System of Units (SI). Data in this report apply only at the time of the test for the sample. For more details, please refer to the text of the report.

Forward Scan:

Area (mm^2)	I_{sc}(mA)	V_{oc}(V)	P_{max}(mW)
8.624	2.314	0.849	1.504
I_{max}(mA)	V_{max}(V)	FF (%)	η (%)
2.089	0.720	76.6	17.4

I-V Characterization Methods:
JJF 1622-2017: Calibration Specification of Solar Cells：Photoelectric Properties
Reference Solar Cell:
Type: Mono-Si
Solar Simulator:
Classification: AAA (Double-light source: Xeon and Halogen);
Total irradiance: 1000 W/m² based on I_{sc} of the above Reference Solar Cell.

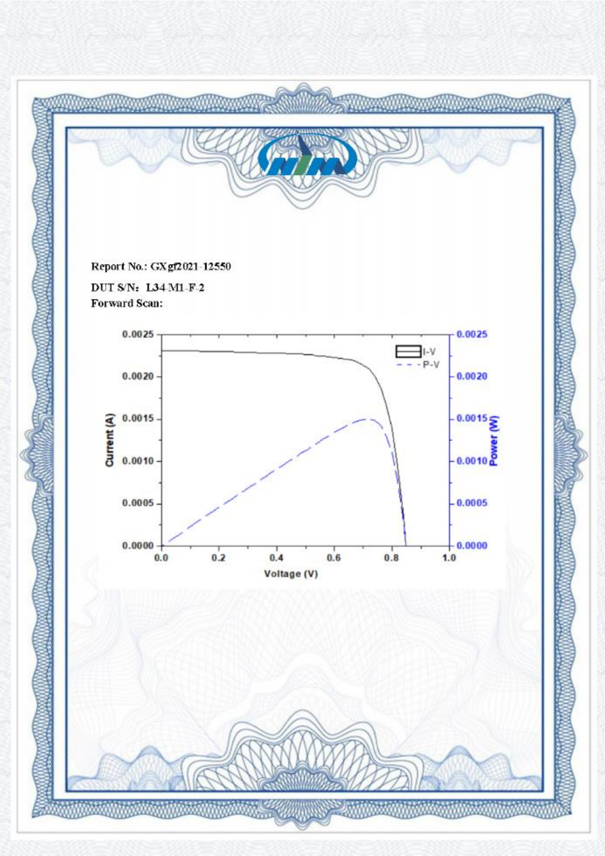

Report No.: GXgf2021-12550
DUT S/N：L34-M1-F-2
Forward Scan:

Figure S26. Independent certification by the National Institute of Metrology of PM6:BTP-m-4Cl:BTP-o-4Cl blended film solar cell confirming a stability PCE of 17.4 %.

5. Crystal data and structure of BTP-m-4Cl

Table S5. Crystal data and structure refinements for BTP-m-4Cl.

Empirical formula	$C_{90}H_{102}Cl_4N_8O_2S_5$
Formula weight	1629.89
Temperature	173.0 K
Wavelength	1.34139 Å
Crystal system	Monoclinic
Space group	C 1 2/c 1
a	25.4283(17) Å
b	22.6716(16) Å
c	31.893(2) Å
α	90°.
β	104.608(3)°.
γ	90°.
Volume	17792(2) $Å^3$
Z	8
Density (calculated)	1.217 Mg/m^3
Absorption coefficient	1.758 mm^{-1}
F(000)	6896
Crystal size	0.1 x 0.08 x 0.05 mm^3
Theta range for data collection	3.471 to 55.396°.
Index ranges	-30<=h<=31, -27<=k<=19, -39<=l<=32
Reflections collected	91013
Independent reflections	16884 [R(int) = 0.0527]
Completeness to theta = 53.594°	99.2 %
Absorption correction	Semi-empirical from equivalents
Max. and min. transmission	0.7508 and 0.4041
Refinement method	Full-matrix-block least-squares on F^2
Data / restraints / parameters	16884 / 319 / 976
Goodness-of-fit on F^2	1.222
Final R indices [I>2sigma(I)]	R1 = 0.1361, wR2 = 0.3289
R indices (all data)	R1 = 0.1910, wR2 = 0.3657
Extinction coefficient	n/a
Largest diff. peak and hole	0.646 and -0.352 e.$Å^{-3}$

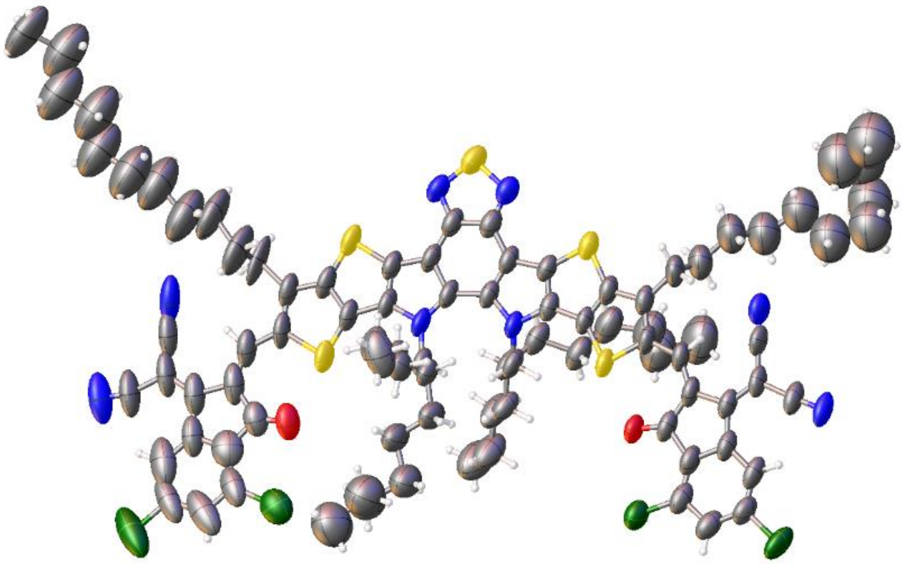

Figure S27. ORTEP drawing of the atomic distribution for the elementary unit of BTP-m-4Cl.

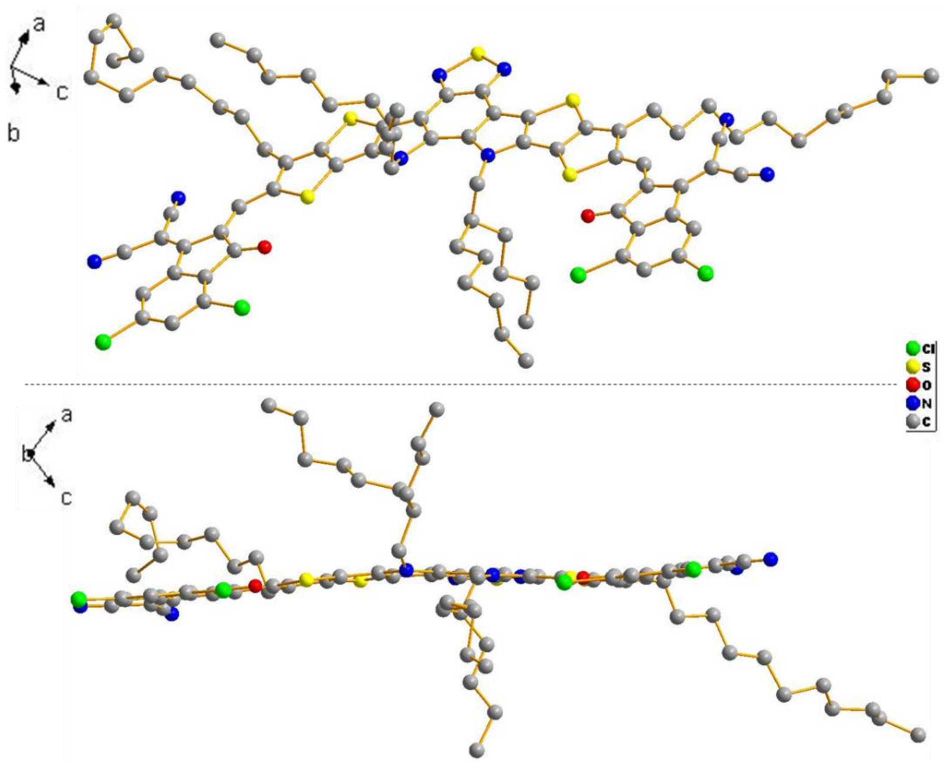

Figure S28. Perspective views of atomic distribution for elementary unit of BTP-m-4Cl molecule in different directions. The hydrogen atoms are omitted for clarity.

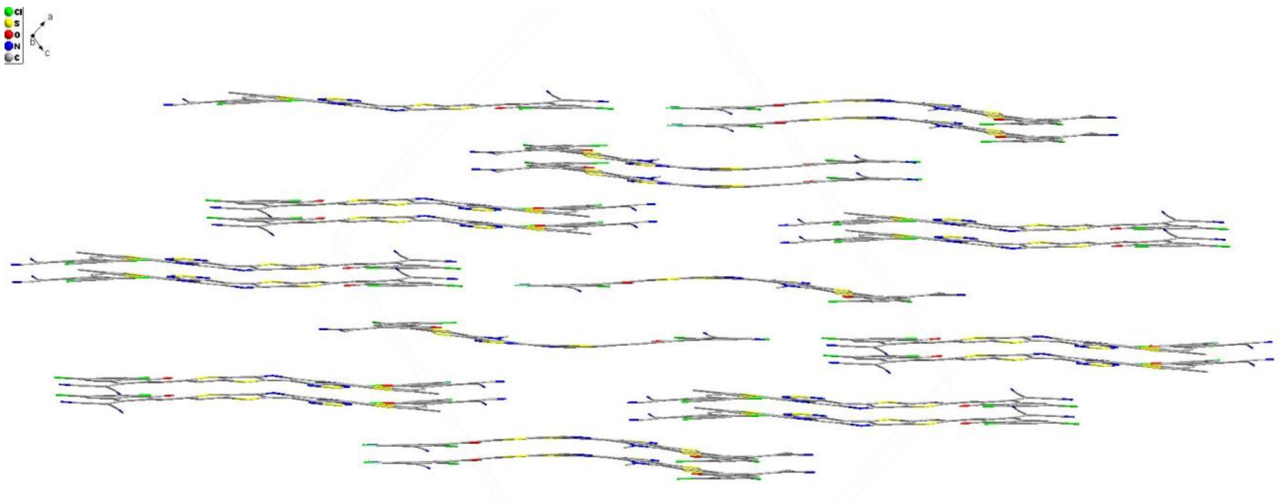

Figure S29. Perspective view of the lamellar staking of BTP-m-4Cl molecules.

The alkyl chains are omitted for clarity.

6. The synthesis route of reported materials for SC calculation

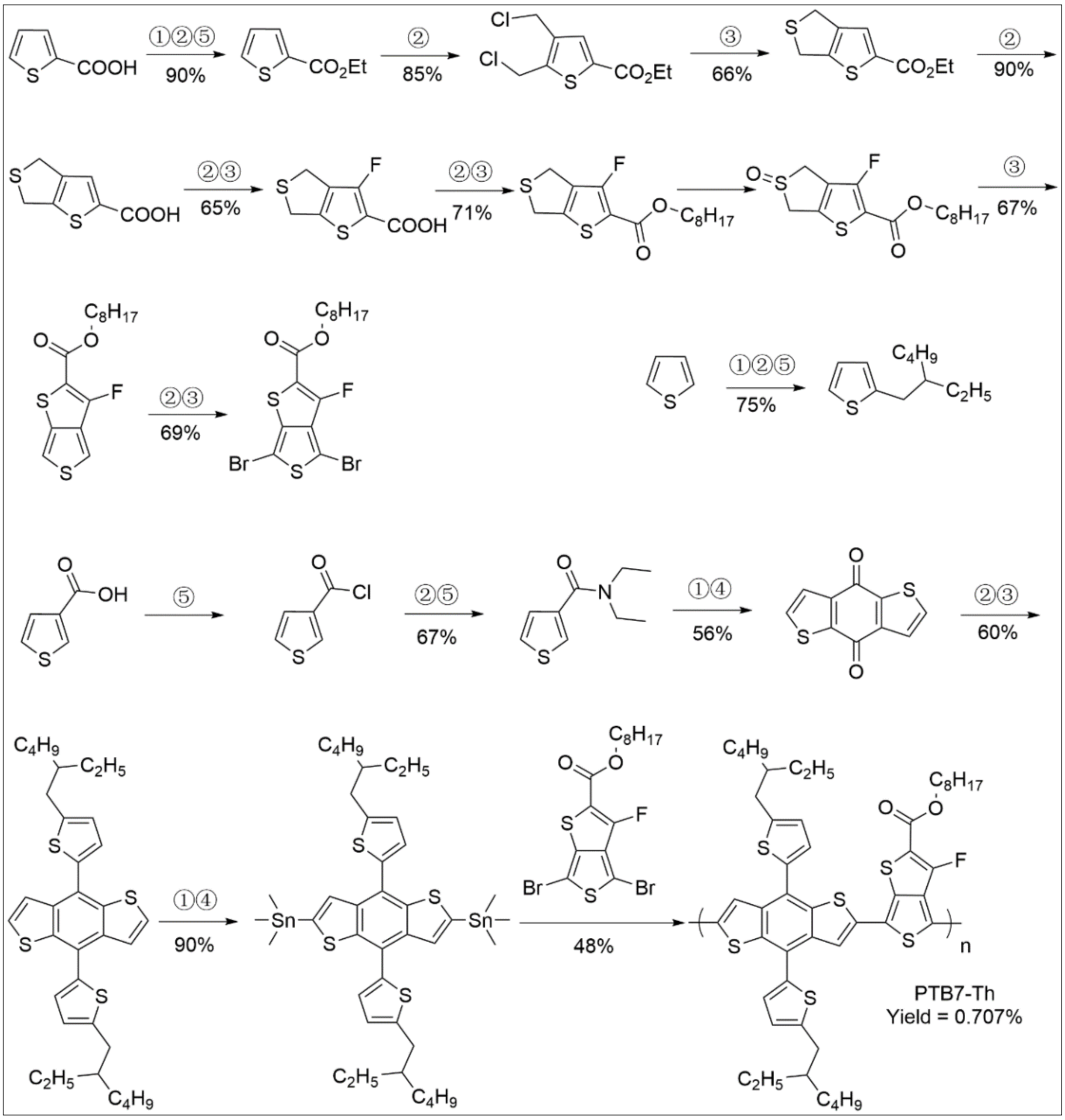

Figure S30. Synthetic route of PM7-Th reported in Ref.[3].

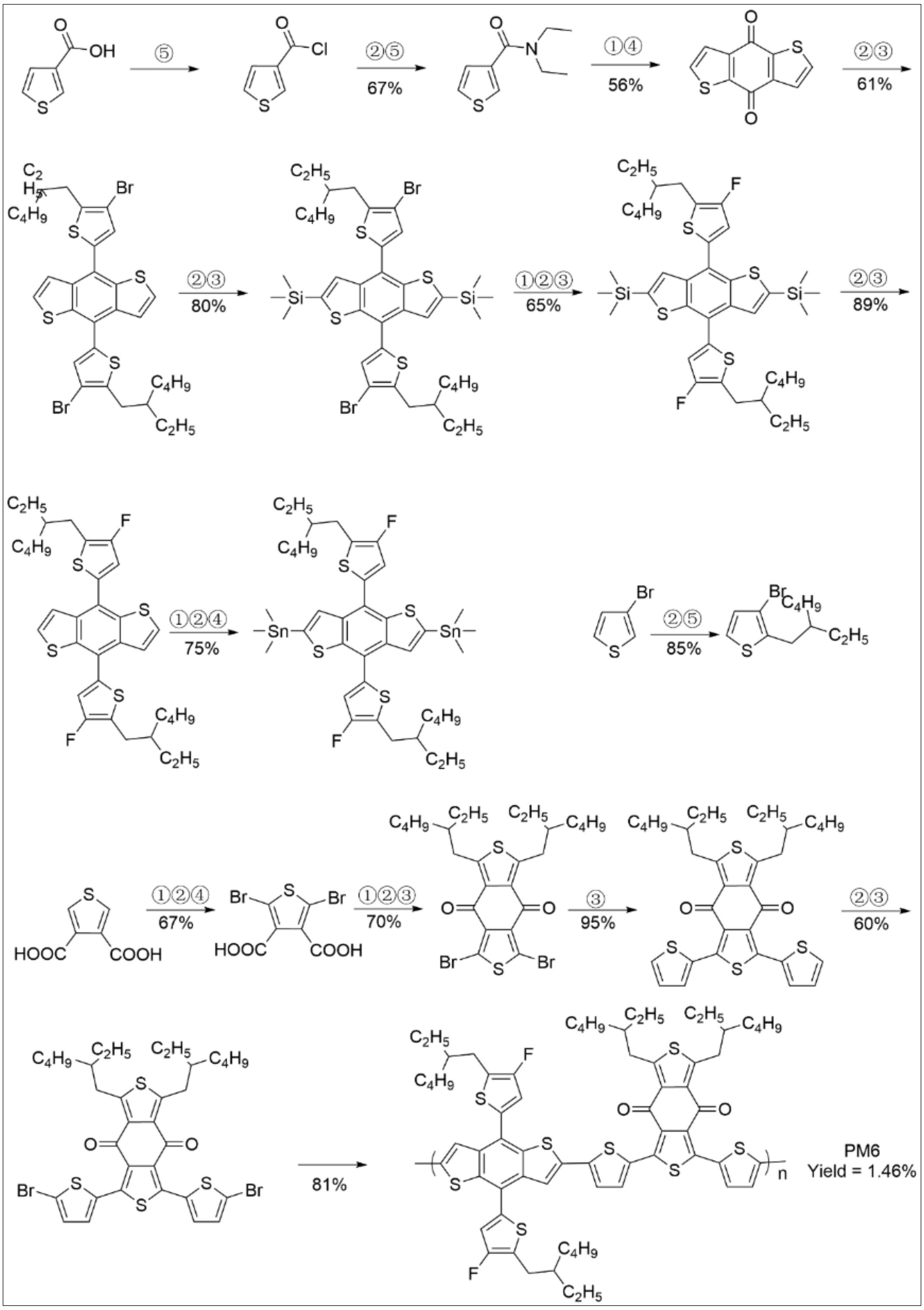

Figure S31. Synthetic route of PM6 reported in Ref.[4].

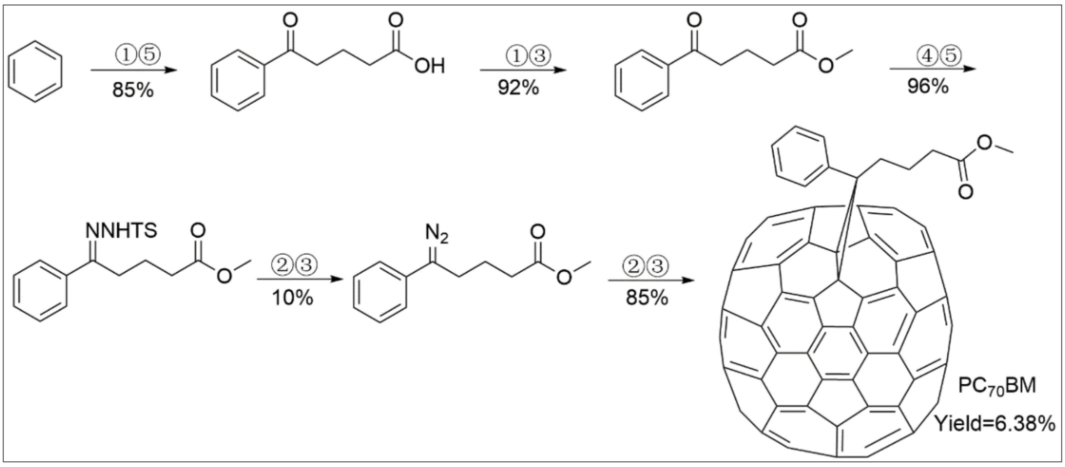

Figure S32. Synthetic route of $PC_{70}BM$ reported in Ref.[5].

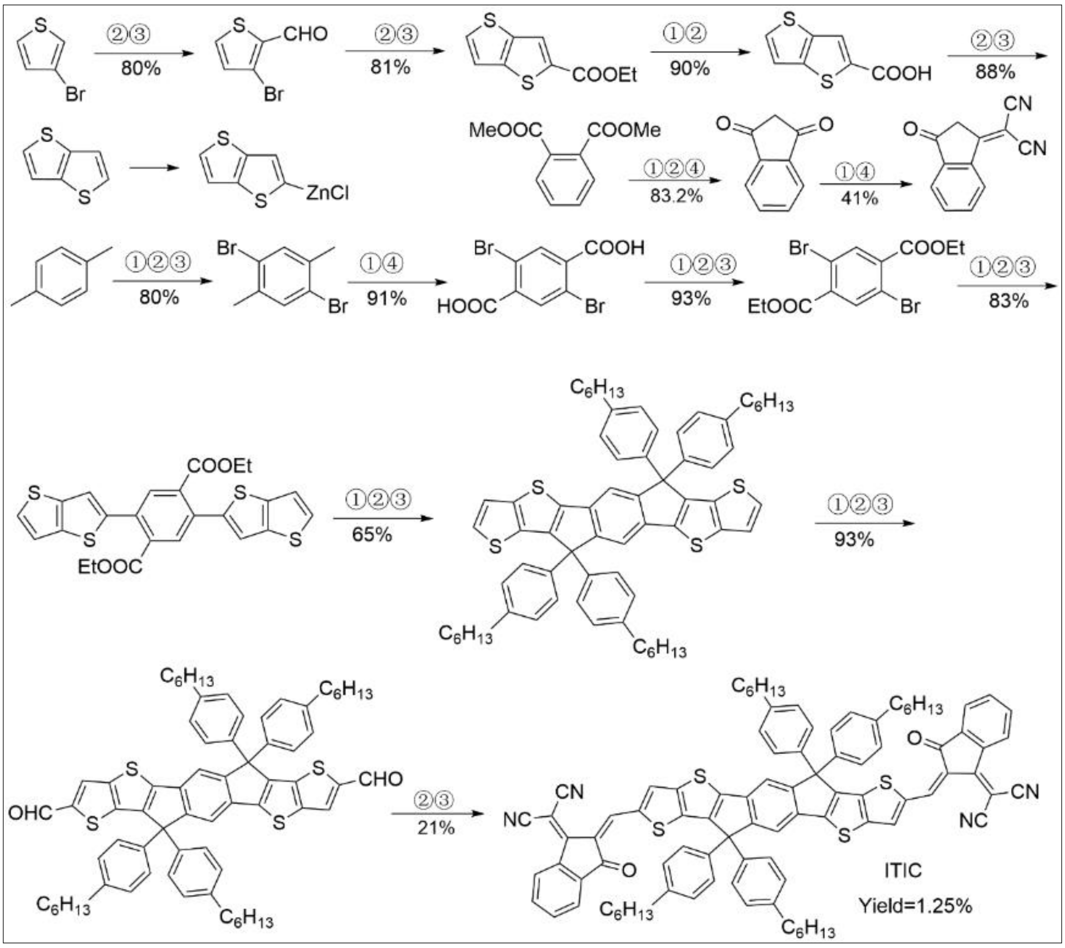

Figure S33. Synthetic route of ITIC reported in Ref.[6].

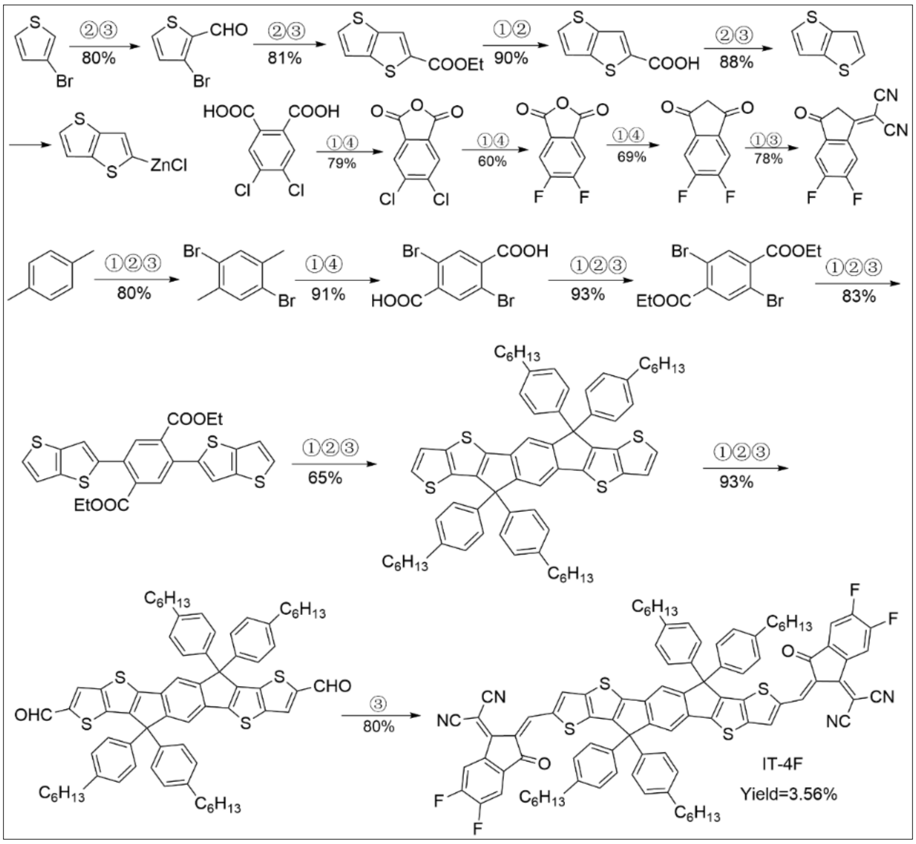

Figure S34. Synthetic route of IT-4F reported in Ref.[7].

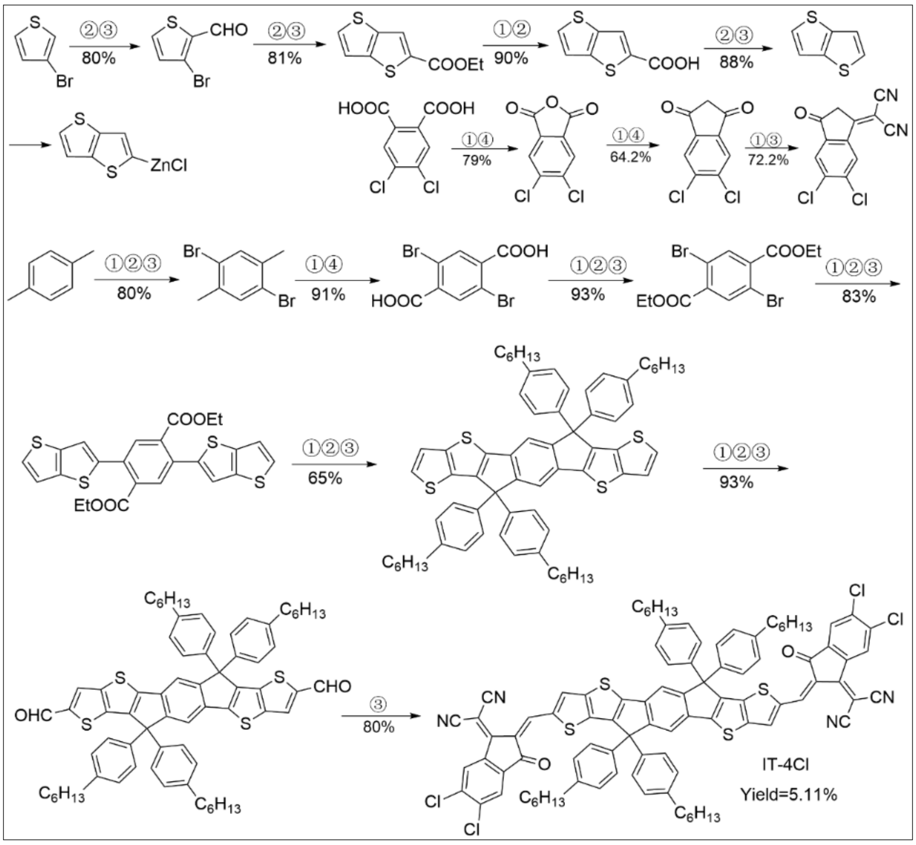

Figure S35. Synthetic route of IT-4Cl reported in Ref.[8].

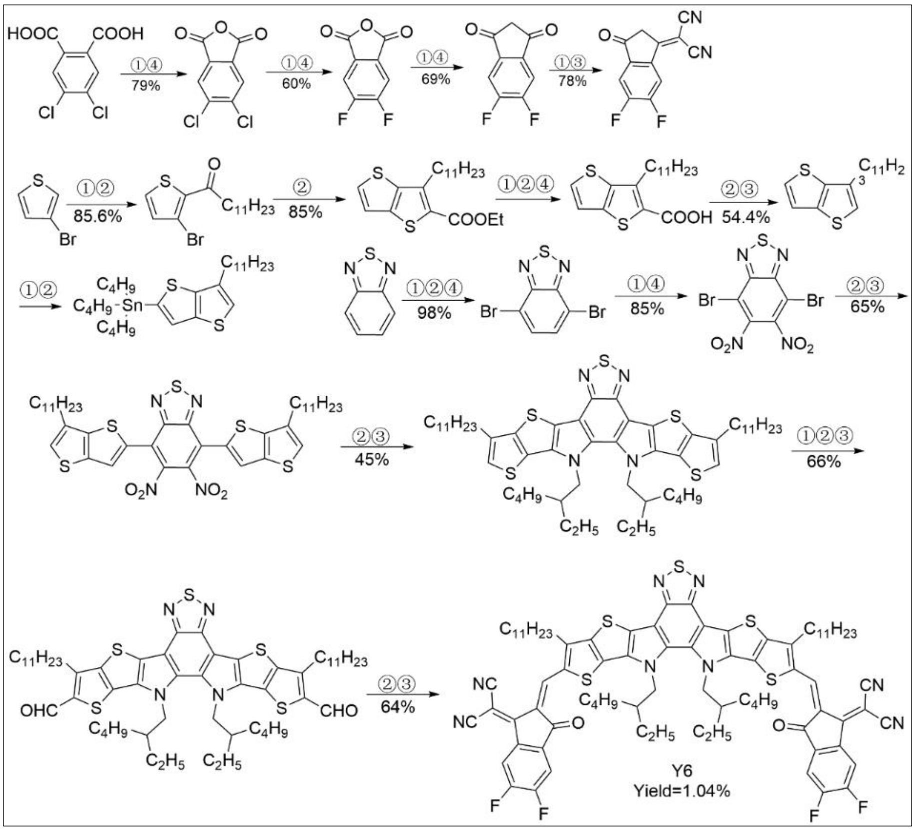

Figure S36. Synthetic route of Y6 reported in Ref.[9].

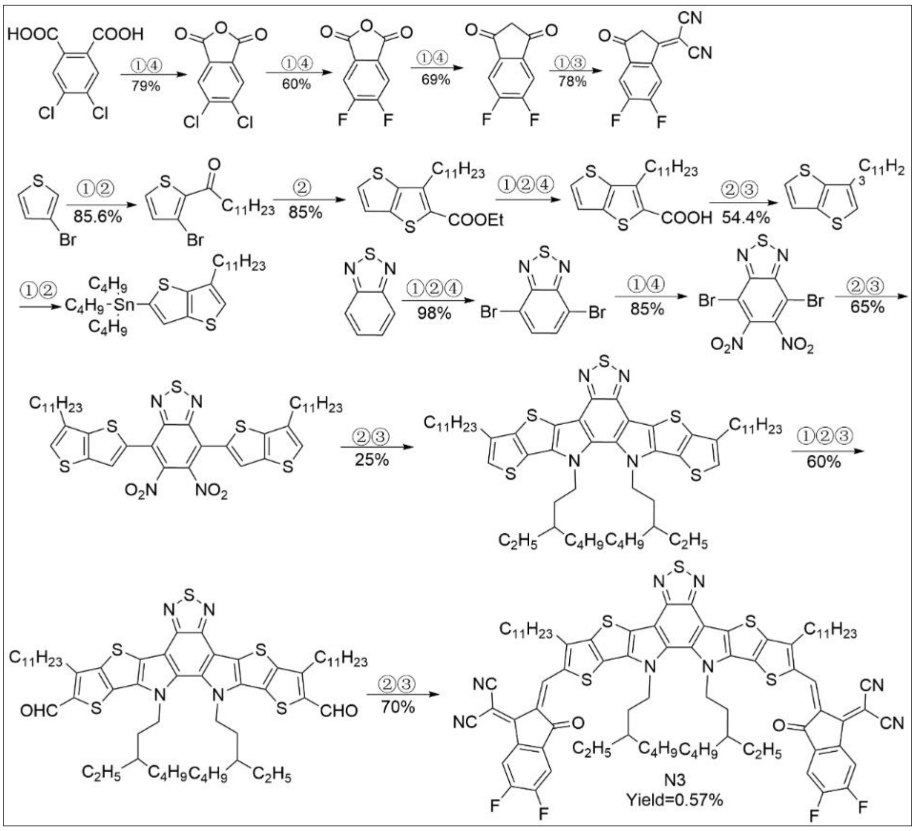

Figure S37. Synthetic route of N3 reported in Ref.[10].

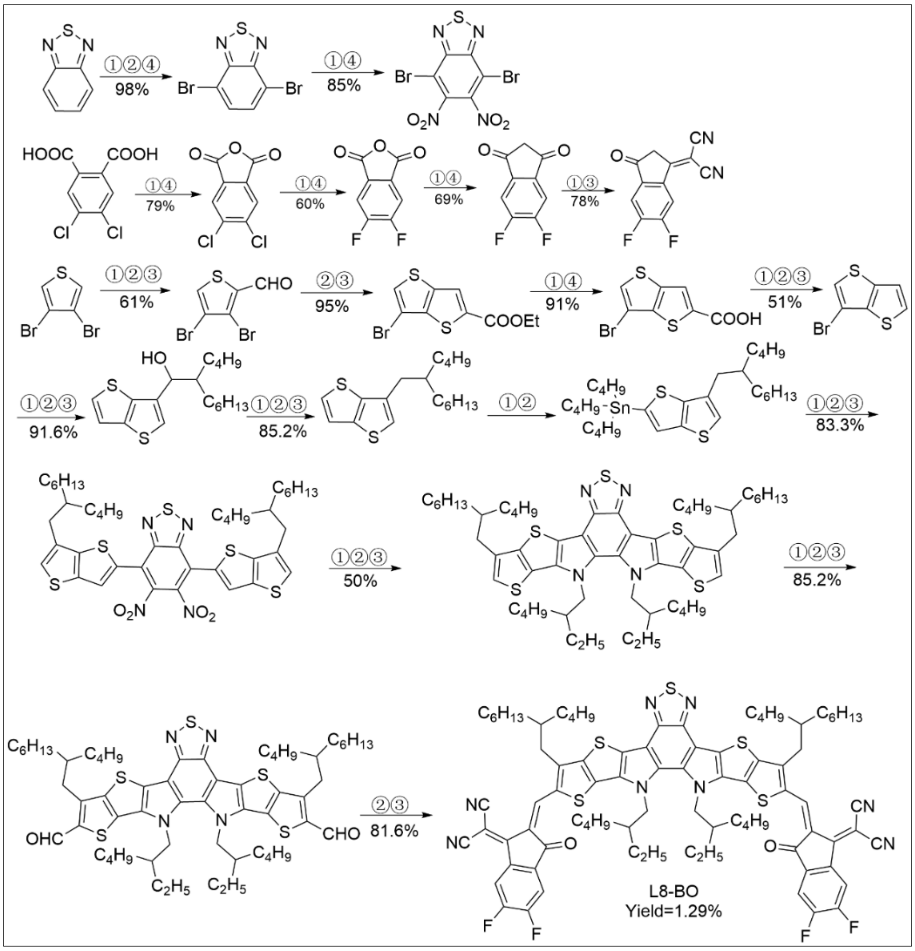

Figure S38. Synthetic route of L8-BO reported in Ref.[11].

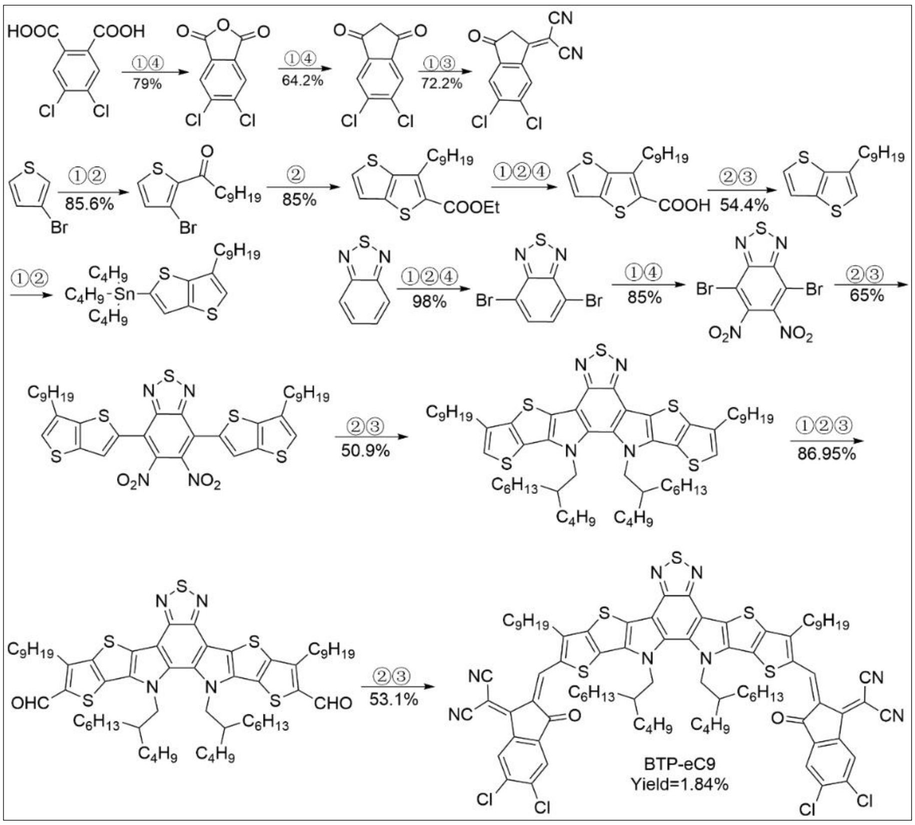

Figure S39. Synthetic route of BTP-eC9 reported in Ref.[12].

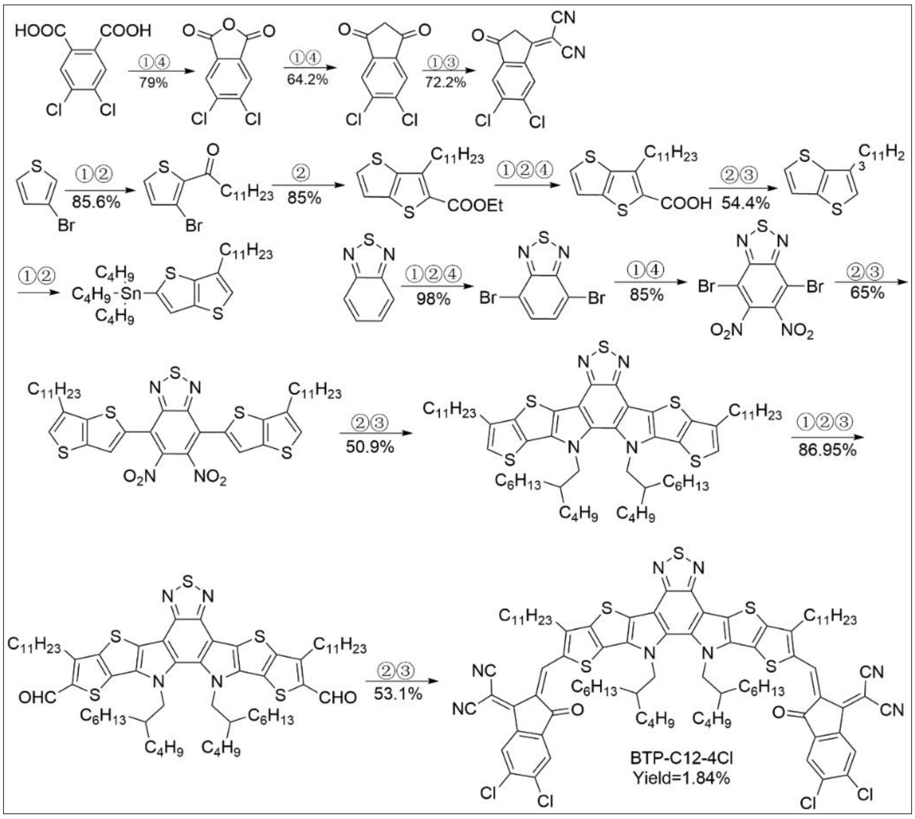

Figure S40. Synthetic route of BTP-C12-4Cl reported in Ref.[13].

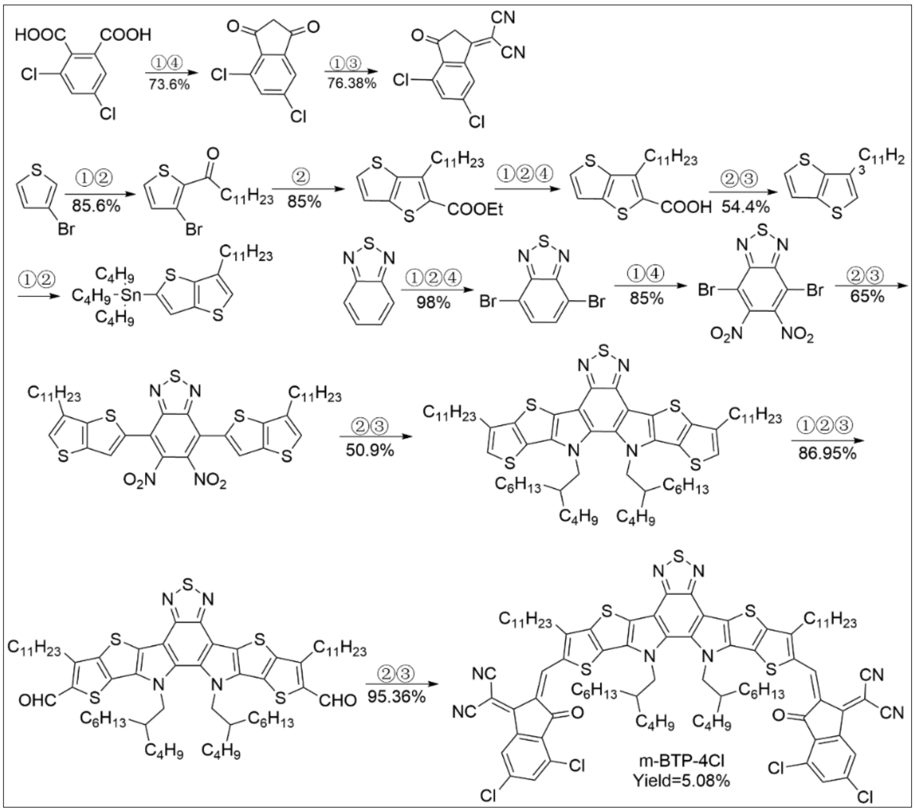

Figure S41. Synthetic route of BTP-m-4Cl reported in this work.

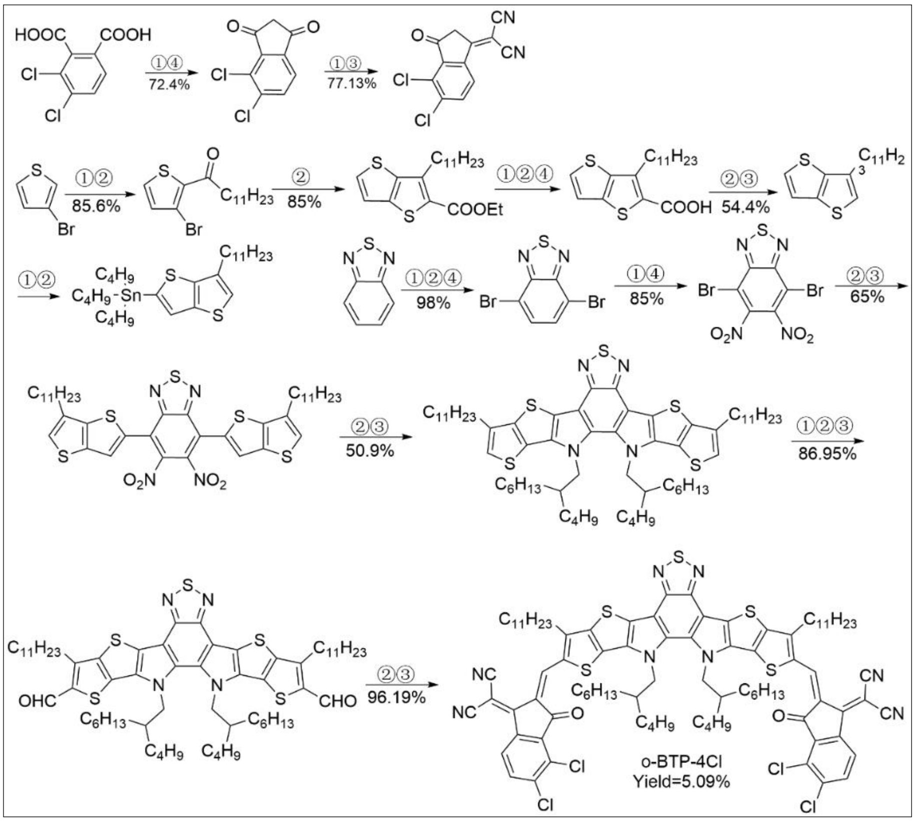

Figure S42. Synthetic route of BTP-o-4Cl reported in this work.

7. Calculation of SC and FOM index

Table S6. Summary of SC and correlation indexes of representative acceptors and their donor partners.

Material	NSS	RY	NUO	NCC	NHC	SC	Refs.
PTB7-Th	16	0.0707	24	7	31	86.327	[3]
PM6	14	0.0146	20	7	22	98.728	[4]
$PC_{71}BM$	5	0.0638	9	3	6	44.116	[5]
ITIC	12	0.0125	23	9	26	94.442	[6]
IT-4F	16	0.0356	24	10	30	97.193	[7]
IT-4Cl	15	0.0511	22	10	28	91.162	[8]
Y6	15	0.0104	26	6	25	100.402	[9]
N3	15	0.0057	26	6	25	105.450	[10]
L8-BO	17	0.0129	32	10	29	111.524	[11]
BTP-eC9	14	0.0184	24	6	23	92.616	[12]
BTP-C12-4Cl	14	0.0184	24	6	23	92.616	[13]
**BTP-m-4Cl**	**13**	**0.0508**	**23**	**6**	**21**	**81.562**	**This Work**
**BTP-o-4Cl**	**13**	**0.0509**	**23**	**6**	**21**	**81.546**	

The SC is calculated according to the following equation 1:[14]

$$SC = 35\frac{NSS}{NSS_{max}} + 25\frac{\log(RY)}{\log(RY_{max})} + 15\frac{NUO}{NUO_{max}} + 15\frac{NCC}{NCC_{max}} + 10\frac{NHC}{NHC_{max}} \qquad \text{(Equation 1)}$$

NSSmax = 17; RYmax = 0.0707; NUOmax = 26; NCCmax = 10; NHCmax = 31.

Table S7. Summary of PCE, SC and FOM indexes of representative OSC devices.

Material	$Ratio_{D:A}$	$SC_{D:A}$	PCE(%)	FOM	Refs.
PTB7-Th:$PC_{71}BM$	1:1.5	61.001	9.35	0.153	[3]
PTB7-Th:ITIC	1:1.3	90.913	6.8	0.075	[6]
PM6:IT-4F	1:1	97.960	13.2	0.135	[7]
PM6:IT-4Cl	1:1	94.945	13.45	0.142	[8]
PM6:Y6	1:1.2	99.641	15.7	0.158	[9]
PM6:N3	1:1.2	103.331	15.98	0.155	[10]
PM6:L8-BO	1:1.2	106.643	18.32	0.172	[11]
PM6:BTP-eC9	1:1.2	96.330	17.8	0.185	[12]
PM6:BTP-C12-4Cl	1:1.2	96.330	17	0.176	[13]
**PM6:BTP-m-4Cl**	**1:1.3**	**89.921**	**17.25**	**0.190**	**This Work**
**PM6:BTP-o-4Cl**	**1:1.3**	**89.912**	**16.04**	**0.178**	
**PM6:BTP-m-4Cl:BTP-o-4Cl**	**1:1:0.3**	**89.023**	**17.95**	**0.202**	

The $SC_{D:A}$ is calculated according to the following equation 2:[14b]

$$SC_{D:A} = \frac{SC_D \times W_D + SC_A \times W_A}{W_D + W_A} \quad \text{(Equation 2)}$$

The FOM is calculated according to the following equation 3:[14b]

$$FOM = \frac{PCE}{SC_{D:A}} \quad \text{(Equation 3)}$$

8. The detailed fabrication of OSC devices

6.1 The detailed fabrication of PM6: BTP-m-4Cl based devices

Table S8. The photovoltaic data of PM6:BTP-m-4Cl ratio is 1:1.3 solar cells with different conditions. All data were obtained under illumination of AM 1.5G (100mW/cm^2) light source.

Condition	V_{OC} [V]	J_{SC} [mA/cm^2]	FF [%]	$^{a)}$PCE [%]
As cast	0.868 (0.860±0.008)	26.19 (26.21±0.02)	69.19 (67.32±1.87)	15.73 (15.18±0.55)
90°C, 10min	0.864 (0.857±0.007)	26.29 (26.14±0.14)	69.65 (68.88±0.77)	15.82 (15.43±0.39)
100°C, 10min	0.858 (0.851±0.007)	26.27 (26.20±0.07)	68.13 (67.49±0.63)	15.35 (15.05±0.31)
110°C, 10min	0.844 (0.843±0.001)	26.00 (25.96±0.04)	68.23 (66.52±1.71)	14.97 (14.55±0.42)

a) The average parameters were calculated over 10 independent cells.

Table S9. The photovoltaic data of PM6:BTP-M-4Cl ratio is 1:1.3 solar cells with different conditions. All data were obtained under illumination of AM 1.5G (100mW/cm^2) light source.

Condition	V_{OC} [V]	J_{SC} [mA/cm^2]	FF [%]	$^{a)}$PCE [%]
CS_2,20s, 90°C,5min	0.851 (0.845±0.006)	26.52 (26.42±0.10)	72.14 (71.49±0.65)	16.28 (16.00±0.28)
CS_2,30s, 90°C,5min	0.857 (0.849±0.008)	26.31 (26.13±0.18)	73.56 (72.17±1.40)	16.58 (16.03±0.55)
CS_2,40s, 90°C,5min	0.858 (0.851±0.007)	26.64 (26.16±0.48)	73.27 (72.37±0.89)	16.70 (16.14±0.56)
CS_2,50s, 90°C,5min	0.861 (0.853±0.008)	26.34 (26.10±0.23)	72.40 (70.86±1.53)	16.43 (15.78±0.66)
CS_2,60s, 90°C,5min	0.846 (0.849±0.003)	25.57 (25.27±0.30)	74.94 (73.43±1.51)	16.21 (15.76±0.44)
CS_2,70s, 90°C,5min	0.855 (0.850±0.005)	25.49 (25.20±0.29)	73.22 (72.96±0.25)	15.97 (15.64±0.33)

a) The average parameters were calculated over 10 independent cells.

Table S10. Under the optimal conditions (CS_2, 40s, TA 5min at 90°C), the photovoltaic data of different ratios of PM6:BTP-m-4Cl solar cells. All data were obtained under the illumination of AM 1.5G (100mW/cm^2) light source.

D:A	V_{OC} [V]	J_{SC} [mA/cm^2]	FF [%]	$^{a)}$PCE [%]
1:1.0	0.847 (0.841±0.005)	26.57 (26.49±0.08)	70.10 (69.46±0.64)	15.78 (15.48±0.30)
1:1.1	0.850 (0.844±0.005)	26.45 (26.42±0.02)	72.59 (71.05±0.46)	16.31 (15.86±0.46)

D:A	V_{OC} [V]	J_{SC} [mA/cm^2]	FF [%]	[a)]PCE [%]
1:1.2	0.850 (0.841±0.008)	26.63 (26.54±0.09)	72.97 (71.88±1.08)	16.51 (16.05±0.46)
1:1.3	0.860 (0.852±0.008)	26.37 (26.24±0.13)	72.95 (72.57±0.38)	16.55 (16.23±0.32)
1:1.4	0.840 (0.839±0.001)	26.52 (26.46±0.05)	72.03 (70.13±1.89)	16.04 (15.57±0.47)
1:1.5	0.836 (0.839±0.003)	26.25 (26.10±0.15)	69.98 (69.47±0.51)	15.36 (15.22±0.13)

a) The average parameters were calculated over 10 independent cells.

6.2 The detailed fabrication of PM6: BTP-o-4Cl based devices

Table S11. The photovoltaic data of PM6:BTP-o-4Cl ratio is 1:1.3 solar cells with different conditions. All data were obtained under the illumination of AM 1.5G (100mW/cm^2) light source.

Condition	V_{OC} [V]	J_{SC} [mA/cm^2]	FF [%]	[a)]PCE [%]
As cast	0.902 (0.899±0.003)	22.78 (22.32±0.46)	66.76 (65.83±0.93)	13.72 (13.21±0.51)
90℃, 10min	0.907 (0.892±0.015)	24.03 (24.55±0.52)	69.79 (67.44±2.35)	15.21 (14.76±0.45)
100℃, 10min	0.895 (0.889±0.006)	24.04 (24.01±0.03)	68.21 (66.92±1.30)	14.68 (14.29±0.39)
110℃, 10min	0.888 (0.883±0.005)	23.79 (23.82±0.03)	66.93 (65.91±1.02)	14.14 (13.87±0.27)

a) The average parameters were calculated over 10 independent cells.

Table S12. The photovoltaic data of PM6:BTP-o-4Cl ratio is 1:1.3 solar cells with different conditions. All data were obtained under the illumination of AM 1.5G (100mW/cm^2) light source.

Condition	V_{OC} [V]	J_{SC} [mA/cm^2]	FF [%]	[a)]PCE [%]
CS_2,30s, 90℃,5min	0.902 (0.898±0.004)	24.32 (24.11±0.22)	70.60 (70.96±0.36)	15.49 (15.36±0.13)
CS_2,40s, 90℃,5min	0.907 (0.895±0.012)	23.79 (23.95±0.15)	73.97 (71.92±2.05)	15.96 (15.41±0.55)
CS_2,50s, 90℃,5min	0.908 (0.899±0.008)	24.12 (23.90±0.21)	73.20 (72.96±0.25)	16.03 (15.68±0.35)
CS_2,60s, 90℃,5min	0.894 (0.893±0.001)	24.46 (24.00±0.46)	73.29 (72.77±0.52)	16.07 (15.60±0.47)
CS_2,70s, 90℃,5min	0.885 (0.890±0.005)	24.02 (24.17±0.14)	74.46 (72.15±2.31)	15.84 (15.52±0.31)

a) The average parameters were calculated over 10 independent cells.

Table S13. Under the optimal conditions (CS_2, 60s, TA 5min at 90℃), the photovoltaic data of different ratios of PM6:BTP-o-4Cl solar cells. All data were obtained under the illumination of AM 1.5G (100mW/cm^2) light source.

D: A	V_{OC} [V]	J_{SC} [mA/cm^2]	FF [%]	[a]PCE [%]
1:1.0	0.886 (0.885±0.001)	24.33 (24.18±0.14)	70.14 (69.86±0.28)	15.11 (14.93±0.17)
1:1.2	0.887 (0.886±0.001)	24.22 (24.20±0.02)	72.08 (70.83±1.25)	15.49 (15.20±0.28)
1:1.3	0.904 (0.895±0.009)	24.22 (24.15±0.07)	73.68 (71.99±1.68)	16.14 (15.56±0.58)
1:1.4	0.889 (0.883±0.006)	24.34 (24.68±0.33)	72.92 (71.32±1.60)	15.78 (15.54±0.24)
1:1.5	0.886 (0.879±0.007)	24.35 (24.17±0.19)	71.31 (24.17±0.19)	15.39 (14.96±0.44)

a) The average parameters were calculated over 10 independent cells.

6.3 The detailed fabrication of PM6: BTP-m-4Cl : BTP-o-4Cl based devices

Table S14. The photovoltaic data of the PM6:BTP-m-4Cl: BTP-o-4Cl (the ratio is 1:1.1:0.2) solar cells with different conditions. All data were obtained under the illumination of AM 1.5G (100mW/cm^2) light source.

Condition D: A1: A2 (1:1.1:0.2)	V_{OC} [V]	J_{SC} [mA/cm^2]	FF [%]	[a]PCE [%]
As cast	0.862 (0.859±0.002)	26.04 (25.71±0.33)	67.02 (66.07±0.94)	15.05 (14.61±0.44)
CS2,30s,90,5min	0.851 (0.850±0.001)	26.39 (26.16±0.24)	70.66 (68.47±2.18)	15.86 (15.26±0.61)
CS2,40s,90,5min	0.862 (0.856±0.006)	26.76 (26.27±0.48)	72.48 (71.00±1.48)	16.74 (15.98±0.76)
CS2,50s,90,5min	0.860 (0.852±0.007)	26.31 (25.94±0.37)	72.54 (71.05±1.49)	16.43 (15.72±0.70)

a) The average parameters were calculated over 10 independent cells.

Table S15. The photovoltaic data of the PM6:BTP-m-4Cl: BTP-o-4Cl (the ratio is 1:1:0.3) solar cells with different condition. All data were obtained under the illumination of AM 1.5G (100mW/cm^2) light source.

Condition D: A1: A2 (1:1:0.3)	V_{OC} [V]	J_{SC} [mA/cm^2]	FF [%]	[a]PCE [%]
As cast	0.870 (0.873±0.003)	25.45 (25.24±0.21)	69.06 (67.83±1.22)	15.29 (14.95±0.34)
CS2,20s,90,5min	0.859 (0.854±0.004)	26.95 (26.50±0.45)	72.42 (71.41±1.01)	16.76 (16.17±0.59)

Condition D: A1: A2 (1:1:0.3)	V_{OC} [V]	J_{SC} [mA/cm^2]	FF [%]	$^{a)}$PCE [%]
CS2,30s,90,5min	0.865 (0.858±0.007)	26.80 (26.71±0.09)	73.40 (72.59±0.82)	17.02 (16.64±0.38)
CS2,40s,90,5min	0.849 (0.850±0.001)	26.88 (26.76±0.11)	72.41 (71.73±0.68)	16.52 (16.34±0.18)
CS2,50s,90,5min	0.848 (0.850±0.002)	26.40 (26.30±0.10)	72.83 (72.11±0.71)	16.30 (16.14±0.16)

a) The average parameters were calculated over 10 independent cells.

Table S16. The photovoltaic data of the PM6: BTP-m-4Cl: BTP-o-4Cl (the ratio is 1:0.9:0.4) solar cells with different conditions. All data were obtained under the illumination of AM 1.5G (100mW/cm^2) light source.

Condition D: A1: A2 (1:0.9:0.4)	V_{OC} [V]	J_{SC} [mA/cm^2]	FF [%]	$^{a)}$PCE [%]
As cast	0.875 (0.871±0.004)	25.08 (24.94±0.15)	68.70 (68.60±0.10)	15.09 (14.89±0.19)
CS2,30s,90,5min	0.852 (0.850±0.002)	26.29 (26.23±0.06)	72.29 (72.24±0.05)	16.19 (16.11±0.08)
CS2,40s,90,5min	0.854 (0.855±0.001)	25.93 (25.90±0.03)	74.12 (73.05±1.08)	16.43 (16.21±0.22)
CS2,50s,90,5min	0.854 (0.851±0.003)	26.33 (26.25±0.08)	74.45 (73.15±1.30)	16.74 (16.36±0.38)

a) The average parameters were calculated over 10 independent cells.

Table S17. The photovoltaic data of the PM6:BTP-m-4Cl: BTP-o-4Cl (the ratio is 1:8:0.5) solar cells with different conditions. All data were obtained under the illumination of AM 1.5G (100mW/cm^2) light source.

Condition D: A1: A2 (1:0.8:0.5)	V_{OC} [V]	J_{SC} [mA/cm^2]	FF [%]	$^{a)}$PCE [%]
As cast	0.866 (0.868±0.002)	25.78 (25.69±0.09)	69.03 (66.93±2.10)	15.40 (14.93±0.47)
CS2,30s,90,5min	0.854 (0.853±0.001)	26.40 (26.41±0.01)	71.97 (70.50±1.47)	16.23 (15.91±0.32)
CS2,40s,90,5min	0.864 (0.855±0.008)	26.53 (26.51±0.02)	71.50 (71.30±0.20)	16.38 (16.17±0.22)
CS2,50s,90,5min	0.853 (0.857±0.004)	25.68 (25.60±0.08)	71.11 (70.71±0.40)	15.57 (15.51±0.06)
CS2,60s,90,5min	0.871 (0.864±0.007)	23.41 (23.21±0.20)	72.59 (71.36±1.23)	14.80 (14.31±0.49)

a) The average parameters were calculated over 10 independent cells.

Table S18. The photovoltaic data of the PM6:BTP-m-4Cl: BTP-o-4Cl (the ratio is 1:0.7:0.6) solar cells with different conditions. All data were obtained under the illumination of AM 1.5G (100mW/cm^2) light source.

Condition D: A1: A2 (1:0.7:0.6)	V_{OC} [V]	J_{SC} [mA/cm^2]	FF [%]	[a]PCE [%]
As cast	0.887 (0.876±0.009)	24.97 (24.88±0.09)	69.28 (68.57±0.71)	15.34 (14.96±0.38)
CS2,20s,90,5min	0.860 (0.858±0.002)	25.65 (25.70±0.05)	70.05 (68.09±1.96)	15.45 (15.02±0.43)
CS2,30s,90,5min	0.865 (0.865±0.003)	25.77 (25.82±0.05)	71.64 (69.90±1.74)	15.97 (15.62±0.35)
CS2,40s,90,5min	0.874 (0.863±0.011)	25.82 (25.71±0.12)	71.60 (71.08±0.39)	16.16 (15.77±0.39)
CS2,50s,90,5min	0.875 (0.870±0.005)	24.37 (23.94±0.43)	70.81 (71.28±0.47)	15.11 (14.85±0.26)

a) The average parameters were calculated over 10 independent cells.

Table S19. The photovoltaic data of the PM6:BTP-m-4Cl: BTP-o-4Cl (the ratio is 1:0.6:0.7) solar cells with different conditions. All data were obtained under the illumination of AM 1.5G (100mW/cm^2) light source.

Condition D: A1: A2 (1:0.6:0.7)	V_{OC} [V]	J_{SC} [mA/cm^2]	FF [%]	[a]PCE [%]
As cast	0.893 (0.887±0.006)	24.35 (24.51±0.15)	68.51 (67.61±0.90)	14.90 (14.68±0.21)
CS2,20s,90,5min	0.869 (0.867±0.002)	25.64 (25.55±0.08)	73.15 (71.31±1.84)	16.30 (15.80±0.50)
CS2,30s,90,5min	0.870 (0.868±0.002)	25.77 (25.56±0.21)	71.89 (70.30±1.59)	16.12 (15.64±0.48)
CS2,40s,90,5min	0.870 (0.869±0.001)	25.63 (25.61±0.02)	71.83 (69.41±2.42)	16.03 (15.45±0.57)

a) The average parameters were calculated over 10 independent cells.

Table S20. The photovoltaic data of the PM6:BTP-m-4Cl: BTP-o-4Cl (the ratio is 1:0.5:0.8) solar cells with different conditions. All data were obtained under the illumination of AM 1.5G (100mW/cm^2) light source.

Condition D: A1: A2 (1:0.5:0.8)	V_{OC} [V]	J_{SC} [mA/cm^2]	FF [%]	[a]PCE [%]
As cast	0.885 (0.884±0.001)	24.87 (24.83±0.04)	69.11 (67.23±1.88)	15.22 (14.76±0.46)
CS2,20s,90,5min	0.876 (0.873±0.003)	25.82 (25.59±0.23)	72.58 (70.44±2.15)	16.43 (15.73±0.69)
CS2,30s,90,5min	0.873 (0.872±0.001)	25.23 (25.13±0.10)	72.33 (70.57±1.76)	15.94 (15.48±0.46)
CS2,40s,90,5min	0.877 (0.875±0.002)	26.02 (25.61±0.41)	70.47 (69.87±0.60)	16.08 (15.66±0.42)
CS2,50s,90,5min	0.876 (0.874±0.002)	25.23 (25.13±0.10)	71.58 (70.54±1.05)	15.83 (15.51±0.32)

a) The average parameters were calculated over 10 independent cells.

Table S21. The photovoltaic data of the PM6:BTP-m-4Cl:BTP-o-4Cl (the ratio is 1:0.4:0.9) solar cells with different conditions. All data were obtained under the illumination of AM 1.5G ($100mW/cm^2$) light source.

Condition D: A1: A2 (1:0.4:0.9)	V_{OC} [V]	J_{SC} [mA/cm^2]	FF [%]	a)PCE [%]
As cast	0.907 (0.901±0.006)	24.07 (24.04±0.03)	68.01 (67.15±0.85)	14.86 (14.55±0.31)
CS2,20s,90,5min	0.884 (0.878±0.005)	25.46 (25.49±0.03)	70.01 (69.04±0.97)	15.75 (15.45±0.30)
CS2,30s,90,5min	0.886 (0.880±0.006)	25.16 (25.25±0.09)	71.86 (70.49±1.37)	16.04 (15.66±0.37)
CS2,40s,90,5min	0.886 (0.880±0.0016)	24.97 (25.02±0.05)	69.86 (68.78±1.07)	15.47 (15.15±0.31)

a) The average parameters were calculated over 10 independent cells.

Table S22. The photovoltaic data of the PM6:BTP-m-4Cl:BTP-o-4Cl (the ratio is 1:0.3:1.0) solar cells with different conditions. All data were obtained under the illumination of AM 1.5G ($100mW/cm^2$) light source.

Condition D: A1: A2 (1:0.3:1.0)	V_{OC} [V]	J_{SC} [mA/cm^2]	FF [%]	a)PCE [%]
As cast	0.906 (0.902±0.004)	24.54 (24.51±0.03)	67.98 (66.24±1.74)	15.12 (14.65±0.47)
CS2,20s,90,5min	0.888 (0.882±0.005)	25.22 (25.19±0.02)	70.73 (69.79±0.93)	15.84 (15.52±0.32)
CS2,30s,90,5min	0.887 (0.884±0.003)	24.86 (25.10±0.24)	71.93 (69.69±2.23)	15.87 (15.46±0.41)
CS2,40s,90,5min	0.891 (0.883±0.007)	25.10 (25.11±0.01)	71.73 (69.90±1.83)	16.04 (15.51±0.54)
CS2,50s,90,5min	0.887 (0.884±0.003)	25.14 (25.10±0.03)	70.78 (69.88±0.89)	15.79 (15.50±0.29)

a) The average parameters were calculated over 10 independent cells.

Table S23. The photovoltaic data of the PM6:BTP-m-4Cl:BTP-o-4Cl (the ratio is 1:0.2:1.1) solar cells with different conditions. All data were obtained under the illumination of AM 1.5G ($100mW/cm^2$) light source.

Condition D: A1: A2 (1:0.2:1.1)	V_{OC} [V]	J_{SC} [mA/cm^2]	FF [%]	a)PCE [%]
As cast	0.906 (0.901±0.005)	23.92 (23.98±0.05)	67.66 (65.55±2.11)	14.66 (14.17±0.49)
CS2,20s,90,5min	0.882 (0.880±0.002)	25.19 (25.11±0.08)	70.40 (68.50±1.90)	15.64 (15.14±0.50)
CS2,30s,90,5min	0.885 (0.882±0.003)	24.16 (24.11±0.05)	71.38 (68.56±2.82)	15.25 (14.59±0.66)
CS2,40s,90,5min	0.886 (0.885±0.001)	24.54 (24.45±0.09)	70.14 (68.53±1.61)	15.25 (14.84±0.41)
CS2,50s,90,5min	0.883 (0.882±0.001)	24.13 (24.16±0.03)	68.70 (67.42±1.48)	14.68 (14.37±0.31)

a) The average parameters were calculated over 10 independent cells.

Table S24. Under optimal conditions, the PM6:BTP-m-4Cl, PM6:BTP-o-4Cl, and PM6:BTP-m-4Cl: BTP-o-4Cl photovoltaic data with different HTL interface layer (PEDOT:PSS and 2Br-2Pac). All data were obtained under the illumination of AM 1.5G (100mW/cm^2) light source.

Active layer	Interface layer	V_{OC} [V]	J_{SC} [mA/cm^2]	FF [%]	[a]PCE [%]
[b] BTP-m-4Cl	PEDOT: PSS	0.858 (0.851±0.007)	26.44 (26.40±0.04)	73.90 (71.96±1.94)	16.77 (16.18±0.59)
	2Br-2Pac	0.851 (0.850±0.001)	26.86 (26.68±0.19)	74.72 (73.09±1.62)	17.12 (16.59±0.53)
[c] BTP-o-4Cl	PEDOT: PSS	0.908 (0.897±0.011)	24.11 (23.95±0.16)	73.20 (73.07±0.12)	16.03 (15.72±0.32)
	2Br-2Pac	0.890 (0.885±0.005)	24.68 (24.45±0.23)	72.99 (71.37±1.62)	16.03 (15.44±0.59)
[d] BTP-m-4Cl: BTP-o-4Cl	PEDOT: PSS	0.867 (0.855±0.012)	26.71 (26.76±0.05)	75.50 (73.51±1.99)	17.49 (16.81±0.67)
	2Br-2Pac	0.864 (0.857±0.007)	27.37 (26.99±0.38)	75.90 (74.70±1.21)	17.95 (17.27±0.68)

a) The average parameters were calculated over 15 independent cells. b) The PM6:BTP-m-4Cl optimal conditions is CS_2, 40s, 90, 5min with ratio 1:1.3; c) The PM6:BTP-o-4Cl optimal condition is CS_2, 60s, 90, 5min with ratio 1:1.3 and c) The PM6:BTP-m-4Cl:BTP-o-4Cl optimal condition is CS_2, 30s, 90, 5min with ratio 1:1:0.3.

Table S25. Under optimal conditions, the photovoltaic performance data of inverted devices of PM6:BTP-m-4Cl, PM6:BTP-o-4Cl, and PM6:BTP-m-4Cl:BTP-o-4Cl. All data were obtained under the illumination of AM 1.5G (100mW/cm^2) light source.

Active layer	V_{OC} [V]	FF [%]	J_{SC} [mA/cm^2]	PCE [%]
PM6:BTP-m-4Cl	0.854(0.857±0.003)	71.45(70.11±1.34)	26.50(26.27±0.24)	16.11(15.72±0.39)
PM6:BTP-o-4Cl	0.884(0.880±0.004)	71.61(69.20±2.41)	24.61(24.75±0.15)	15.51(15.00±0.51)
PM6:BTP-m-4Cl: BTP-o-4Cl	0.866(0.862±0.003)	71.22(70.54±0.68)	26.93(26.38±0.55)	16.55(16.00±0.55)

a) The average parameters were calculated over 15 independent cells. b) Inverted devices with structure ITO/ZnO/active layer/MoO_3/Ag; c) The PM6:BTP-m-4Cl optimal conditions is CS_2, 40s, 90, 5min with ratio 1:1.3; d) The PM6:BTP-o-4Cl optimal condition is CS_2, 60s, 90, 5min with ratio 1:1.3 and e) The PM6:BTP-m-4Cl:BTP-o-4Cl optimal condition is CS_2, 30s, 90, 5min with ratio 1:1:0.3.

9. References

[1] G. F. Burkhard, E. T. Hoke, M. D. McGehee, *Adv. Mater.* **2010**, 22, 3293.

[2] Y. Gong, Z. Kan, W. Xu, Y. Wang, S. H. AlShammari, F. Laquai, W.-Y. Lai, W. Huang, *Solar RRL* **2018**, 2.

[3] S. H. Liao, H. J. Jhuo, Y. S. Cheng, S. A. Chen, *Adv Mater* **2013**, 25, 4766.

[4] M. Zhang, X. Guo, W. Ma, H. Ade, J. Hou, *Adv Mater* **2015**, 27, 4655.

[5] M. M. Wienk, J. M. Kroon, W. J. Verhees, J. Knol, J. C. Hummelen, P. A. van Hal, R. A. Janssen, *Angew Chem Int Ed Engl* **2003**, 42, 3371.

[6] Y. Lin, J. Wang, Z. G. Zhang, H. Bai, Y. Li, D. Zhu, X. Zhan, *Adv Mater* **2015**, 27, 1170.

[7] W. Zhao, S. Li, H. Yao, S. Zhang, Y. Zhang, B. Yang, J. Hou, *J Am Chem Soc* **2017**, 139, 7148.

[8] M. Wang, J. Li, X. Ma, J. Lv, C. Zhang, Y. Xia, *Polym. Adv. Technol.* **2018**, 29, 914.

[9] J. Yuan, Y. Zhang, L. Zhou, G. Zhang, H.-L. Yip, T.-K. Lau, X. Lu, C. Zhu, H. Peng, P. A. Johnson, M. Leclerc, Y. Cao, J. Ulanski, Y. Li, Y. Zou, *Joule* **2019**, 3, 1140.

[10] K. Jiang, Q. Wei, J. Y. L. Lai, Z. Peng, H. K. Kim, J. Yuan, L. Ye, H. Ade, Y. Zou, H. Yan, *Joule* **2019**, 3, 3020.

[11] C. Li, J. Zhou, J. Song, J. Xu, H. Zhang, X. Zhang, J. Guo, L. Zhu, D. Wei, G. Han, J. Min, Y. Zhang, Z. Xie, Y. Yi, H. Yan, F. Gao, F. Liu, Y. Sun, *Nature Energy* **2021**, 6, 605.

[12] Y. Cui, H. Yao, J. Zhang, K. Xian, T. Zhang, L. Hong, Y. Wang, Y. Xu, K. Ma, C. An, C. He, Z. Wei, F. Gao, J. Hou, *Adv Mater* **2020**, 32, e1908205.

[13] Y. Cui, H. Yao, L. Hong, T. Zhang, Y. Tang, B. Lin, K. Xian, B. Gao, C. An, P. Bi, W. Ma, J. Hou, *Natl Sci Rev* **2020**, 7, 1239.

[14] a) R. Po, G. Bianchi, C. Carbonera, A. Pellegrino, *Macromolecules* **2015**, 48, 453; b) J. Guo, B. Qiu, D. Yang, C. Zhu, L. Zhou, C. Su, U.-S. Jeng, X. Xia, X. Lu, L. Meng, Z. Zhang, Y. Li, *Advanced Functional Materials* **2022**, 32, 2110159.
